# Reduction in PA28αβ activation in HD mouse brain correlates to increased mHTT aggregation in cell models

**DOI:** 10.1371/journal.pone.0278130

**Published:** 2022-12-27

**Authors:** Karlijne W. Geijtenbeek, Jolien Janzen, Aleksandra E. Bury, Alicia Sanz-Sanz, Ron A. Hoebe, Marie K. Bondulich, Gillian P. Bates, Eric A. J. Reits, Sabine Schipper-Krom

**Affiliations:** 1 Amsterdam UMC Location University of Amsterdam, Medical Biology, Amsterdam, The Netherlands; 2 Department of Neurodegenerative Disease, Huntington’s Disease Centre and UK Dementia Research Institute at UCL, Queen Square Institute of Neurology, UCL, London, United Kingdom; Universitair Medisch Centrum Groningen, NETHERLANDS

## Abstract

Huntington’s disease is an autosomal dominant heritable disorder caused by an expanded CAG trinucleotide repeat at the N-terminus of the *Huntingtin* (*HTT*) gene. Lowering the levels of soluble mutant HTT protein prior to aggregation through increased degradation by the proteasome would be a therapeutic strategy to prevent or delay the onset of disease. Native PAGE experiments in *Hdh*Q150 mice and R6/2 mice showed that PA28αβ disassembles from the 20S proteasome during disease progression in the affected cortex, striatum and hippocampus but not in cerebellum and brainstem. Modulating PA28αβ activated proteasomes in various *in vitro* models showed that PA28αβ improved polyQ degradation, but decreased the turnover of mutant HTT. Silencing of PA28αβ in cells lead to an increase in mutant HTT aggregates, suggesting that PA28αβ is critical for overall proteostasis, but only indirectly affects mutant HTT aggregation.

## Introduction

Huntington’s disease (HD) is an autosomal dominant heritable disorder and is characterized by progressive motor dysfunction, cognitive impairment and disturbances in behavior [1,2]. The worldwide prevalence of HD is 2–5 per 100,000 people [3]. HD is caused by an expanded CAG trinucleotide repeat at the 5’ end of the *Huntingtin* (*HTT*) gene and the length of the resulting polyglutamine (polyQ) repeat in the HTT protein is inversely correlated with the age of onset of the disease [4–6]. CAG repeat lengths between 36 and 39 do not always cause signs and symptoms of HD (due to reduced penetrance) but *HTT* containing 40 or more CAG repeats leads invariably to HD [5,7]. The expanded polyQ stretches result in misfolded mutant huntingtin (mHTT) proteins by the formation of β-sheets, and N-terminal mHTT fragments containing the polyQ expansion are aggregation prone [1,8].

Lowering the levels of soluble mHTT prior to aggregation through increased degradation would be a therapeutic strategy to prevent or delay the onset of disease. The two main systems responsible for protein degradation in the eukaryotic cell are autophagy and the ubiquitin proteasome system (UPS) (reviewed by Soares et al. [9]). Whereas autophagy functions mainly in the cytoplasm, the UPS is the main route for the degradation of misfolded proteins in both the cytoplasm and the nucleus [10–12]. The UPS is thus a promising candidate to target both cytoplasmic and nuclear mHTT fragments. Initial studies suggested that the proteasome cannot degrade polyQ stretches and that proteasomes are irreversibly sequestered into mHTT aggregates [13–17]. More recently, we showed that proteasomes are dynamically recruited to mHTT inclusion bodies, that they remain catalytically active and that they are accessible to substrates [18]. Furthermore, when polyQ-expanded mHTT fragments were targeted towards the proteasome with an N-terminal degradation signal, they were efficiently and completely degraded [19]. Together this indicates that the proteasome remains active in cells with HTT inclusion bodies and is able to degrade soluble mHTT when this is targeted for degradation.

The proteasome consists of a latent 20S core particle, which is composed of four heptameric α- and β-rings (α_7_β_7_β_7_α_7_), of which the latter ones contain catalytic subunits that face the interior of the cylinder and are responsible for the proteolytic activity [20,21]. Access to the central cavity is regulated by a gate formed by the N-terminal protrusions of the α-subunits [22]. Modulation of the gate is required for substrate entry into the 20S core and is mediated by the proteasome activators (PA), such as the 19S complex and PA28. The 19S and PA28 can bind to both ends of the 20S proteasome core leading to single (referred to as 26S when one 19S complex binds 20S), double (referred to as 30S when two 19S complexes bind 20S) or even hybrid-capped proteasomes (with a 19S and PA28 complex on opposing ends of the 20S) [23]. The 19S is a complex of distinct subunits and is involved in the recognition, unfolding and de-ubiquitination of poly-ubiquitinated substrates in an ATP-dependent manner [24]. PA28 is involved in ATP-independent degradation and has three homologues: PA28α and PA28β, forming heterodimers, and PA28γ, forming a homo-heptameric ring, which is only expressed in the nucleus [25,26]. Although PA28αβ exists as a heterodimer, homomeric PA28α is sufficient to activate proteasome activity [26,27]. By binding to the proteasomal α-ring, PA28 allows entrance of peptides into the 20S core and increases proteasome activity. PA28 overexpression also increases degradation of oxidized proteins in cells, with increased PA28αβ binding to the 20S proteasome immediately upon H_2_O_2_ treatment, followed by increased PA28αβ expression during oxidative stress adaptation [28–30]. Interestingly, PA28αβ has been shown to prevent aggregation in the mouse hippocampus during aging [31,32]. Furthermore, PA28γ overexpression relieved HD pathology and lowered the number of inclusion bodies in cells and *in vivo* [33,34]. The role of PA28αβ, which is present in both the nucleus and cytoplasm, on mHTT turnover remains elusive.

In this study, we examined whether changes in proteasome complex formation occur during disease progression in HD mouse models. To determine which consequences these alterations have on mHTT degradation, we modulated PA28αβ activated proteasomes in various *in vitro* models and determined the effects on both polyQ fragments and mHTT proteins.

## Materials and methods

### HD mouse models

All procedures were in accordance with the Animals (Scientific Procedures) Act 1986 and were approved by the King’s College London (KCL) Ethical Review Process Committee. In this study *Hdh*Q150 mice [35] expressing endogenous full-length mouse *Htt* with an expanded CAG repeat and R6/2 mice [36] expressing a human exon 1 *HTT* transgene were used. Animals were genotyped by PCR and CAG length was determined as previously described [37]. The *Hdh*Q150 homozygous mice were on a CBA/Ca and C57BL/6J F1 background and had a CAG repeat size of 166±9. The R6/2 mice were bred by backcrossing R6/2 males (CBA/Ca x C57BL/6J) to F1 females (B6CBAF1/OlaHsd, Envigo, Netherlands) and had a CAG repeat length of 209±2. *Hdh*Q150 mice were sacrificed by cervical dislocation at 2, 16 and 22 months of age, and R6/2 mice at 4 and 14 weeks of age. The dissected brain regions were immediately snap frozen.

### DNA constructs

PA28α and PA28β (kindly provided by Prof. PM Kloetzel, Charité Universitätsmedizin Berlin, Germany) were cloned into a pcDNA3 vector using EcoRI. GFP-Ub-Q54, DNAJB6 and m*HTT*(Q25/Q97)exon1-H4 were generated as described before [19,38,39], respectively.

To create a stable cell line with inducible expression of mHTT, a pINDUCER m*HTT*(Q25/Q46/Q97)exon1-IRES-*GFP*-Q16 construct was generated. First, m*HTT*(Q97)exon1 was amplified with Xho on the 3’ end (fw (T7) 5’-TAATACGACTCACTATAGGG-’3, rv 5’-GTTCTAGATTAAGGTCGGTGCAGAGGCTC-’3) and cloned into pIRES2-*GFP*, using XhoI and SmaI, creating m*HTT*(Q97)exon1-IRES-*GFP*. Next, *GFP*-Q16 was amplified with BstX1 and Not1 on the 3’ and 5’ ends, respectively (fw 5’-CGATGATAATATGGCCACAACCATGGCCACCATGGTGAGCAAGGGCGAGG-3’, rv 5’-TGATCTAGAGTCGCGGCCCCGCTTACCTGGGGCTAGTCTC-3’) and cloned into m*HTT*(Q97)exon1-IRES-*GFP* using BstX1 and Not1, to replace *GFP*. Subsequently, m*HTT*(Q97)exon1-IRES-*GFP*-Q16 was amplified with EcoRI on the 3’ and 5’ ends (fw 5’-GTCCAGTGTGGTGGAATTCTCGAGGTCGACCGCCATGG-’3, rv 5’- CTGGATATCTGCAGaattCCGCTTACCTGGGGCTAGTCTC-‘3) and introduced into pENTR/D-TOPO by EcoRI. Finally, the construct was transferred from the pENTER Gateway vector into the pINDUCER20-Blast lenti-viral dox-regulated expression vector. To create cells expressing m*HTT*(Q25/Q46)exon1-IRES-*GFP*-Q16 m*HTT*(Q25/Q46)exon1 was amplified with BamHI on the 3’ and 5’ ends (fw 5’-TGGTACCGAGCTCGGATCGCCACCATGGCGACCCTGGAAAAGCTG-’3, rv 5’-GAGGGAGAGGGGCGGATCTTAAGGTCGGTGCAGAGGCTC-’3). Next m*HTT*(Q25/Q46)exon1 was cloned into pENTR m*HTT*(Q25/Q46)exon1-IRES-*GFP*-Q16 using BamHI, replacing m*HTT*(Q97)exon1. All plasmids were verified by sequencing before use.

### Cell culture

HEK293 and ST*Hdh*^Q7/Q7^ [40] cells were cultured in DMEM (Gibco) supplemented with 10% FBS (Gibco), 1% penicillin/streptomycin (Gibco) and 0.2mM L-glutamine (Gibco) and grown in a humidified chamber with 5% CO_2_ at 37°C or 32°C, respectively. ST*Hdh* cells expressing doxycycline inducible m*HTT* were generated by retroviral transfection of ST*Hdh*^Q7/Q7^ cells with pINDUCER m*HTT*(Q25/Q46/Q97)exon1-IRES-*GFP*-Q16.

### Treatment and transfection

HEK293 cells were stimulated with 100U/ml IFNγ (ProSpec) for 72 hours and proteasomes were inhibited with 250nM epoxomicin (Sigma) for 16 hours. HEK293 cells were transfected with jetPEI one day after plating according to the manufacturer’s instructions (Polyplus transfection). Neon Transfection System (Invitrogen) was used to overexpress constructs in ST*Hdh* cells. For silencing experiments with shRNA, the MISSION® TRC-Mm 1.0 (Mouse) library was used. The PA28α targeting sequence (5’-CCCGATCCAGTCAAAGAGAAA-3’, MISSION® TRC shRNA TRCN0000066420) was delivered through retroviral transduction. The pLKO.1-puro Non-Mammalian shRNA Control Plasmid (SHC002) was used as a control. SiRNA targeting PA28α (ON-TARGETplus Mouse Psme1 siRNA SmartPool, Horizon Discovery) or non-targeting siRNA (siGENOME Non-Targeting siRNA Control Pool, Horizon Discovery) was delivered to the cells by using Lipofectamine RNAiMAX Transfection Reagent (Invitrogen) directly with plating the cells.

### Native PAGE

Brain tissue and cell pellets were suspended in TSDG buffer (10mM Tris/HCl pH7.4, 25mM KCl, 10mM NaCl, 1.1mM MgCl_2_, 0.1mM EDTA, 10% glycerol, 1mM ATP fresh) and brain tissue was further homogenized by the use of Dounce tissue homogenizer. Lysis was performed by 3–5 freeze/thaw cycles in liquid nitrogen. After centrifugation (15 minutes, 14.000 rpm at 4°C), the protein concentration of the clarified lysate was determined by Bradford protein assay (Serva). After the addition of 4x native sample buffer (20mM Tris pH8.0, 50% glycerol, bromophenol blue) samples were separated on 3–12% NativePAGE Novex Bis-Tris gels (Invitrogen). For western blotting, native gels were transferred to PVDF membranes (Millipore, Bedford, MA, USA) in transfer buffer (25mM Tris pH7.5, 192mM Glycine, 20% MeOH) using the Criterion blotter (Biorad). After blocking in 5% milk, membranes were incubated with the antibody of interest and Odyssey detection system (LICOR Bioscienses) was used for scanning and analysis.

### Visualizing proteasome activity and peptide degradation in gel

Activity based probe (ABP) labeling was performed either in the lysate or after running native PAGE [41]. For in-lysate labeling, the samples were incubated with 0.5μM ABP for 30 minutes at 37°C before adding sample buffer and loading on native PAGE. In-gel ABP labeling or in-gel peptide degradation was performed after protein separation by native PAGE. For these overlay assays the wet gel slab was incubated in 10ml overlay buffer (20mM Tris pH7.5, 5mM MgCl_2_, 1mM ATP fresh) with 25nM ABP or 400μM of the quenched peptide for 20 minutes at 37°C. Fluorescent intensities were measured directly on a Typhoon imager (Ge Healthsciences) using the 580 BP 30 filter. In order to inhibit proteasome activity in these assays, the lysate was incubated with 0.5μM epoxomicin (Sigma), or similar amounts of DMSO in control samples, for 1 hour at 37°C, prior to in lysate labeling or native PAGE separation.

### Western blot

Cells pellets were lysed in Triton-x buffer (50mM Tris/HCl pH7.4, 150mM NaCl, 1mM EDTA, 1% Triton-X100, supplemented with complete mini protease inhibitor cocktail (Roche)). After centrifugation (15 minutes, 14.000 rpm at 4°C), the protein concentration of the supernatant was determined by Bradford protein assay (Serva). Samples were boiled in 6x sample loading buffer (350mM Tris/HCl pH6.8, 10% SDS, 30% glycerol, 6% β-mercaptoethanol, bromophenol blue) and separated on 12.5% SDS-PAGE gels. Proteins were transferred to nitrocellulose membranes (Biorad) with the use of the Trans-Blot Turbo Transfer System (Biorad). After blocking in 5% milk, membranes were incubated with the antibody of interest and Odyssey detection system (LICOR Bioscienses) was used for scanning and analysis.

### Filtertrap assay

For filtertrap assay, the pellet obtained after centrifugation of the cell lysate was resuspended and treated with endonucleases for 1 hour at 37°C (1mM MgCl_2_, 50mM Tris/HCl pH8.0, with 0.02U/μl DENARASE® (c-LEcta) added fresh). This reaction was stopped by adding 2x termination buffer (40mM EDTA, 4% SDS, 100mM DTT fresh) and samples were diluted in 2% SDS buffer (2% SDS, 150mM NaCl, 10mM Tris/HCl pH8.0). Cellulose acetate membranes (Schleicher & Schuell) with a pore size of 0.2 μm were pre-equilibrated in 2% SDS buffer. After sample loading through the Bio-Dot microfiltration apparatus (Biorad, Hercules, CA, USA), the membrane was washed twice with 0.1% SDS buffer (0.1% SDS, 150mM NaCl, 10mM Tris pH8.0) and further treated like western blot membranes.

### Antibodies

The following primary antibodies were used: anti-PA28α, directed against RVQPEAQAKVDVFRED, (1:3000, kindly provided by Prof. M Groettrup, University of Konstanz, Germany) [100], anti-PA28α (1:1000, Enzo Life Sciences, BML-PW8185), anti-polyQ 1C2 (1:1000, Millipore, MAB1574), anti-polyQ (1:1000, Sigma-Aldrich 3B5H10, P1874), anti-β-actin (1:1000, Santa Cruz, SC-130656 and SC-47778), anti-α2 (1:1000, MCP236, kindly provided by Prof. Rasmus Hartmann-Petersen, Biologisk Institut, University of Copenhagen, Copenhagen), anti-α7 (1:1000, MCP72, Enzo Life Sciences, PW8110), anti-HTT (1:5000, Abcam, ab109115), anti-tubulin (1:1000, Cell Signaling Technology, CST2148) and anti-RPT (1:1000, Enzo Life Sciences, PW8825). IRDye 680 and IRDye 800 (1:10.000; LI-COR Biosciences) were used as secondary antibodies.

### In vitro degradation assays

mHTT(Q25/Q97)exon1-H4 was purified as described before [19]. 100ng purified HTT(Q97)exon1-H4 protein was incubated with 0.3μg mammalian open-gated 20S proteasomes in 1x 20S buffer (10mM Tris/HCl pH7.4, 30mM NaCl, 1mM MgCl2, 400μM DTT fresh) for 8 hours at 37°C. For proteasome inhibition, 1μM epoxomicin (Sigma), or similar amounts of DMSO in control samples, was added. For proteasome activation, 3μg isolated PA28αβ caps, 50μM RPT peptides (both kindly provided by Prof. M. Rechsteiner, University of Utah School of Medicine, USA) or 0.01% SDS were added to the reaction. ATP Regeneration solution (Enzo Life Sciences) was added to reactions involving the 26S proteasome. After the incubation period, 0.5μM ABP was added for an additional 30 minutes at 37°C. The reaction was stopped by boiling the samples in 6x sample loading buffer (350mM Tris/HCl pH6.8, 10% SDS, 30% glycerol, 6% β-mercaptoethanol, bromophenol blue). The complete reaction was used for 12.5% SDS PAGE to visualize ABP signal and for immunoblotting.

### Fluorescent microscopy and quantification of aggregate formation

STHdh mHTT(Q97)exon1-IRES-GFP-Q16 cells were rinsed with PBS and fixed with 4% formaldehyde in 1x PBS for 1 hour at RT. Nuclei were stained with 0.01 mg/ml Hoechst 33324. Images were obtained using automated microscopy (ImageXpress Pico). To measure the aggregates, GFP-Q16 was used as a fluorescent reporter. The amount of aggregation was defined by using the MATLAB algorithm. In short, the algorithm defined the single nuclei on the pictures and it calculated the average intensity of the nuclei, which was later used to calculate the amount of cells on the images. Nuclei were defined based on size, intensity and shape. Aggregates were defined based on their size and intensity that needs to be above certain threshold based on the GFP overexpression in the cells. More information on the MATLAB script can be found as supplementary information.

### Data quantification

Experimental results were normalized to the control condition and presented as mean ± SEM. Outliers were identified using Grubbs’ test statistic, and removed. Statistical differences between groups were determined by the one sample t-test or one-way ANOVA with Dunnett’s Multiple Comparison. Analysis was performed using GraphPad™ Prism v.9 (GraphPad Software, Inc.). An alpha level of 0.05 was used to define statistical significance. All data is available through the online platform FiglinQ (https://create.figlinq.com/~k.geijtenbeek/92/collection/).

## Results

### Loss of PA28 activated proteasomes during HD progression in HD mice

**Fig 1:**
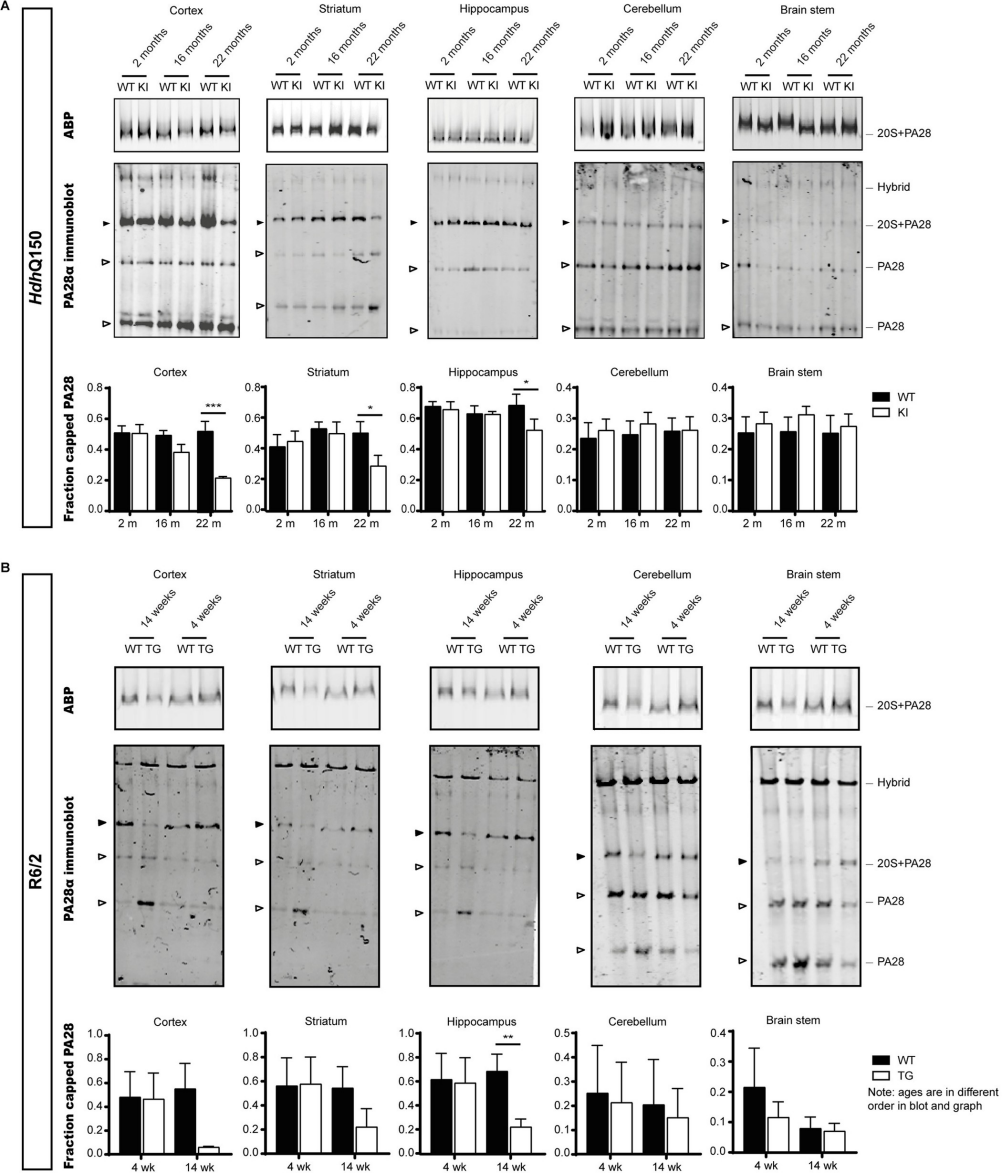
Loss of PA28αβ capped proteasomes during HD progression. Native PAGE showing proteasome activity labeling and PA28α immunoblot for different brain regions of *Hdh*Q150 (A) and R6/2 (B) during disease progression. The upper blots depict fluorescent ABP signal, only showing the bands representing PA28 capped proteasomes. The graphs in the lower panels show quantification of the fraction of capped PA28αβ (PA28α bound to 20S, black arrowheads, divided by the total pool of PA28α (black and open arrowheads). Data are shown as mean ± SEM (*Hdh*Q150 n = 3/4; R6/2 n = 3/4). A one sample t-test is performed after normalization to the WT animal of the same age-group (p-values are only given for the latest age group). One sample t-test; *Hdh*Q150 22m cortex p = 0.0006; striatum p = 0.0269; hippocampus p = 0.0119; cerebellum p = 0.8258; brain stem p = 0.3801; R6/2 14wk cortex p = 0.1620; striatum p = 0.0546; hippocampus p = 0.0052; cerebellum p = 0.5529; brain stem p = 0.4152. * indicates p<0.05; ** indicates p<0.01; *** indicates p<0.001. WT = wildtype; KI = knock-in; TG = transgene; m = months; wk = weeks.

To explore whether proteasome complex formation changes during HD development, we investigated proteasome complex composition in two different HD mouse models: the *Hdh*Q150 mice [35] expressing endogenous full-length mouse *Htt* with an expanded CAG repeat and the R6/2 mice [36] expressing a human exon 1 *HTT* transgene. For the visualization of proteasome complexes, we used ABP labeling [42]. These probes bind to the catalytic sites of 20S proteasomes and can be visualized via their fluorescence tag. Only proteasome complexes associated with proteasome activating complexes such as PA28 and the 19S are accessible for substrates. Therefore, ABPs will specifically label incorporated, active proteasome subunits [41]. When the lysates are subsequently separated on native PAGE gels to keep protein complexes intact, and are analyzed for fluorescence, various bands can be distinguished representing various proteasome complexes (S1 Fig). To represent different disease stages, proteasome complexes were analyzed at 2, 16 and 22 months of age for homozygous *Hdh*Q150 mice (Fig 1A), and at 4 and 14 weeks of age for R6/2 mice (Fig 1B), which show a faster disease progression due to the expression of the N-terminal exon 1 HTT fragment. Of these mice cortex, striatum, hippocampus, cerebellum and brain stem were dissected and analyzed, with HD pathology being more prominent in the first regions. The frozen sections were lysed, labeled with ABP and subjected to native PAGE analysis for fluorescence gel analysis. A decrease in ABP labeling at the height of PA28 capped proteasomes was observed in late disease stages in various brain regions, which suggests an alteration in these complexes (Fig 1A and 1B, upper panels showing ABP labeling). Interestingly, immunostaining of PA28α subunits confirmed a decrease in PA28αβ activated proteasomes (Fig 1A and 1B, middle panels, black arrowheads). In addition, an increase in the free pool of PA28αβ subunits (not bound to 20S proteasomes) was observed in late stage models (Fig 1A and 1B, open arrowheads at the bottom of the gel), suggesting disassembly of the PA28αβ activator from the 20S core. Quantification of the fraction capped PA28αβ (black arrowheads) versus the total pool of PA28α (black and open arrowheads) showed a significant decrease in PA28αβ proteasome activation at late disease stages in cortex, striatum and hippocampus of *Hdh*Q150 mice, and in hippocampus of R6/2 mice (Fig 1A and 1B, graphs in lower panels).

### PA28αβ overexpression improves degradation of polyQ peptides

**Fig 2:**
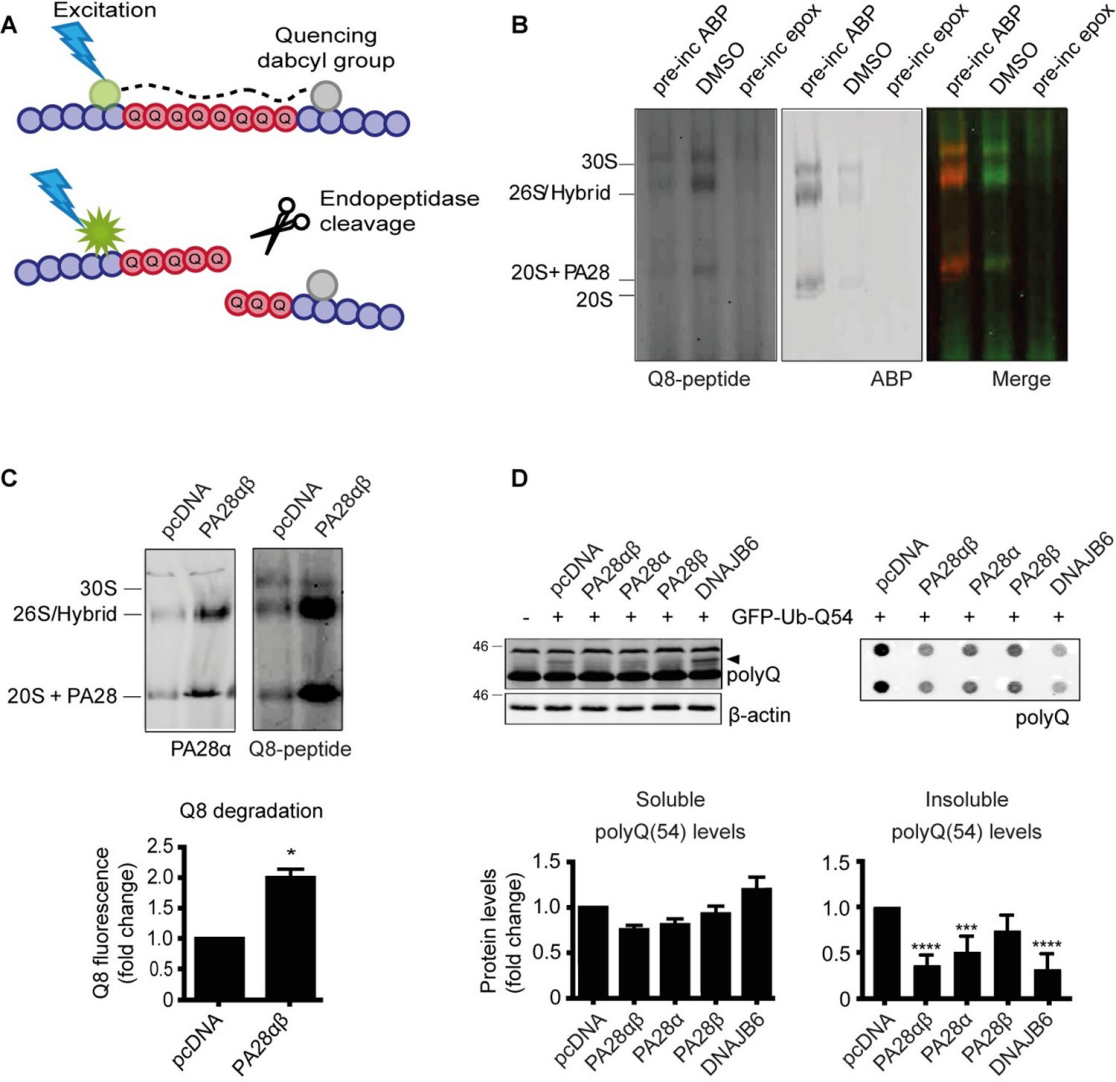
Addition of PA28αβ increases degradation of polyQ peptides. (A) Illustration of qQ8 peptides, which are used to show polyQ endopeptidase activity. These peptides include eight glutamine residues (represented in red) which are flanked by non-degradable D-amino acids (represented in purple) at both peptide termini to prevent degradation by exopeptidases. Upon cleavage within the polyQ sequence, the C-terminal quencher (in grey), and N-terminal fluorophore (in green), are separated which will result in the emission of fluorescent signal. (B) Proteasome activity labeling by ABP and qQ8-peptide degradation on native PAGE. (C) Native PAGE showing improved polyQ degradation after PA28αβ overexpression in HEK293 cells. Data are normalized to WT HEK cells and shown as mean ± SEM (n = 3). One-sample t-test; PA28αβ p = 0.0170. * p<0.05. Pre-inc: Pre-incubated. (D) PA28α and PA28αβ overexpression in HEK293 cells improves the clearance of insoluble polyQ peptides. Data are normalized to pcDNA transfected cells and shown as mean ± SEM (soluble n = 3; insoluble n = 4). One-way ANOVA with Dunnet’s multiple testing; for soluble mHTT PA28αβ p = 0.1612; PA28α p = 0.3313; PA28β p = 0.9235; DNAJB6 p = 0.3006; for insoluble mHTT PA28αβ p<0.0001; PA28α p = 0.0010; PA28β p = 0.0788; DNAJB6 p<0.0001. *** p<0.001; **** p<0.0001. Pre-inc = pre incubation; ABP = activity based probe; epox = epoxomicin.

To examine whether the observed reduction in PA28αβ proteasome activation in HD mice would affect mHTT degradation directly, we first studied the effect of PA28αβ-activated proteasomes towards degradation of polyQ repeats using quenched Q8 (qQ8) peptides [41], which become fluorescent after cleavage by endopeptidases (Fig 2A). When lysates are separated on native PAGE gels, proteasome complexes do not only remain intact, but also remain active. If these gels are subsequently incubated with the qQ8 peptides in an overlay assay, local fluorescence will appear at the height of the responsible enzyme when the peptide is cleaved. When HEK293 cell lysate were subjected to an overlay assay with the qQ8 peptide, a pattern of fluorescent bands was observed, demonstrating polyQ peptide degradation (Fig 2B, left panel). Treatment with epoxomicin prevented cleavage of the polyQ peptides, indicating that the fluorescence was specifically generated by proteasomal cleavage. In addition, ABP labeled proteasomes showed a similar fluorescence pattern (Fig 2B, middle panel), and merging both fluorescent channels demonstrated the ability of proteasomes to cleave polyQ substrates (Fig 2B, right panel). To examine the effect of PA28αβ activation on the degradation of these polyQ-peptides in cells, HEK293 cells were transfected with PA28αβ. A native PAGE overlay with qQ8 peptides showed that PA28αβ overexpression led to an increase in qQ8 degradation (Fig 2C). Together these data demonstrate that proteasomes are able to cleave within polyQ sequences and that activation by PA28αβ accelerates this degradation.

Next, we examined whether overexpression of PA28αβ would enhance the degradation of polyQ peptides that exceed the pathological threshold. We transfected HEK293 cells with GFP-Ub-Q54, which generates pure Q54-peptides since GFP-Ub is separated by C-terminal hydrolases directly after synthesis [38]. Cells were co-transfected with PA28α, PA28β or PA28αβ. The chaperone DNAJB6 was transfected as a positive control to detect soluble Q54, as it prevents aggregation of polyQ peptides [39]. Although no significant decrease in soluble mHTT levels (arrowhead) was observed, overexpression of PA28α and PA28αβ led to a decrease in insoluble Q54 levels as shown by filtertrap analysis (Fig 2D). To study whether the PA28αβ-induced effects on polyQ peptide levels were due to improved degradation through the proteasome, we treated the cells with the proteasome inhibitor epoxomicin (S2 Fig). Epoxomicin slightly reduced the effects of PA28 overexpression. The fact that epoxomicin could not completely prevent the increased degradation of Q54 by PA28αβ can be explained by the relatively short incubation with the inhibitor compared to the long expression time of PA28 and Q54. Together this shows that PA28αβ improves degradation of polyQ peptides by the proteasome.

### PA28αβ hampers mHTT degradation by the 20S in vitro

**Fig 3:**
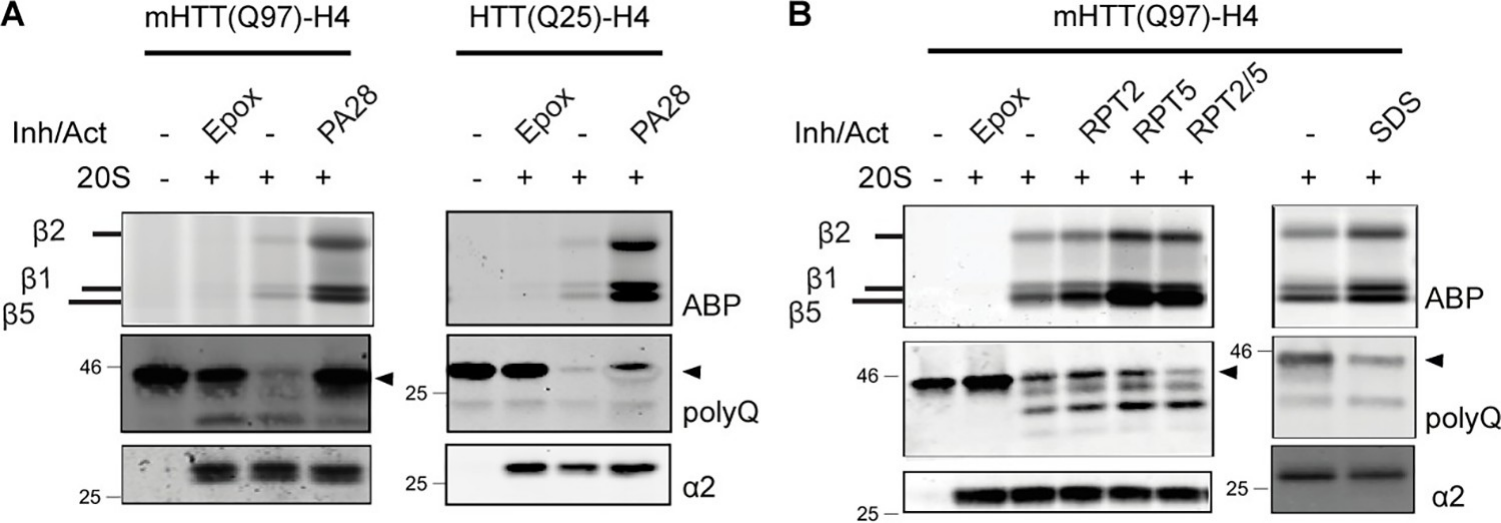
PA28αβ hampers *in vitro* degradation of mHTT by the proteasome. (A) mHTT(Q97)exon1-H4 or mHTT(Q25)exon1-H4 incubated with purified 20S proteasomes in the absence or presence of PA28αβ. Activating purified 20S proteasomes with PA28αβ prevents degradation of mHTT(Q97)exon1-H4 and reduces degradation of mHTT(Q25)exon1-H4 (arrowheads). (B) Incubation of 20S proteasomes with RPT2, RPT5, RPT2 and 5 or SDS increases proteasome accessibility and activity as shown by ABP labeling, and improves the degradation of mHTT(Q97)exon1-H4 (arrowhead). Inh = inhibitor; Act = activator; Epox = epoxomicin.

Since PA28αβ improved degradation of polyQ-expanded peptides, we next examined whether PA28αβ would also improve the degradation of polyQ-expanded mHTT protein fragments. First, we used isolated mHTT fragments to study the degradation *in vitro*. For this we isolated N-terminal mHTT(Q25/Q97)exon1 fragments from N2a cells [19]. These proteins resemble the fragments that are also expressed in the R6/2 mice. The mHTT fragments were incubated with purified 20S proteasomes in the absence or presence of isolated PA28αβ. Both HTT(Q25) and mHTT(Q97) (arrowheads) were degraded by 20S proteasomes. However, when 20S proteasomes were activated by PA28αβ, as demonstrated by ABP labeling, the degradation of HTT(Q25) was reduced and mHTT(Q97) degradation was completely prevented (Fig 3A). Interestingly, artificial opening of the 20S did result in increased mHTT(Q97) (arrowheads) degradation (Fig 3B). We stimulated 20S opening by using RPT peptides that represent the C-termini of the 19S subunits RPT2 and RPT5, which are responsible for opening of the α-ring [43,44]. Additionally, we used SDS to open 20S proteasomes [45]. It should be noted however that SDS can also affect protein denaturation and thereby its accessibility into the proteasome. Toghether these results indicate that (artificial) opening the entrance of the 20S core can improve degradation of mHTT, but that PA28αβ only improves the accessibility of the 20S for small peptides, while entrance of larger protein fragments including mHTT exon1 is completely blocked.

### PA28αβ overexpression does not affect mHTT levels in cells

**Fig 4:**
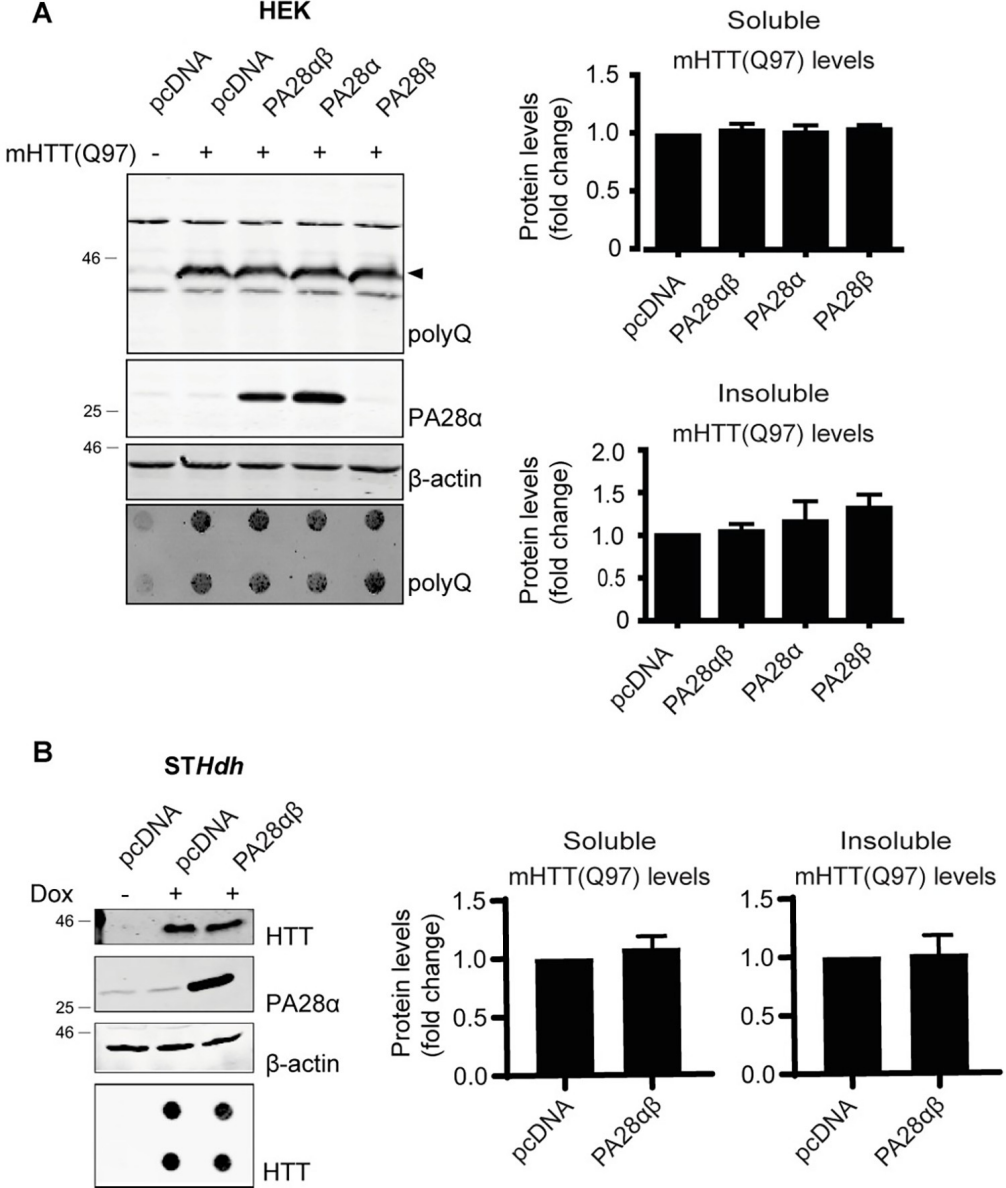
PA28αβ overexpression does not affect mHTT degradation in cells. (A) Effects of PA28α, PA28β or PA28αβ on levels of soluble (arrowhead) and insoluble mHTT(Q97) in HEK293 cells. Data are normalized to pcDNA transfected cells and shown as mean ± SEM (n = 3). One-way ANOVA with Dunnet’s multiple testing; for soluble mHTT PA28αβ p = 0.8481, PA28α p = 0.9552, PA28β p = 0.7517; for insoluble mHTT PA28αβ p = 0.9900, PA28α p = 0.7822, PA28β p = 0.3380 (B) Effects of PA28αβ on soluble and insoluble mHTT(Q97) in ST*Hdh* cells expressing dox inducible mHTT(Q97)exon1. Data are normalized to pcDNA transfected cells and shown as mean ± SEM (n = 4). One sample t-test; for soluble mHTT p = 0.47; for insoluble mHTT p = 0.82.

Following the experiments with purified proteasomes, we examined the effects of PA28αβ activation on mHTT exon1 degradation in cells, which contain all other components of the proteostasis network that may affect the role of PA28αβ on mHTT degradation [46]. HEK293 cells transfected with N-terminal mHTT(Q97) and PA28α, PA28β or PA28αβ for 72 hours showed an increase in activity of PA28αβ activated proteasomes, as shown by ABP labeling and immunoblotting, yet no statistically significant changes were observed in either soluble (arrowhead) or insoluble mHTT levels upon PA28αβ overexpression (Fig 4A). Since cell-type specific differences were observed in mHTT aggregation and sensitivity [47] and proteasome composition differs between different human cell lines [23], we next examined whether proteasomal complexes differ between HEK293 cells and striatal ST*Hdh*^Q7/Q7^ cells, which are more relevant in HD [40]. When proteasome complexes of both cell lines were analyzed by native PAGE (S3 Fig) ST*Hdh* cells showed higher levels of hybrid and 30S proteasome complexes, while the 26S proteasome is more abundant in HEK293 cells. Interestingly, PA28αβ seems to be more abundant in ST*Hdh* cells, both as a free pool and in complex with 20S proteasomes. Modulating proteasomal complexes could therefore have distinct consequences in these cell lines. To examine the effect of PA28αβ levels on mHTT degradation in ST*Hdh* cells, we generated ST*Hdh*^Q7/Q7^ cells that express N-terminal mHTT(Q97)-exon1 under a doxycycline inducible promotor. These ST*Hdh*(Q97) cells were electroporated with PA28αβ and mHTT expression was subsequently induced for 48 hours (Fig 4B). Resembling the data observed in HEK293 cells, no changes in soluble or insoluble mHTT(Q97) levels were detected after PA28αβ overexpression in ST*Hdh* cells.

### PA28αβ silencing in STHdh cells increases mHTT aggregation

**Fig 5:**
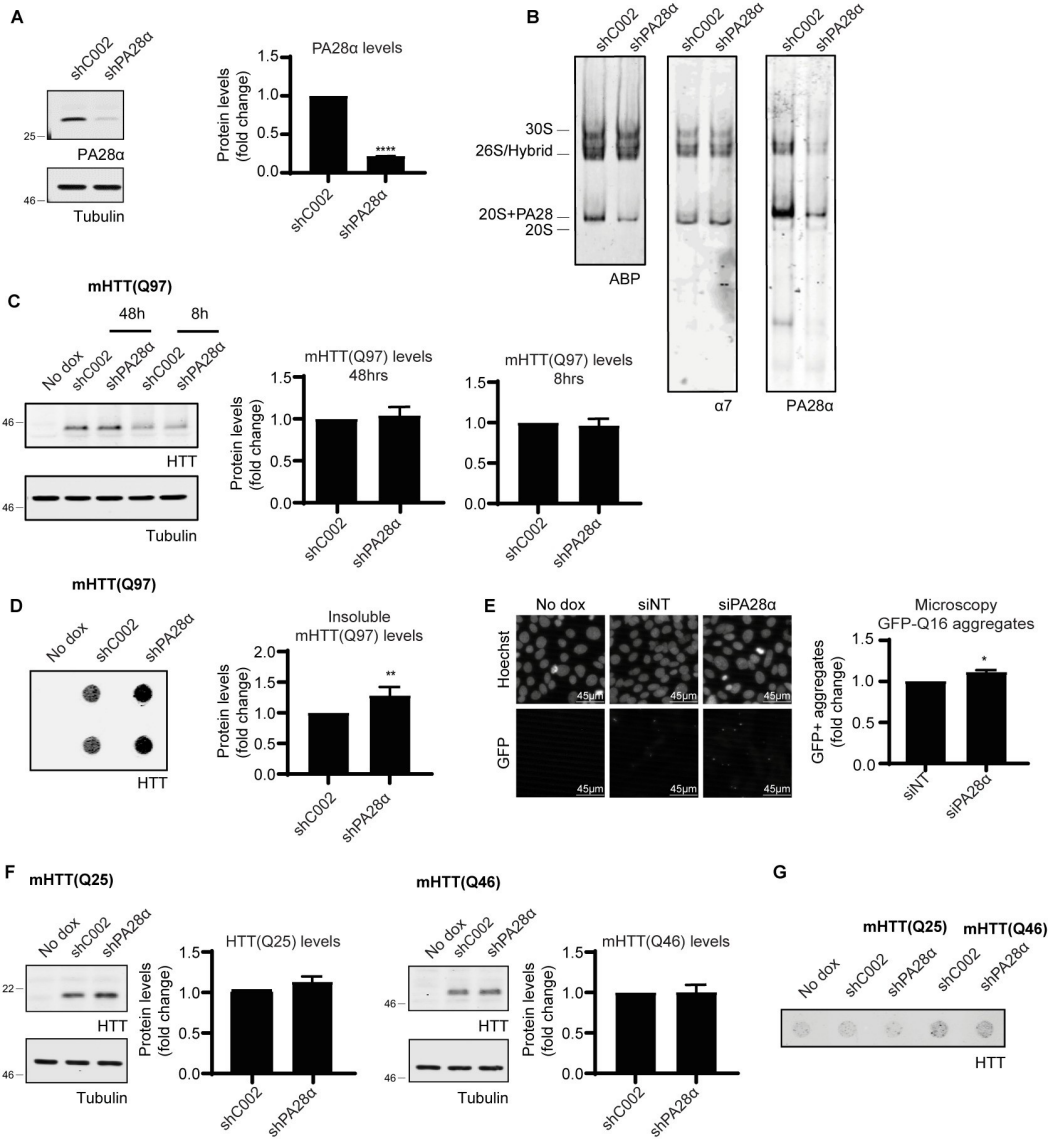
PA28αβ silencing increases mHTT aggregation in ST*Hdh* cells. (A) ST*Hdh* cells treated with shPA28α show decreased PA28α protein levels. Data are normalized to shC002 treated cells and shown as mean ± SEM (n = 6). One sample t-test; **** p<0.0001. (B) Native PAGE of PA28αβ activated proteasomes levels in ST*Hdh* cells treated with shPA28α as shown by ABP labeling and immunoblotting. (C) Effects ofPA28α silencing on soluble mHTT(Q97) levels in ST*Hdh* cells by SDS-PAGE. HTT expression was normalized to shC002 treated cells and shown as mean ± SEM (n = 3). One sample t-test; mHTT(Q97) 48h p = 0.4515, mHTT(Q97) 8h p = 0.6969. (D) Filtertrap analysis to showthe effects of shPA28α treatment in ST*Hdh* cells on mHTT(Q97) aggregation. Data are normalized to shC002 treated cells and shown as mean ± SEM (n = 7). One sample t-test; p = 0.0093. * p<0.05; ** p<0.01 (E) mHTT(97) aggregation analyzed by microscopy in ST*Hdh* cells expressing dox inducible mHTT(Q97)exon1-IRES-GFP-Q16 upon treatment with siNT or siPA28α. Data are normalized to siNT treated cells and shown as mean ± SEM (n = 4). One sample t-test; p = 0.0295. (F) Effects of transduction with shPA28α on soluble levels of wildtype HTT(Q25) and on shorter polyQ-expanded mHTT(Q46) fragments. HTT expression was quantified and normalized to shC002 treated cells and shown as mean ± SEM (n = 3). One sample t-test; HTT(Q25) p = 0.2857, mHTT(Q46) p = 0.9331. (G) Effects of PA28α silencing on HTT(Q25) and mHTT(Q46) aggregation.

To examine the effects of PA28αβ silencing on mHTT(Q97) clearance in ST*Hdh* cells that express high PA28αβ levels, and thereby mimic the decrease in PA28αβ capped proteasomes observed in HD mice, we reduced PA28α levels by 80% using retroviral transduction of shRNA targeting PA28α (Fig 5A). Native PAGE showed efficient reduction of PA28αβ activated proteasomes, also leading to less proteasome activity as shown by ABP labeling (Fig 5B). Subsequently, mHTT expression was induced for eight hours (pre-aggregation state) or 48 hours and the effects of PA28α silencing on mHTT protein levels were determined. While reduced PA28αβ activated proteasome levels did not affect soluble mHTT(Q97) protein levels (Fig 5C), levels of insoluble mHTT(Q97) increased significantly (Fig 5D). These results were confirmed using fluorescence microscopy experiments, using ST*Hdh* cells expressing doxocycline inducible mHTT(Q97)-IRES-GFPQ16 (Fig 5E). Here the separately expressed GFPQ16 acts as a fluorescent reporter for mHTT aggregation, with the short polyQ sequence being sequestered into aggregates formed by untagged mHTT exon1, while in the absence of mHTT aggregation the GFP-Q16 reporter will be diffusedly distributed throughout the cell. This approach enables the visualization of aggregation of untagged mHTT in cells, which is preferred since a tag can influence a protein’s stability. Following siRNA transfection targeting PA28α and induction of mHTT expression, the cells were analyzed by automated microscopy and the percentage of cells with aggregates was determined by a generated Matlab script. This data showed that, similar to the filtertrap assay, PA28α silencing increased aggregation. When HTT(Q25) or mHTT(Q46) were used as wildtype or short polyQ-expanded HTT fragments, respectively, which do not form aggregates after 48 hours of expression, no effects of PA28α silencing on soluble wildtype HTT or mHTT levels were observed (Fig 5F). To investigate whether PA28α silencing leads to accelerated aggregation HTT(Q25) and mHTT(Q46) samples were analyzed in a filtertrap assay (Fig 5G). Although five times more protein was loaded for HTT(Q25) and mHTT(Q46) compared to mHTT(Q97), there was no signal above background visible. This indicates that there were no aggregates present, despite silenced PA28α levels. Altogether this data indicates that downregulation of PA28αβ accelerates aggregate formation, but does not affect soluble HTT turnover directly.

## Discussion

By studying changes in proteasome complexes during disease progression in HD mouse models, we observed that PA28αβ disassembles from the 20S core most obviously in the cortex, striatum and hippocampus during HD disease progression. This suggests that the changes in PA28αβ were most abundant in the HD-affected regions. When examining the consequences of alterations in PA28αβ activated proteasomes on mHTT turnover in cell models and using purified proteasomes, we observed that while the degradation of polyQ peptides is improved by PA28αβ activation of (purified) proteasomes, degradation of the mHTT protein fragment is hampered by the addition of PA28αβ to purified proteasomes. This shows that PA28 obstructs the entrance of the folded mHTT protein into the 20S core. Indeed it has been suggested before that PA28αβ selectively blocks the passage of larger protein fragments [48]. However, PA28αβ overexpression did not affect mHTT levels when overexpressed in cells. This can suggest several things: PA28αβ activation is already sufficient; another limiting factor necessary for degradation is required; other proteasome complexes including the 26S proteasome mainly target mHTT; or mHTT is not efficiently targeted towards the proteasome [19]. However, as reducing PA28αβ levels increased mHTT aggregation but did not affect the degradation of soluble (m)HTT, this suggests that PA28αβ is critical for overall proteostasis and only indirectly affects mHTT aggregation.

The indirect role of PA28αβ affecting mHTT aggregation may be the result of its reported chaperone-like functions. The 90-kDa heat shock protein, HSP90, binds unfolded proteins to prevent aggregation and is, together with HSC70 (cytosolic HSP70), and HSP40 (cytosolic DnaJ homologue), involved in protein refolding [49]. PA28 serves as a linker between HSP90 and HSC70 and is a necessary cofactor for protein remodeling [50]. Since PA28 can act as a chaperone cofactor, the observed effects on mHTT(Q97) could be mediated by the ability of PA28 to interfere with aggregate formation. Indeed, hippocampal extracts from PA28α overexpressing mice, prevented aggregation of heat sensitive luciferase [31,32]. In these samples activity of PA28α activated proteasomes was not increased and the total amount of damaged proteins was not altered, suggesting that chaperone-like activity is responsible for the observed effects. Overexpression of PA28γ in the striatum of HD mice reduced HD pathology as demonstrated by behavioral tasks [33]. Furthermore, in these mice PA28γ overexpression led to reduced levels of mHTT aggregation, but no changes in mHTT levels were observed. This could again point towards a potential chaperone function of PA28 in obstructing mHTT aggregate formation.

Another known function of PA28αβ is its role in the cell’s protection mechanism against oxidative stress. Rapidly after the induction of oxidative stress by hydrogen peroxide, PA28αβ and PA28γ bind to free 20S subunits [29]. *In vitro* experiments show that PA28αβ activated proteasomes improve the selective degradation of oxidized proteins, but not their native form [29]. In cells, overexpression of PA28α also improves the capability of the proteasome to degrade oxidized proteins [30]. Reducing PA28αβ levels on the other hand, leads to an increase in carbonylated proteins in differentiating embryonic stem cells, without altering proteasome content [51]. HD progression is associated with increased oxidative damage to DNA, proteins and lipids, which contributes to neurodegeneration (reviewed by Kumar and Ratan [52] and Gkekas et al. [53]). mHTT disrupts nuclear integrity and both mHTT protein and expanded CAG RNA impair DNA repair mechanisms [54–58]. In combination with the increased oxidative damage to DNA, this contributes to neuronal pathology. Furthermore, HTT itself can be oxidized, which leads to stabilization of mHTT oligomers and accelerates aggregation, by facilitating aggregate interactions [59–61]. By the dissociation of PA28αβ from the 20S core, the proteasome is less adapted to degrade oxidized proteins, which could accelerate oxidative stress and subsequent pathology in HD.

The question remains whether dissociation of PA28αβ activators from the 20S core in the cortex, striatum and hippocampus of HD mice is a cause or consequence of increased mHTT aggregation. During normal homeostasis, proteasome complex formation is a dynamic process, which is altered upon several stimuli, including inhibition of catalytic activity, pro-inflammatory stimuli and competition between the different PAs [51,62–64]. The pool of free PA28αβ heptamers, which are not bound to 20S core subunits, suggests that the cell has free PA28 activators that can be used to quickly increase PA28αβ-mediated proteasome activation. This underlines the ability of cells to dynamically regulate the number of PA28αβ activated proteasomes. Upon hydrogen peroxide treatment, levels of PA28αβ activated proteasomes increase within the first hour prior to synthesis of new PA28αβ complexes [29]. Moreover, PA28γ activated proteasomes increase within hours after proteasome inhibition, without an increase in PA28γ transcription [64]. Although the exact signaling pathways remain elusive, phosphorylation of PA28αβ is found to be involved in its binding to the 20S core subunit [65]. Several studies, however, show that kinases are dysregulated in HD (reviewed by Bowles and Jones [66]). This may imply that during HD, general dysregulation of the signaling pathways responsible for proteasome conformational changes may lead to the disassembly of PA28αβ activated proteasomes, after which normal functioning is impaired. However, PA28αβ-20S disassembly may also be a specific response to the increase in mHTT aggregates in affected cells. Since PA28αβ is involved in the clearance of aggregates though its chaperone like function, the increased need for free-PA28αβ may cause the disassembly from the 20S core. Based on this hypothesis, the PA28αβ-20S disassembly observed in mouse brain would not lead to increased aggregation but would rather be a coping mechanism to deal with the aggregates already present in the cell. Indeed, aggregates are already present at the disease stages at which we observe the change in PA28αβ activated proteasomes [67–69].

## Supporting information

S1 Checklist (PDF)

S1 Fig Activity Based Probe labeling in mouse cells and brain tissue. Mouse whole brain lysate and 20S, 20S+PA28αβ, 26S+PA28αβ and 26S were pre-incubated with ABP and separated on native PAGE to visualize the different active proteasome complexes. In mouse brain tissue the upper fluorescent signal represents the 30S proteasomes (double 19S capped 20S). Just below the 30S band run the 26S (20S+19S) and the hybrid (20S+19S+PA28) proteasomes. The lower fluorescence band represents PA28 capped 20S. It needs to be noted that PA28αβ competes with the 19S cap for binding to the 20S core, resulting in loss of 26S proteasomes when 26S is combined with PA28αβ. After the addition of epoxomicin to the mouse brain lysate ABP labeling decreases, whereas α7 labeling remains.
(TIF)

S2 Fig Increased clearance of pathogenic polyQ peptides by PA28αβ after proteasome inhibition. PA28αβ, PA28α or PA28β was overexpressed in HEK293 cells. During the last 16 hours the cells were incubated with proteasome inhibitor epoxomicin. Data are normalized to pcDNA transfected cells and shown as mean ± SEM (n = 2–3). One-way ANOVA with Dunnet’s multiple testing; soluble mHTT soluble PA28αβ p = 0.5945; PA28α p = 0.9993; PA28β p = 0.8558.; DNAJB6 p = 0.8882; insoluble mHTT PA28αβ p = 0.1452; PA28α p = 0.2714; PA28β p = 0.7467; DNAJB6 p = 0.1119.
(TIF)

S3 Fig Different proteasome complex composition in HEK293 and ST*Hdh* cells. Native PAGE showing different proteasome complexes in HEK293 and STHdh cells. Immunoblots for α7, RPT1 and PA28α show core complexes, 19S activated proteasomes and PA28αβ activated proteasomes, respectively.
(TIF)

S1 Raw images (PDF)

S1 File (ZIP)
