# Supplementary figures and images for: Reduction in PA28αβ activation in HD mouse brain correlates to increased mHTT aggregation in cell models
Source: PLoS One. 2022 Dec 27;17(12):e0278130. doi: 10.1371/journal.pone.0278130 (PMC9794069; doi:10.1371/journal.pone.0278130)

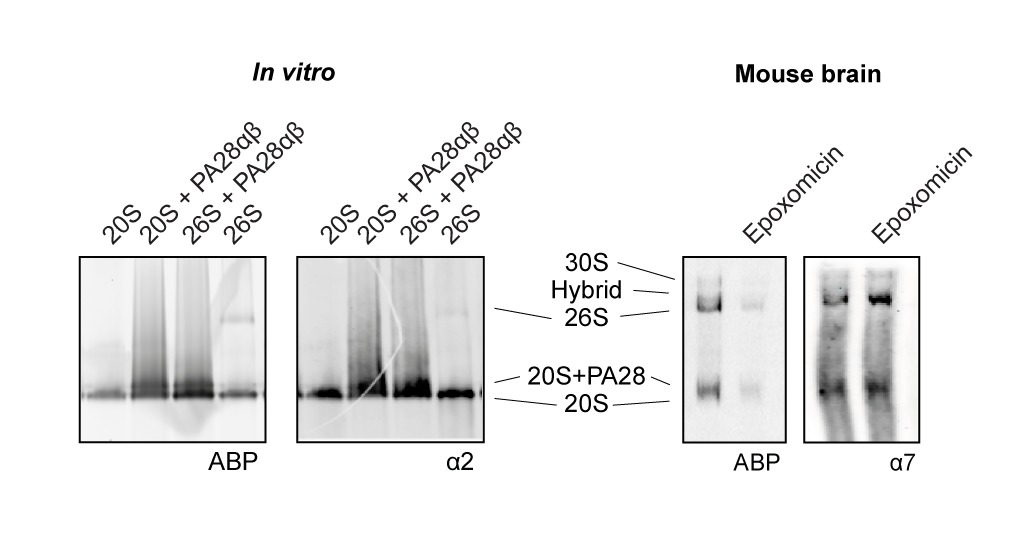

Supplement: S1 Fig — Mouse whole brain lysate and 20S, 20S+PA28αβ, 26S+PA28αβ and 26S were pre-incubated with ABP and separated on native PAGE to visualize the different active proteasome complexes. In mouse brain tissue the upper fluorescent signal represents the 30S proteasomes (double 19S capped 20S). Just below the 30S band run the 26S (20S+19S) and the hybrid (20S+19S+PA28) proteasomes. The lower fluorescence band represents PA28 capped 20S. It needs to be noted that PA28αβ competes with the 19S cap for binding to the 20S core, resulting in loss of 26S proteasomes when 26S is combined with PA28αβ. After the addition of epoxomicin to the mouse brain lysate ABP labeling decreases, whereas α7 labeling remains. (TIF) [file pone.0278130.s002.tif]

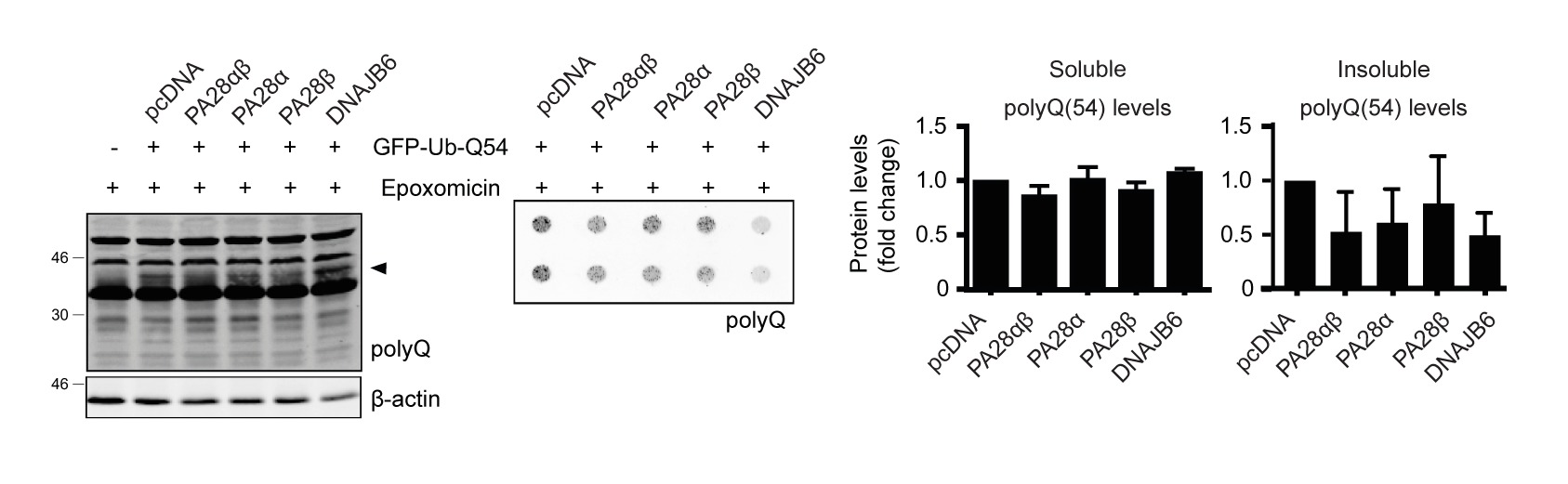

Supplement: S2 Fig — PA28αβ, PA28α or PA28β was overexpressed in HEK293 cells. During the last 16 hours the cells were incubated with proteasome inhibitor epoxomicin. Data are normalized to pcDNA transfected cells and shown as mean ± SEM (n = 2–3). One-way ANOVA with Dunnet’s multiple testing; soluble mHTT soluble PA28αβ p = 0.5945; PA28α p = 0.9993; PA28β p = 0.8558.; DNAJB6 p = 0.8882; insoluble mHTT PA28αβ p = 0.1452; PA28α p = 0.2714; PA28β p = 0.7467; DNAJB6 p = 0.1119. (TIF) [file pone.0278130.s003.tif]

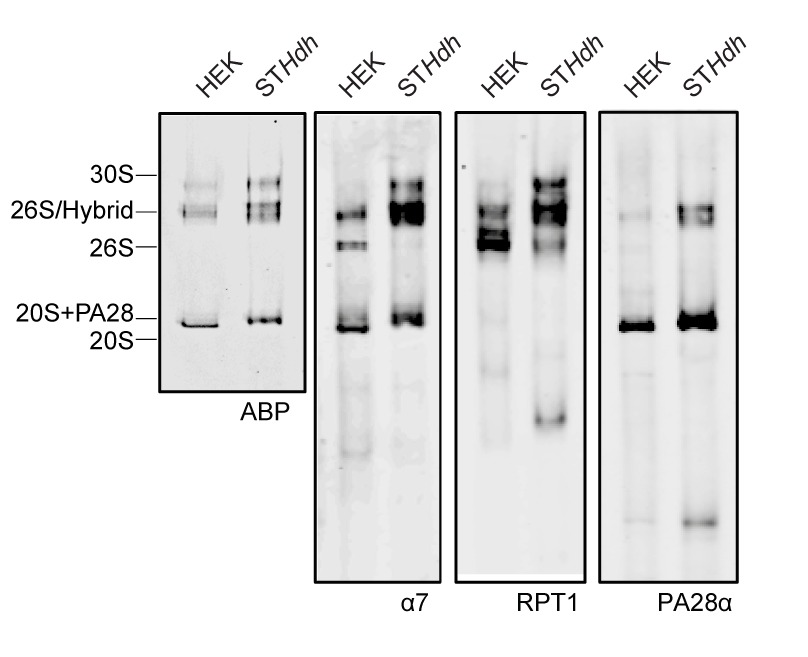

Supplement: S3 Fig — Native PAGE showing different proteasome complexes in HEK293 and STHdh cells. Immunoblots for α7, RPT1 and PA28α show core complexes, 19S activated proteasomes and PA28αβ activated proteasomes, respectively. (TIF) [file pone.0278130.s004.tif]

2C

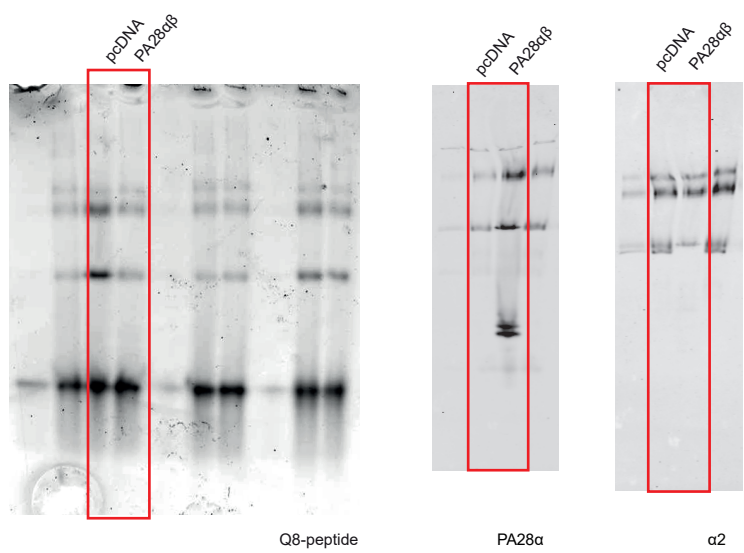

2D

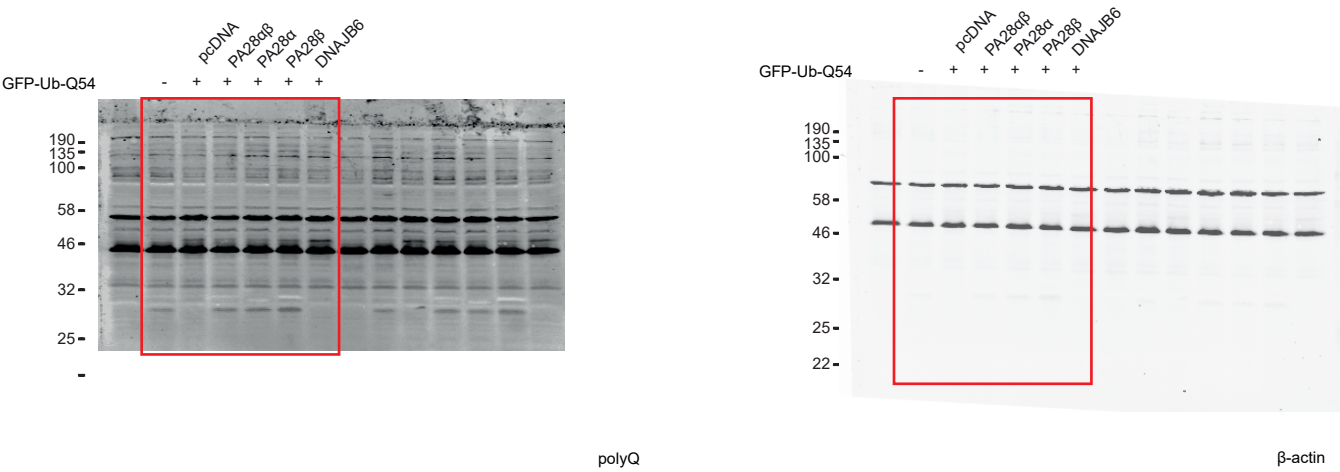

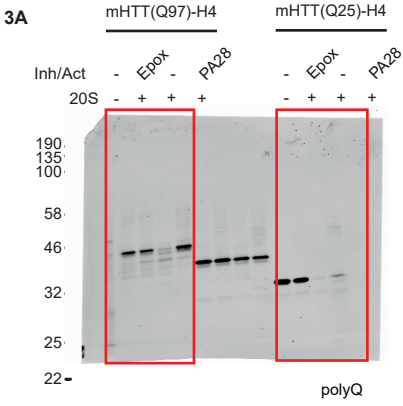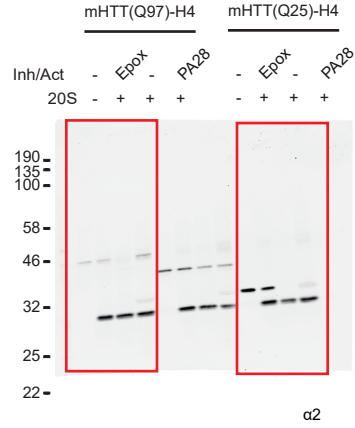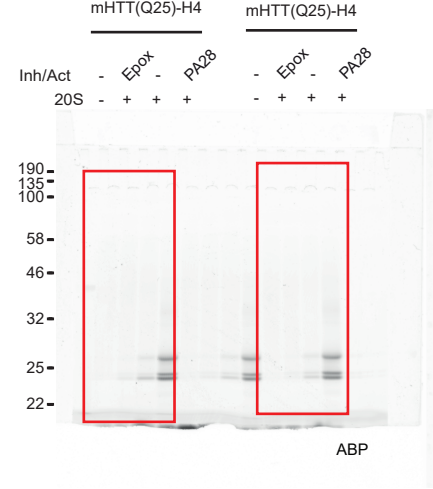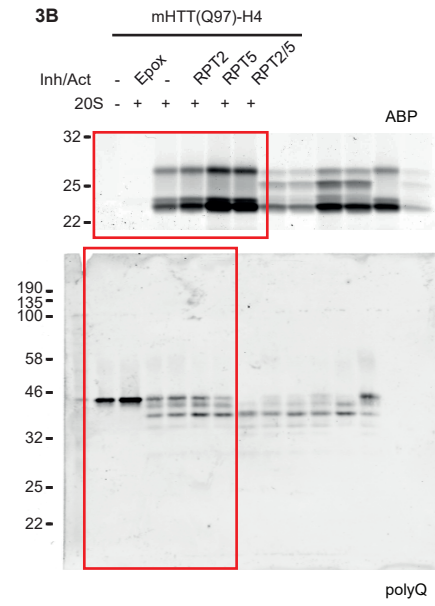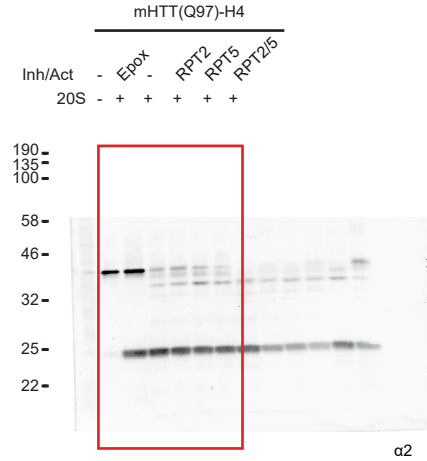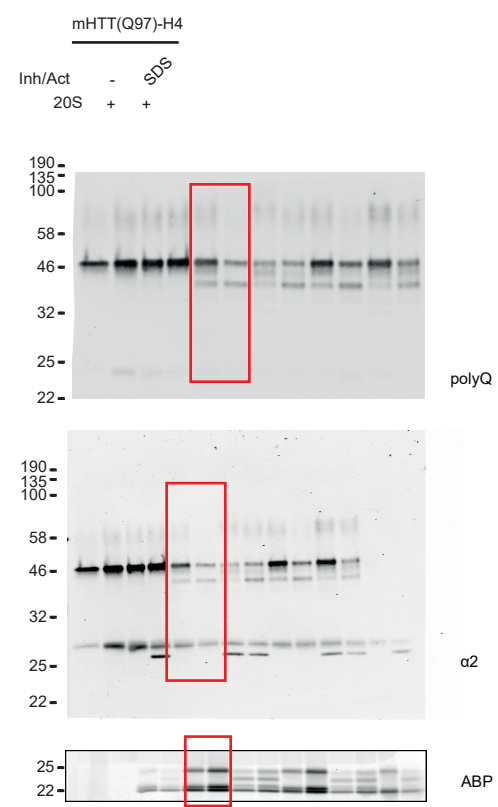

4A

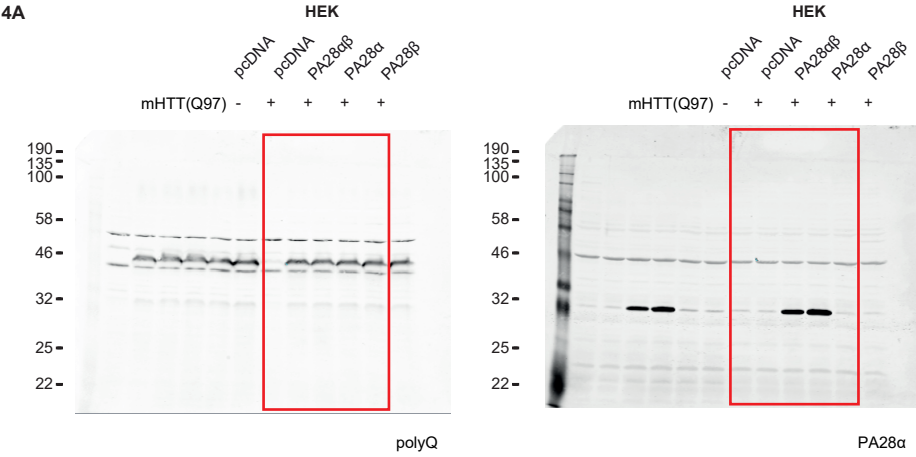

4B

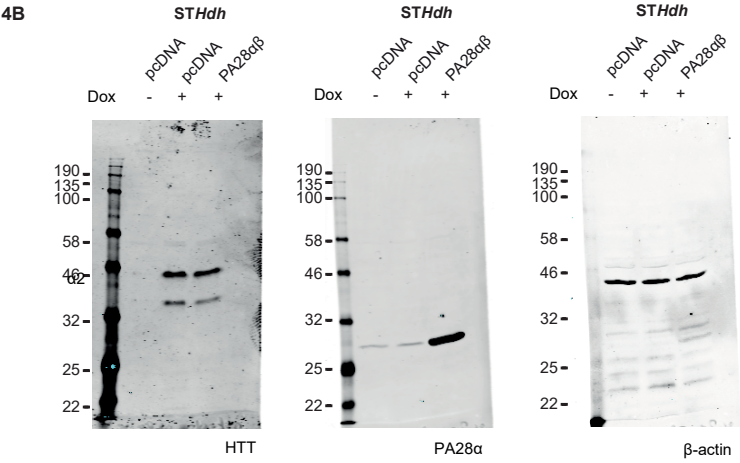

5A

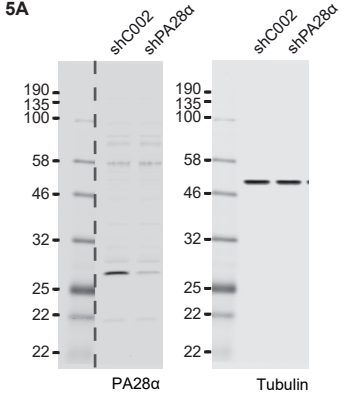

5C/F

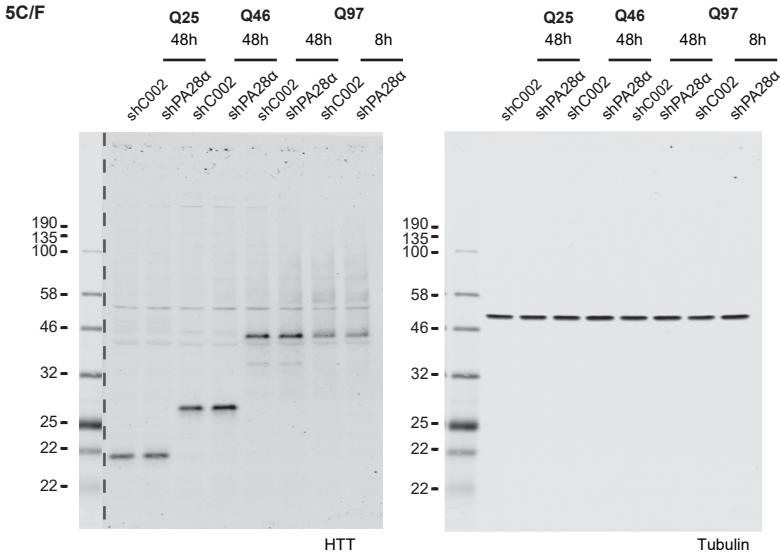

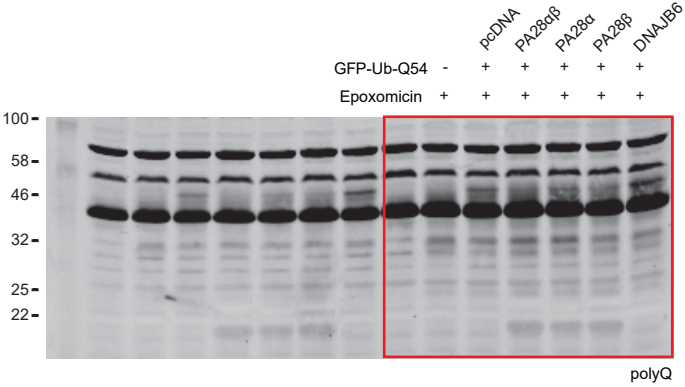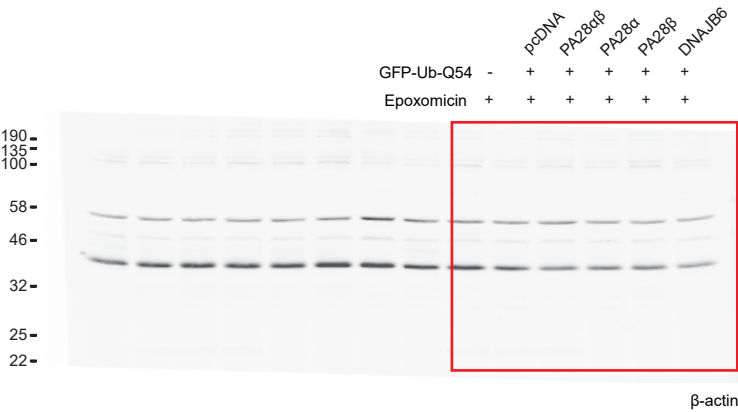

Supplement: S1 Raw images — (PDF) [file pone.0278130.s005.pdf]

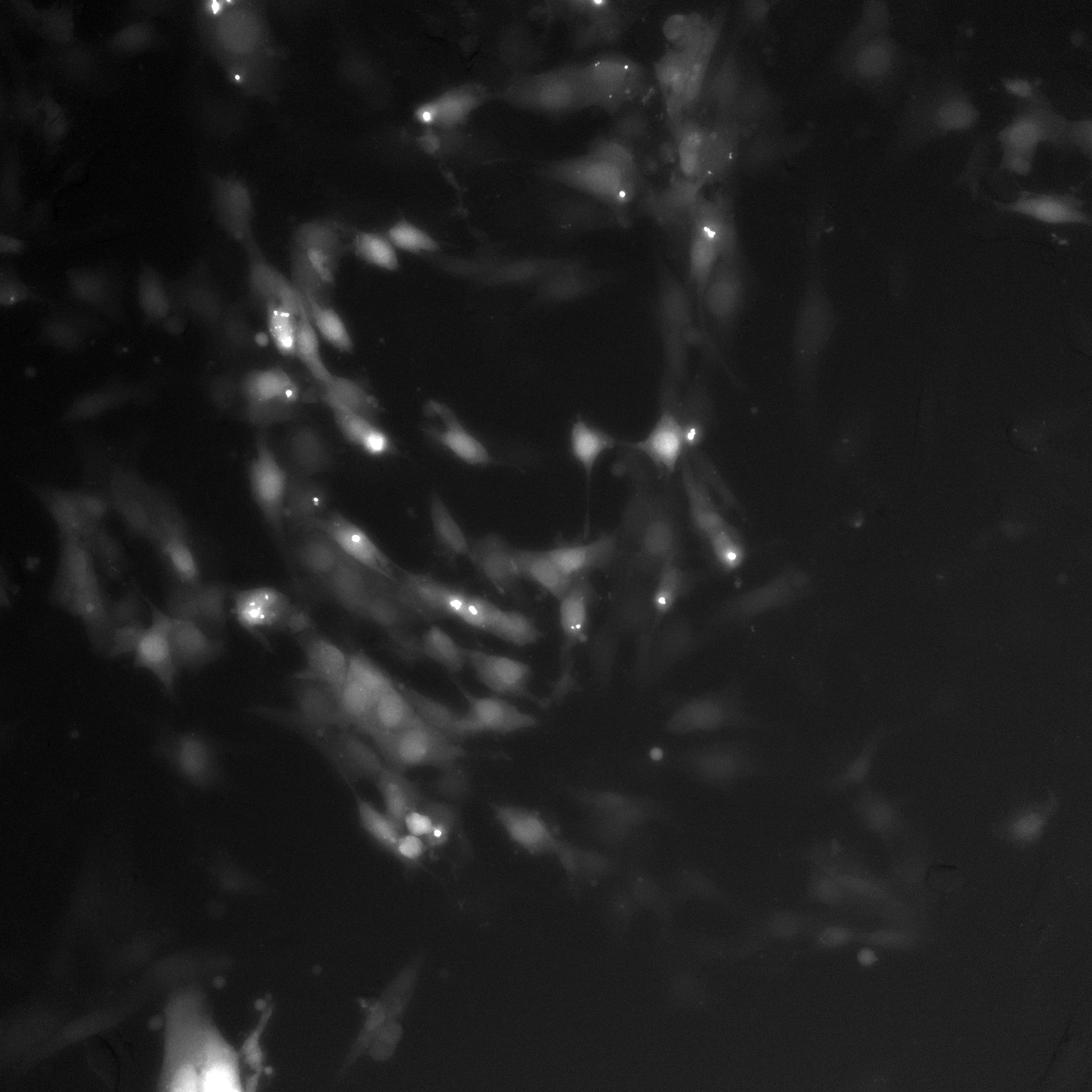

Supplement: S1 File — (ZIP) [file pone.0278130.s006.zip › Supporting Information_Matlab/20210428_ST_72_n3_dnajb6_E04_aggr_14.tif]

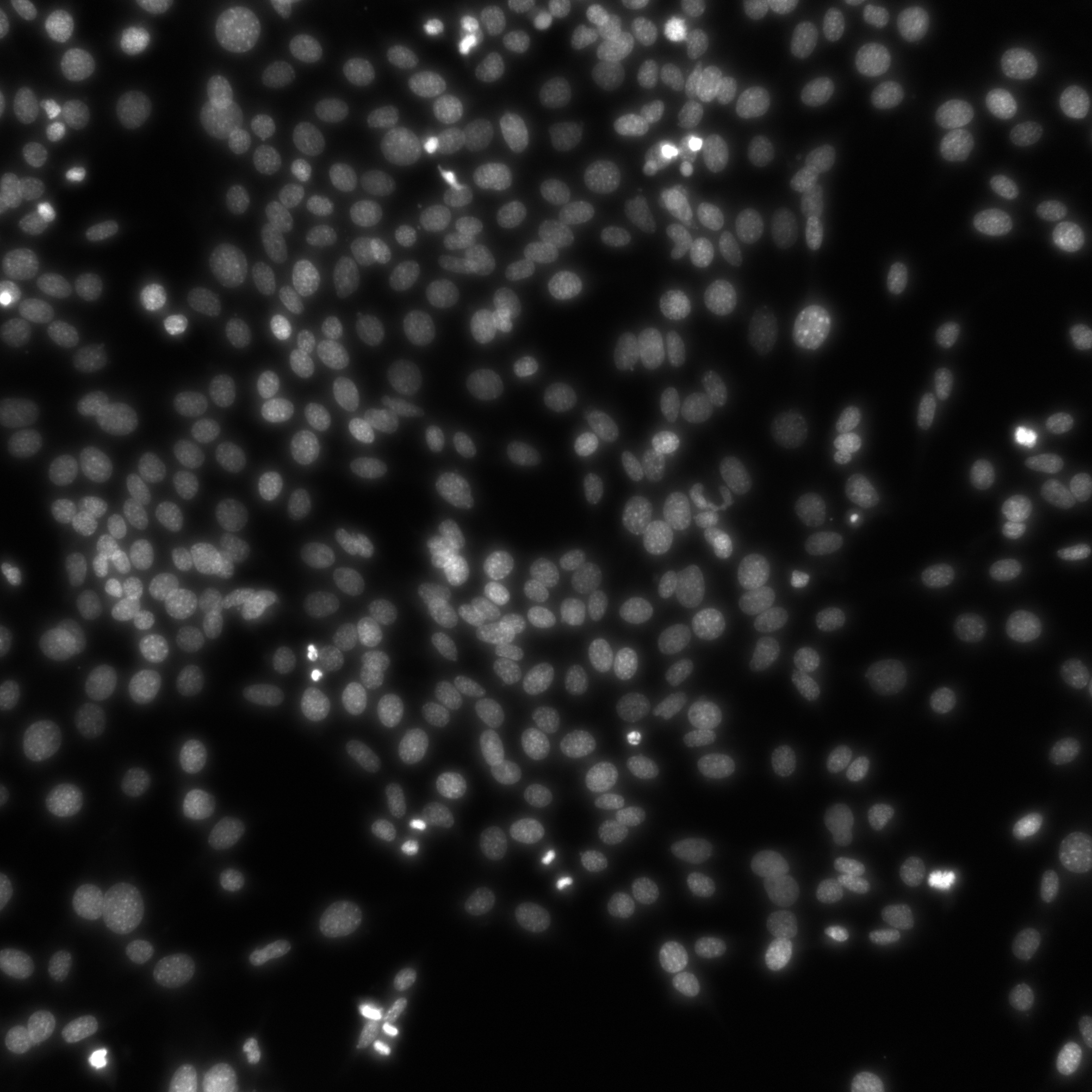

Supplement: S1 File — (ZIP) [file pone.0278130.s006.zip › Supporting Information_Matlab/20210428_ST_72_n3_dnajb6_E04_nuclei_14.tif]

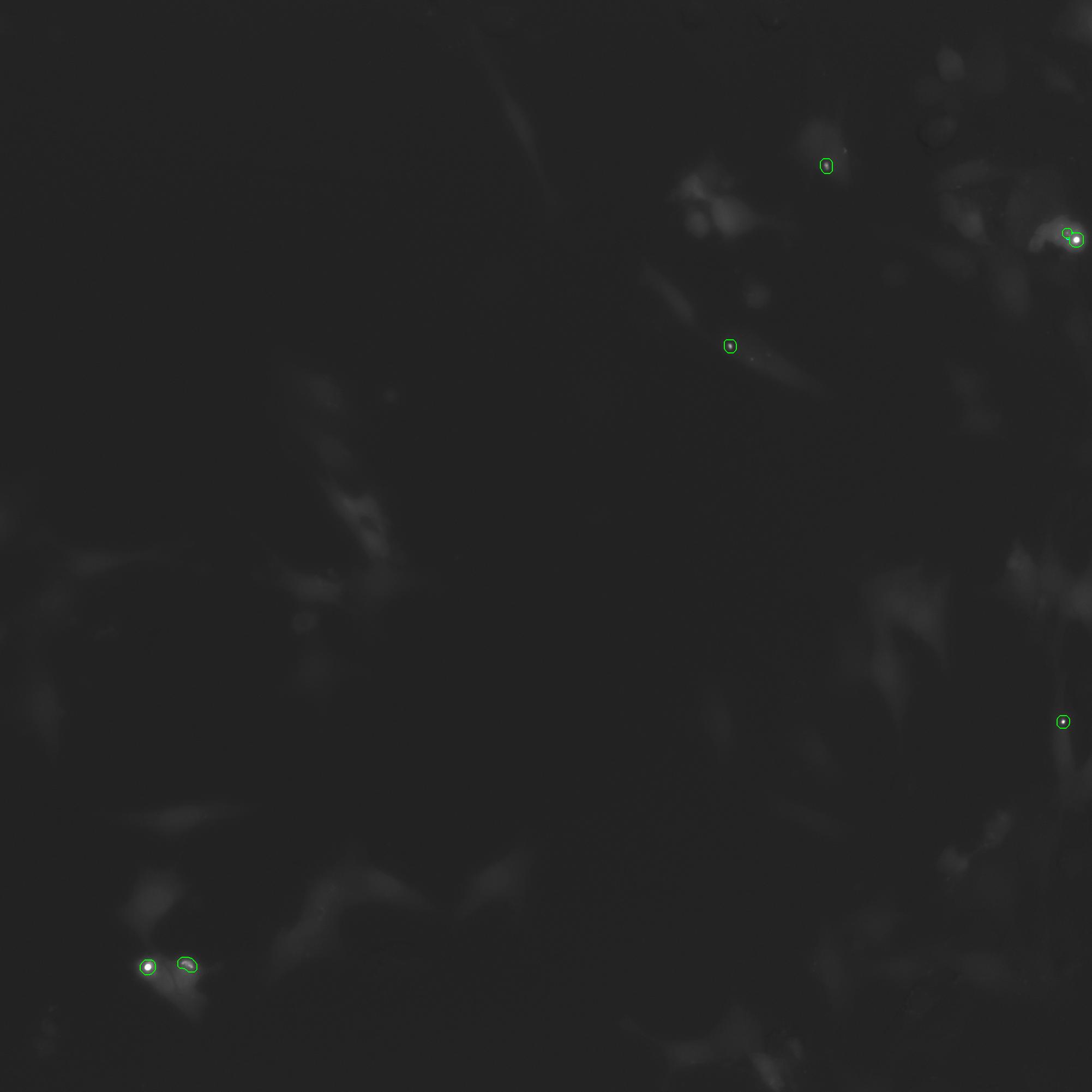

Supplement: S1 File — (ZIP) [file pone.0278130.s006.zip › Supporting Information_Matlab/ExampleData/ScreenWells/AnalyseImages/E04_001_aggr.jpg]

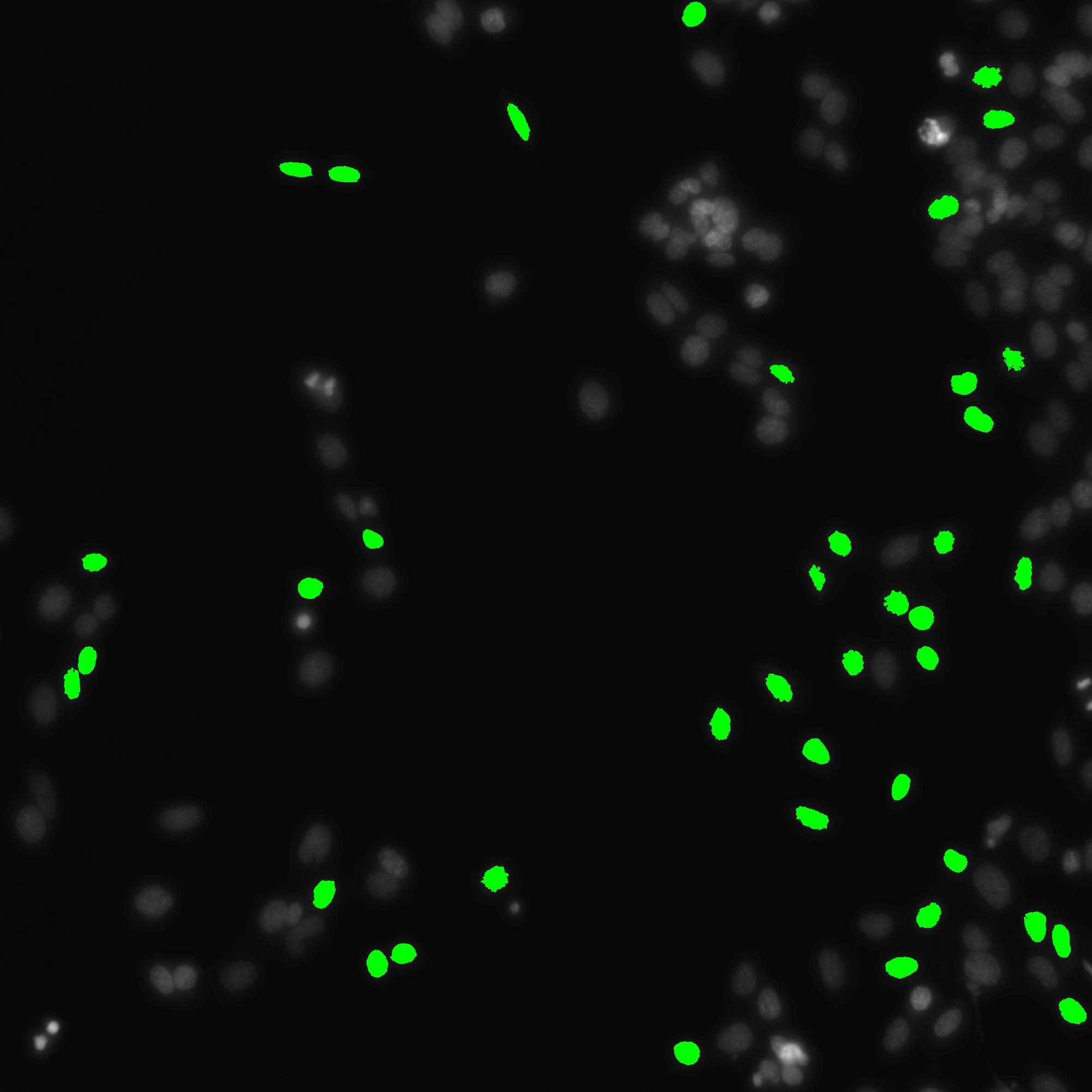

Supplement: S1 File — (ZIP) [file pone.0278130.s006.zip › Supporting Information_Matlab/ExampleData/ScreenWells/AnalyseImages/E04_001_singlenucl.jpg]

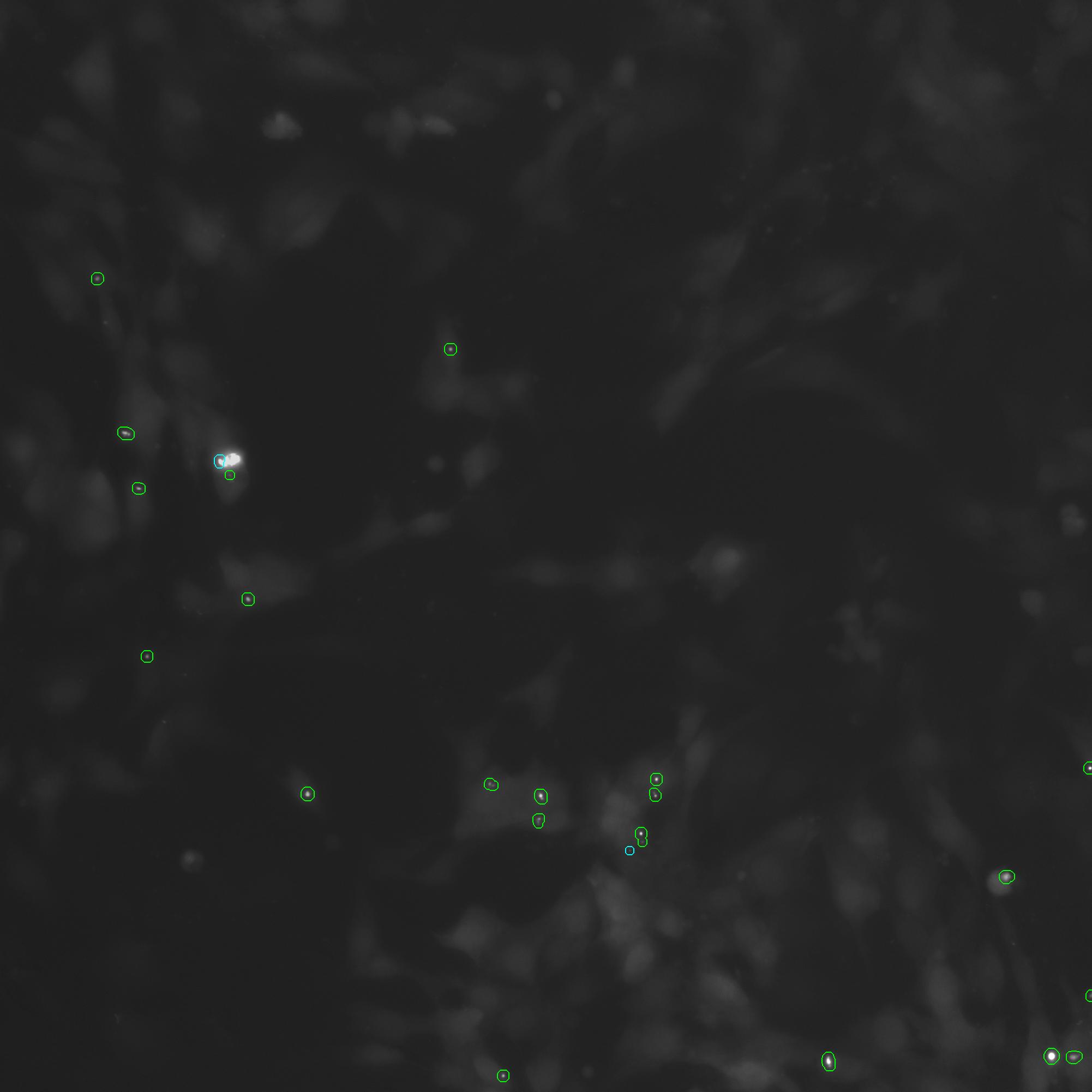

Supplement: S1 File — (ZIP) [file pone.0278130.s006.zip › Supporting Information_Matlab/ExampleData/ScreenWells/AnalyseImages/E04_002_aggr.jpg]

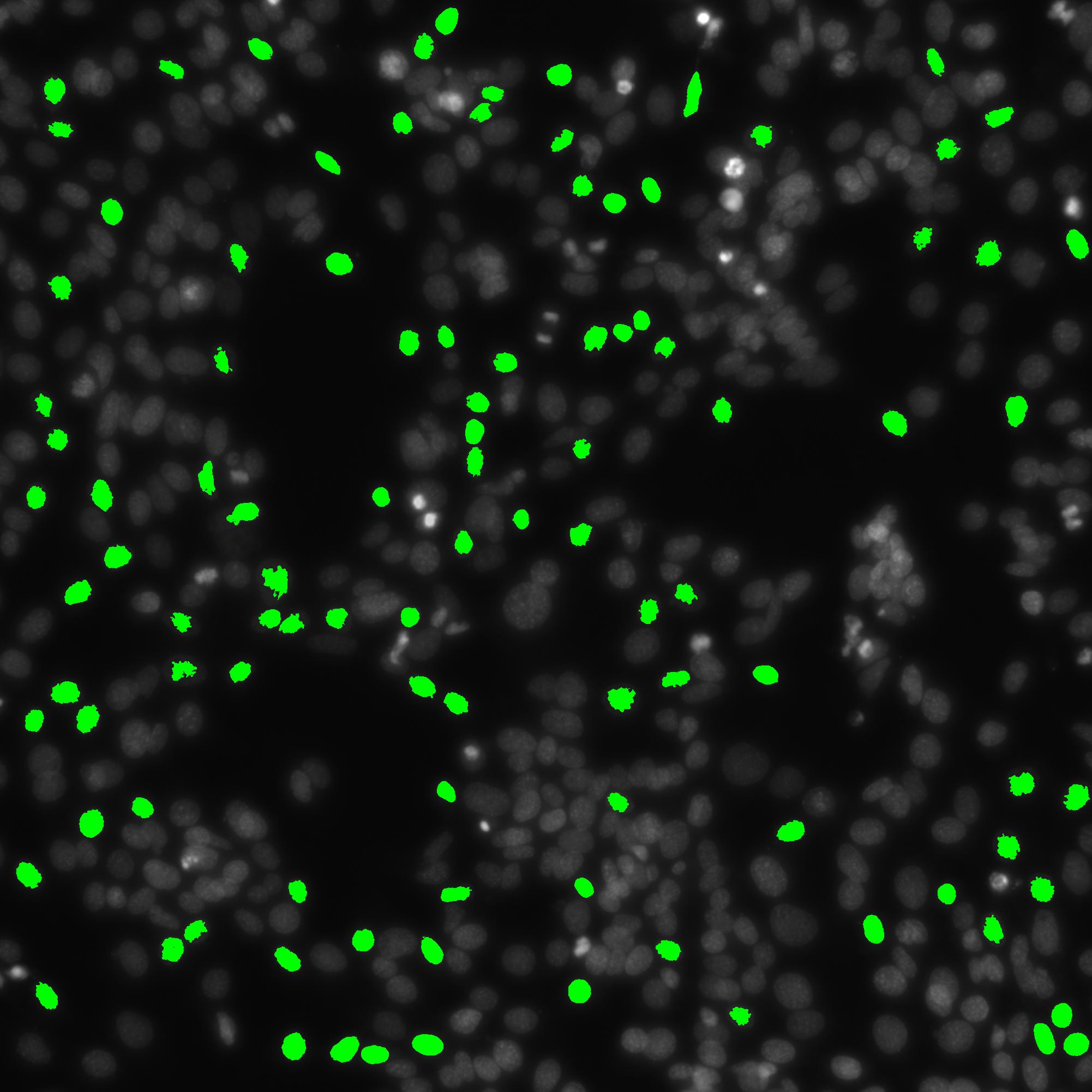

Supplement: S1 File — (ZIP) [file pone.0278130.s006.zip › Supporting Information_Matlab/ExampleData/ScreenWells/AnalyseImages/E04_002_singlenucl.jpg]

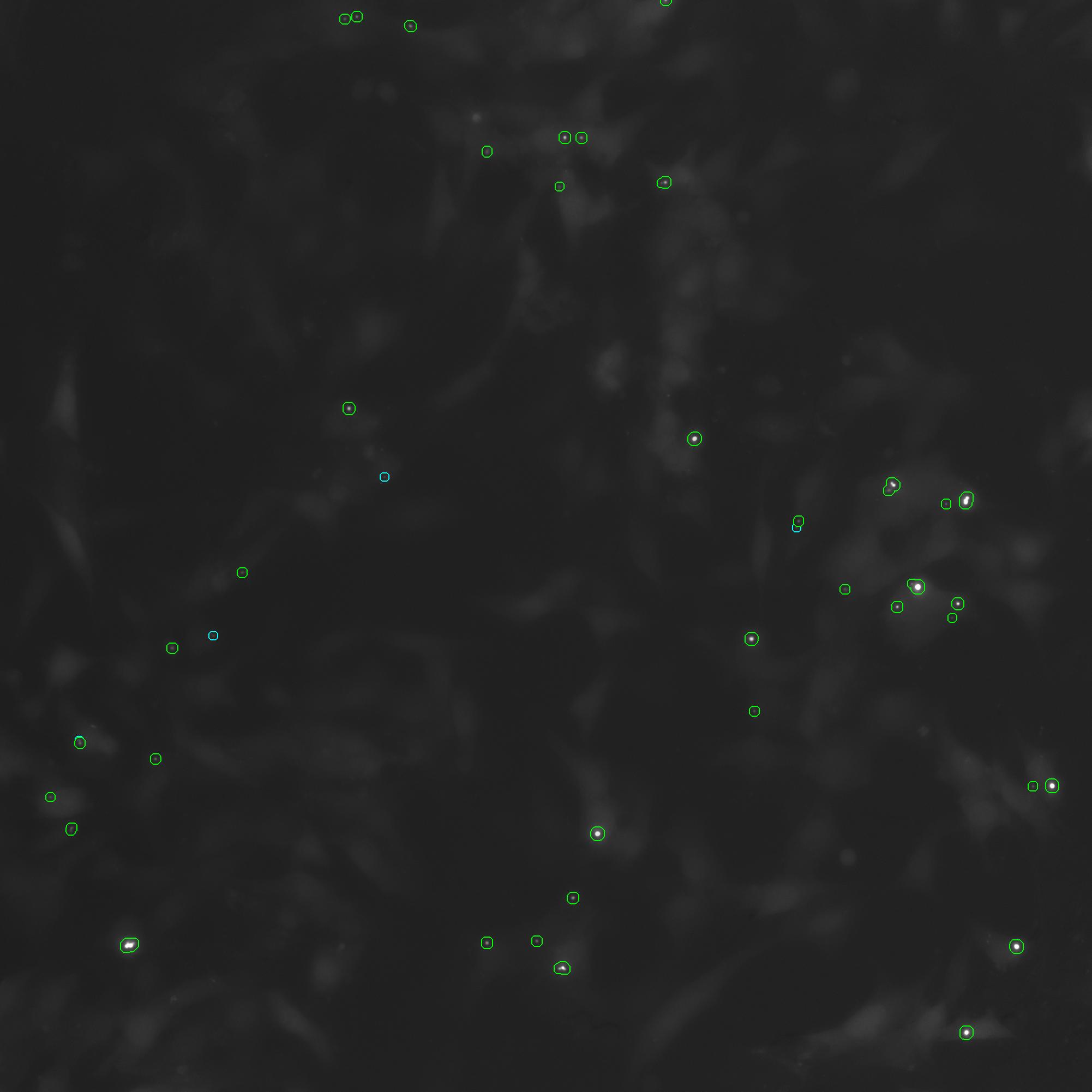

Supplement: S1 File — (ZIP) [file pone.0278130.s006.zip › Supporting Information_Matlab/ExampleData/ScreenWells/AnalyseImages/E04_003_aggr.jpg]

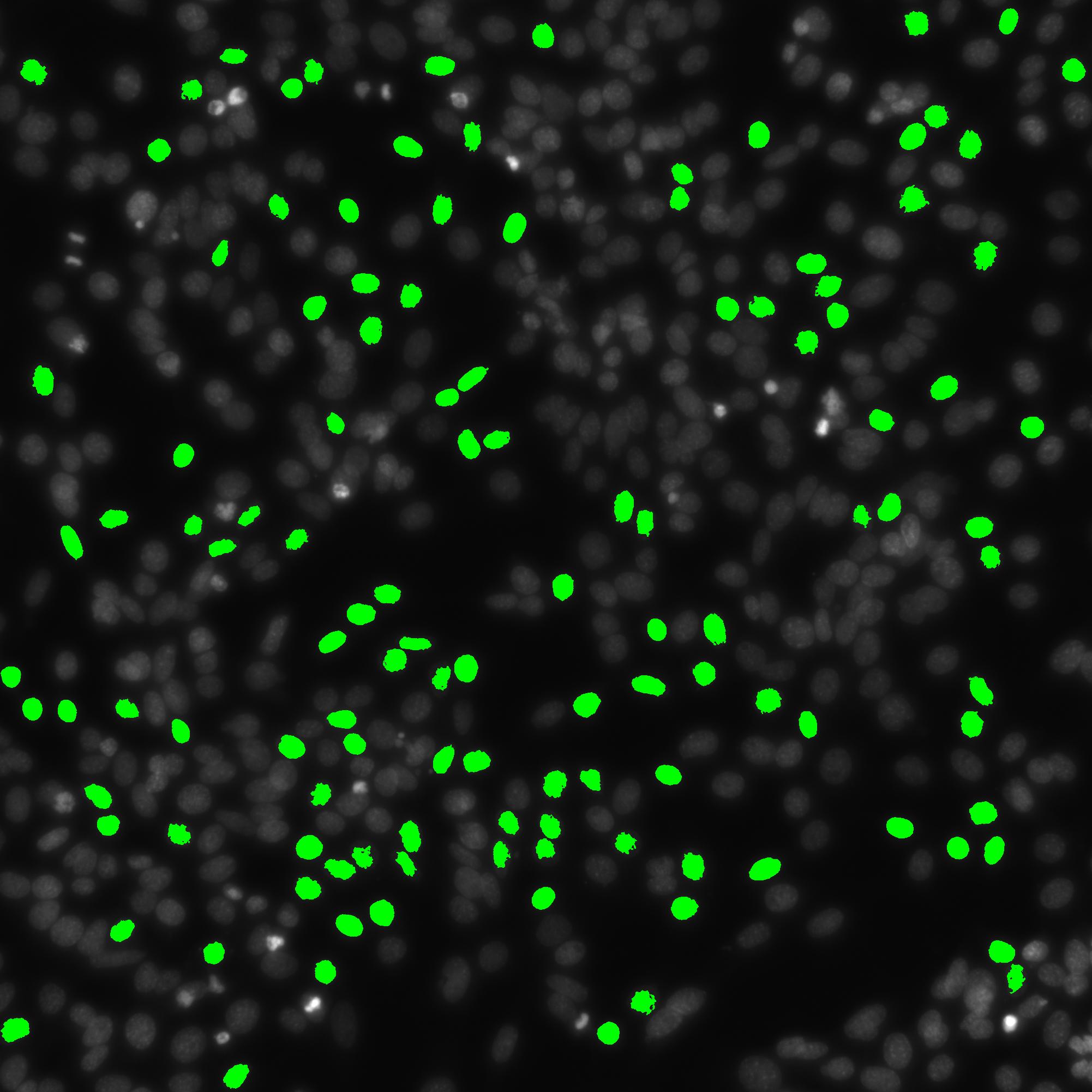

Supplement: S1 File — (ZIP) [file pone.0278130.s006.zip › Supporting Information_Matlab/ExampleData/ScreenWells/AnalyseImages/E04_003_singlenucl.jpg]

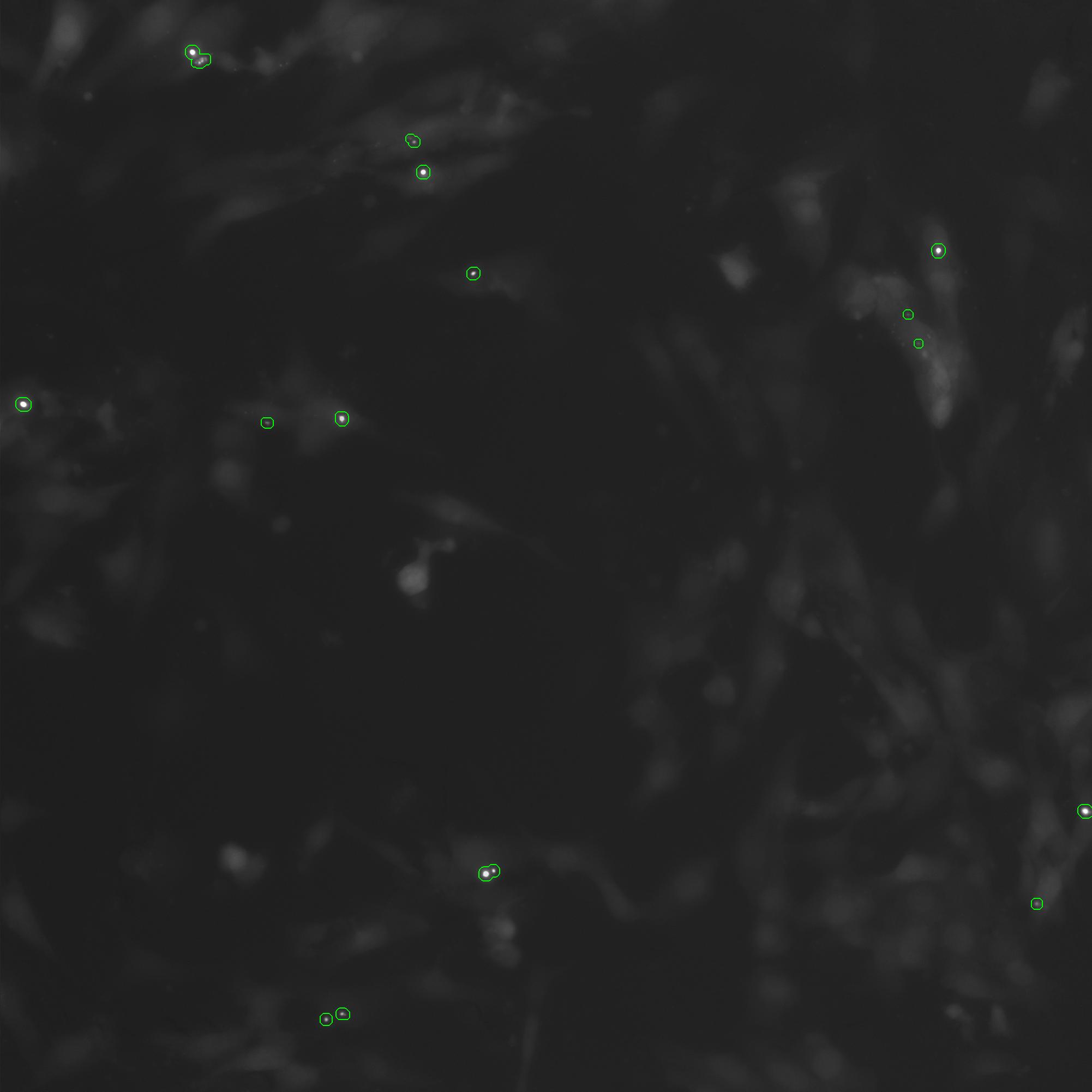

Supplement: S1 File — (ZIP) [file pone.0278130.s006.zip › Supporting Information_Matlab/ExampleData/ScreenWells/AnalyseImages/E04_004_aggr.jpg]

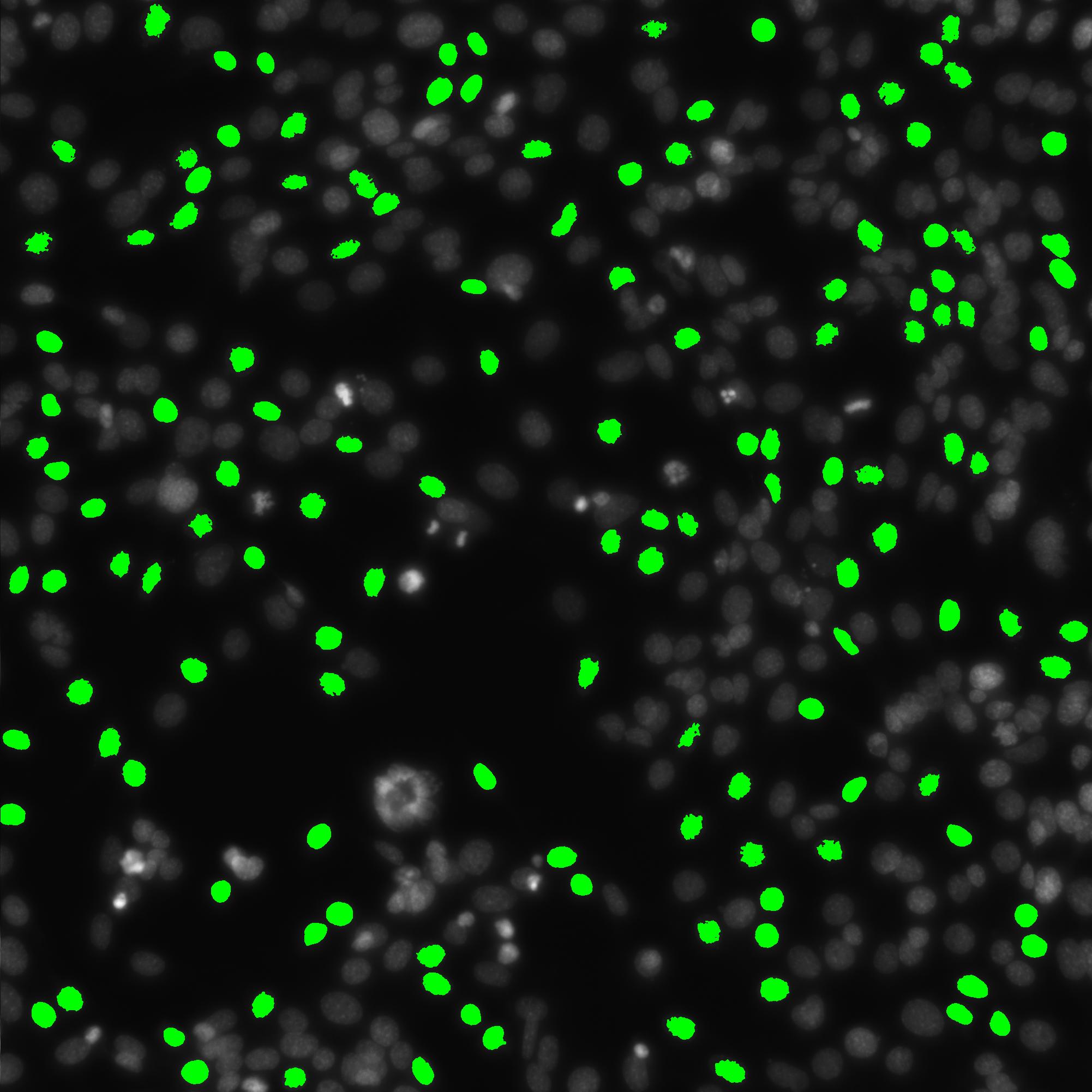

Supplement: S1 File — (ZIP) [file pone.0278130.s006.zip › Supporting Information_Matlab/ExampleData/ScreenWells/AnalyseImages/E04_004_singlenucl.jpg]

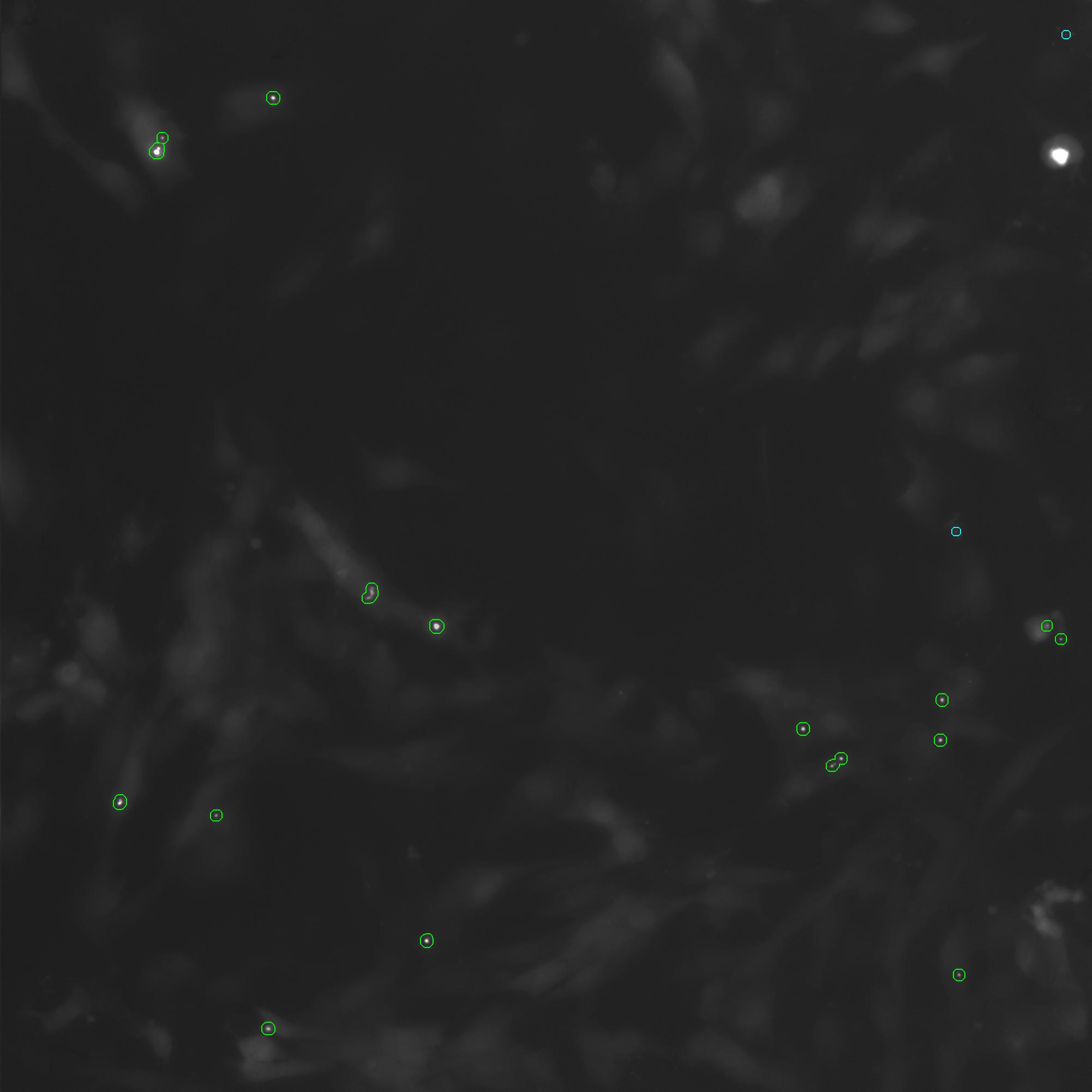

Supplement: S1 File — (ZIP) [file pone.0278130.s006.zip › Supporting Information_Matlab/ExampleData/ScreenWells/AnalyseImages/E04_005_aggr.jpg]

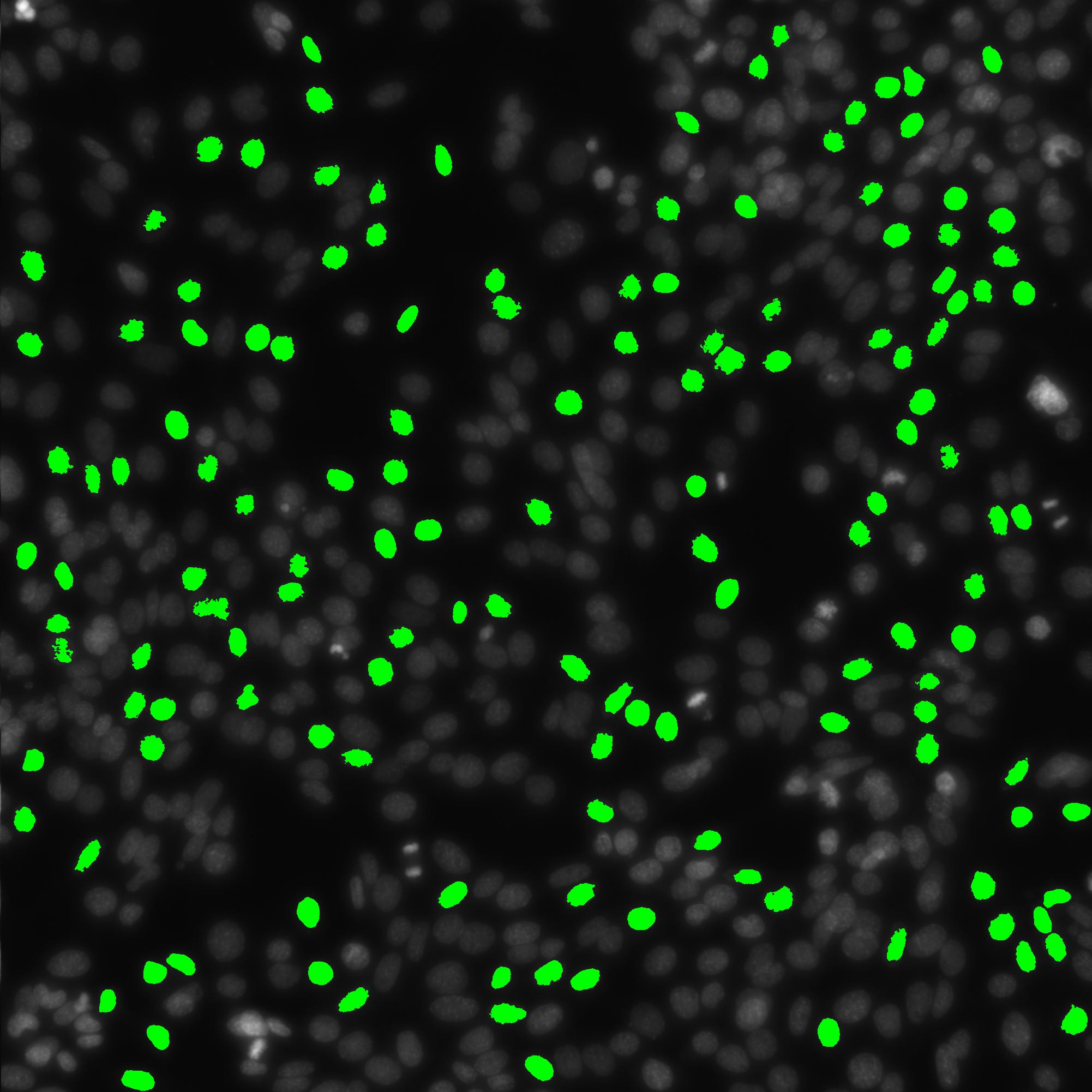

Supplement: S1 File — (ZIP) [file pone.0278130.s006.zip › Supporting Information_Matlab/ExampleData/ScreenWells/AnalyseImages/E04_005_singlenucl.jpg]

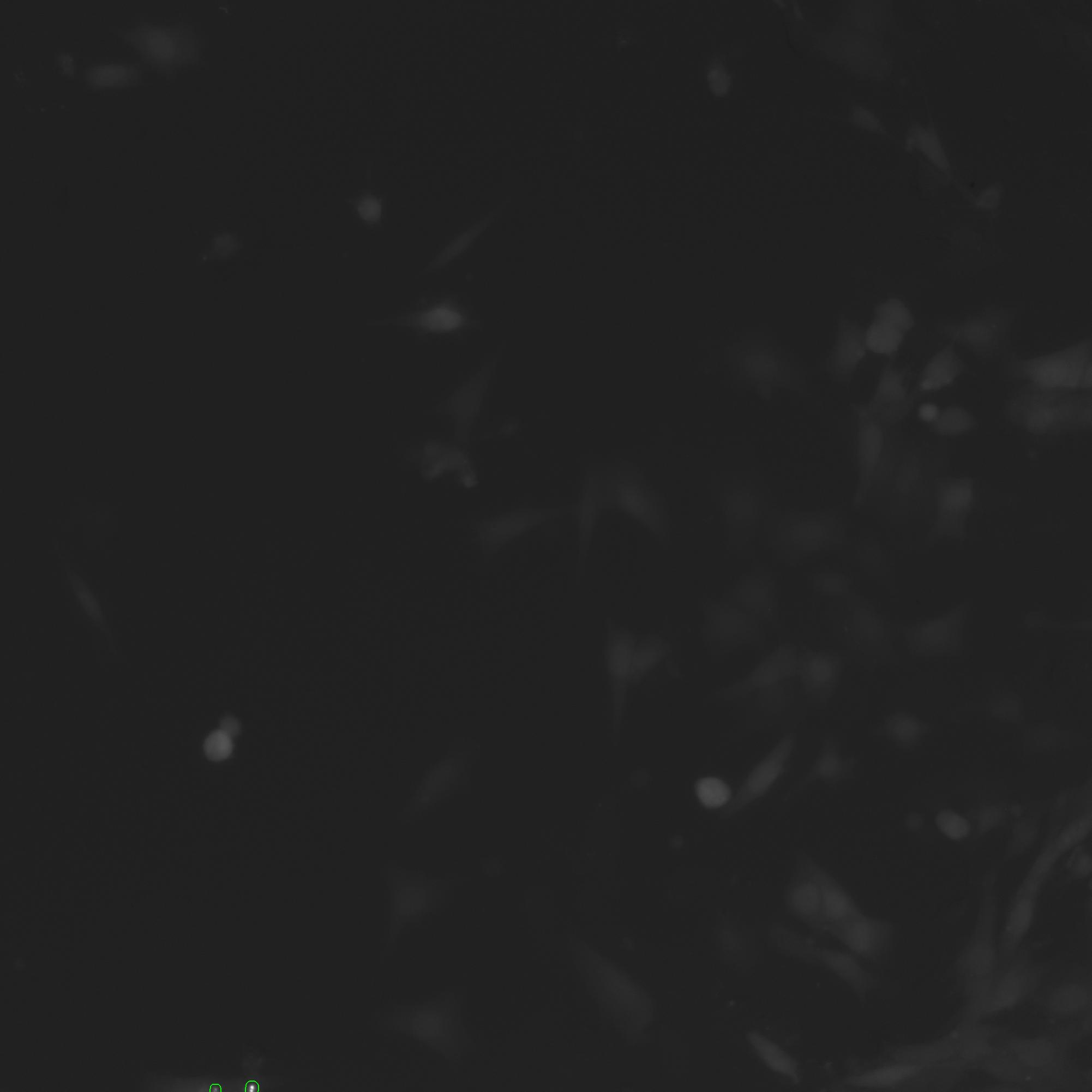

Supplement: S1 File — (ZIP) [file pone.0278130.s006.zip › Supporting Information_Matlab/ExampleData/ScreenWells/AnalyseImages/E04_006_aggr.jpg]

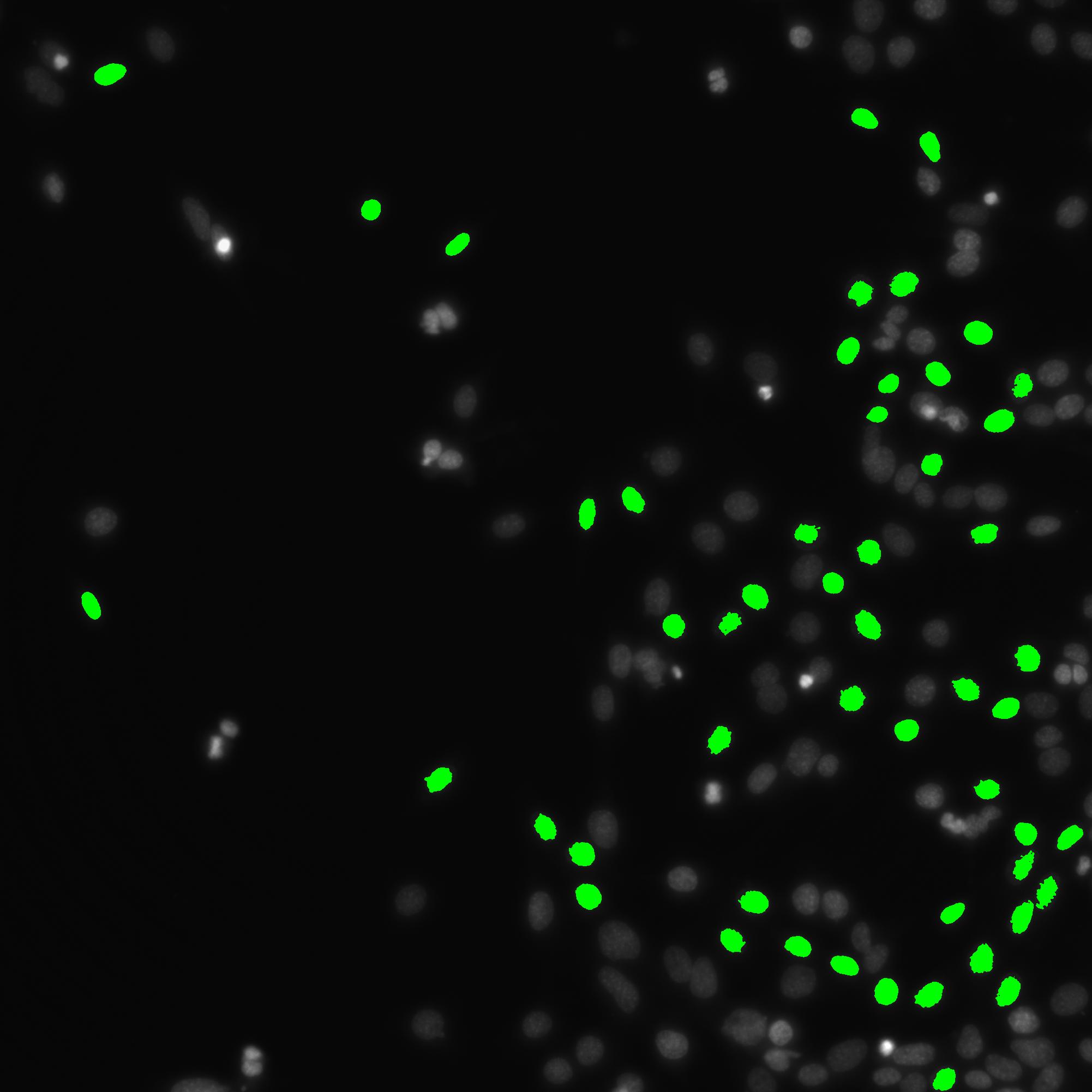

Supplement: S1 File — (ZIP) [file pone.0278130.s006.zip › Supporting Information_Matlab/ExampleData/ScreenWells/AnalyseImages/E04_006_singlenucl.jpg]

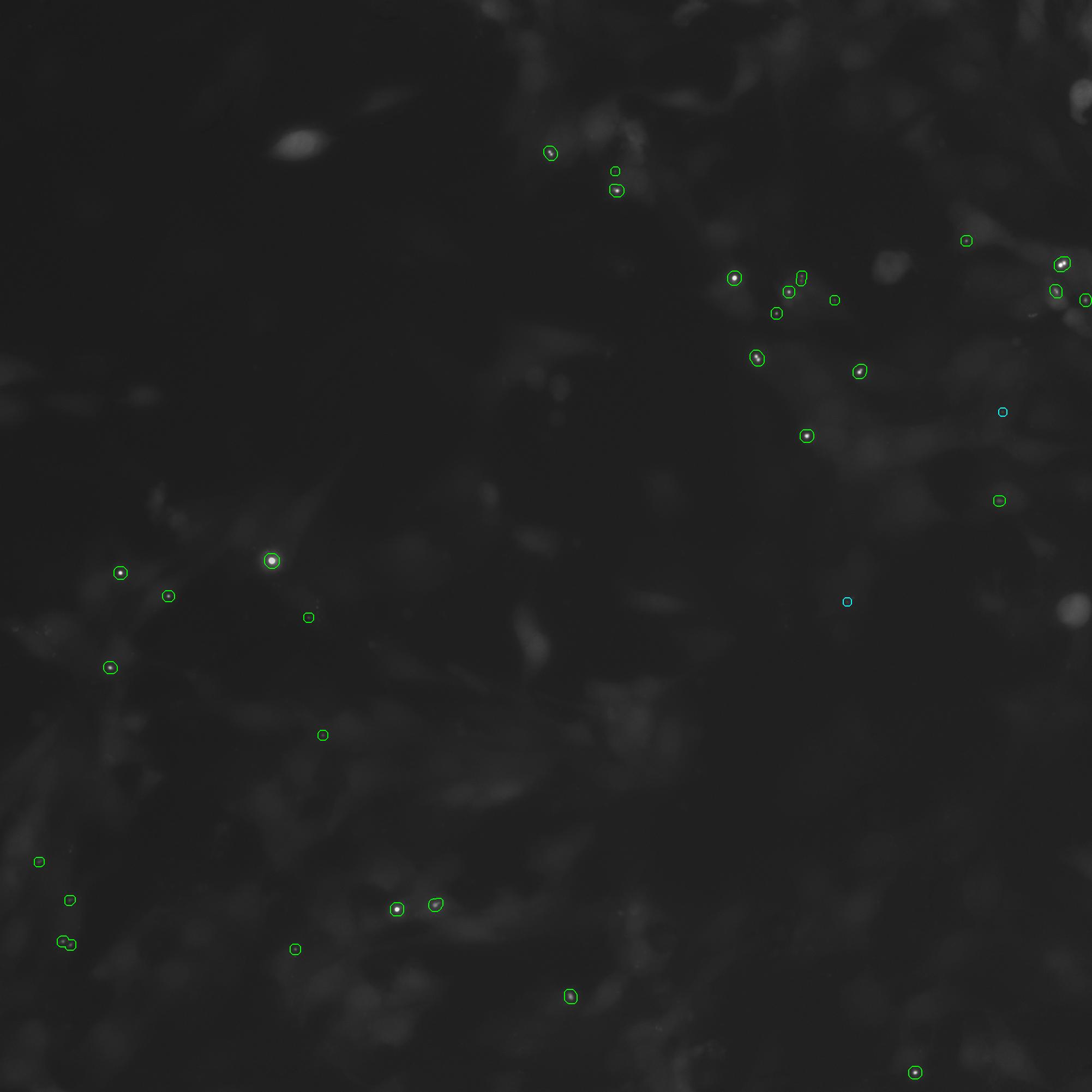

Supplement: S1 File — (ZIP) [file pone.0278130.s006.zip › Supporting Information_Matlab/ExampleData/ScreenWells/AnalyseImages/E04_007_aggr.jpg]

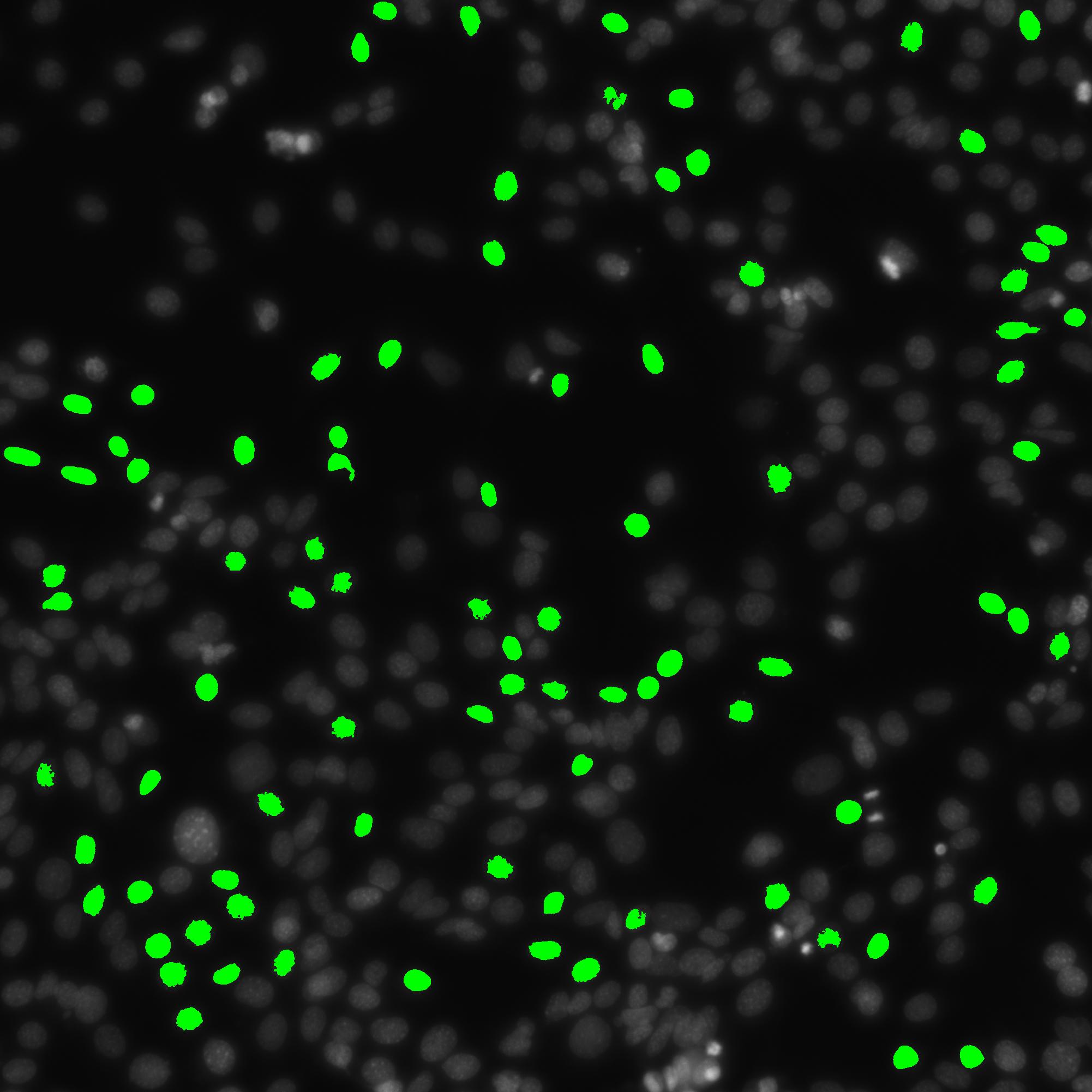

Supplement: S1 File — (ZIP) [file pone.0278130.s006.zip › Supporting Information_Matlab/ExampleData/ScreenWells/AnalyseImages/E04_007_singlenucl.jpg]

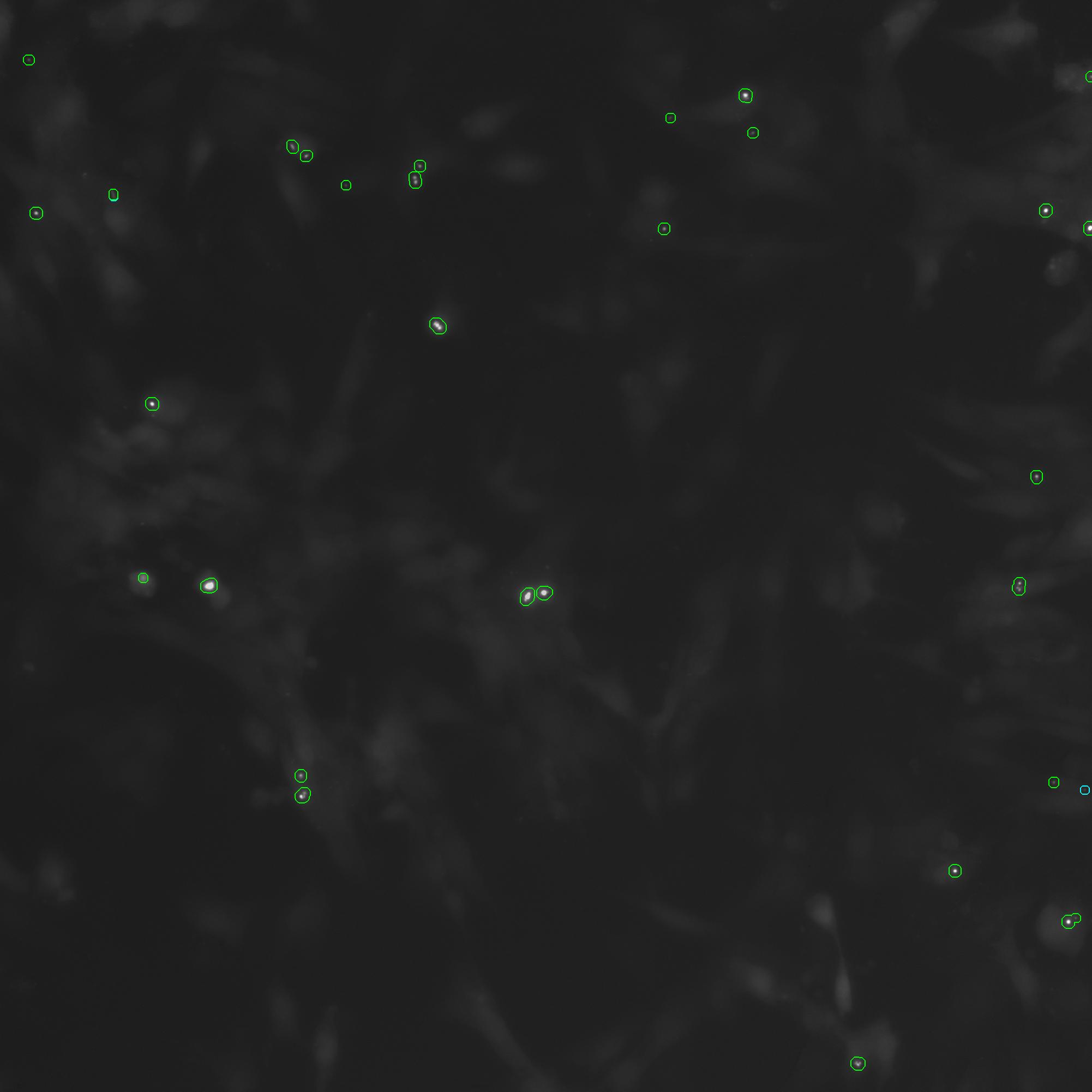

Supplement: S1 File — (ZIP) [file pone.0278130.s006.zip › Supporting Information_Matlab/ExampleData/ScreenWells/AnalyseImages/E04_008_aggr.jpg]

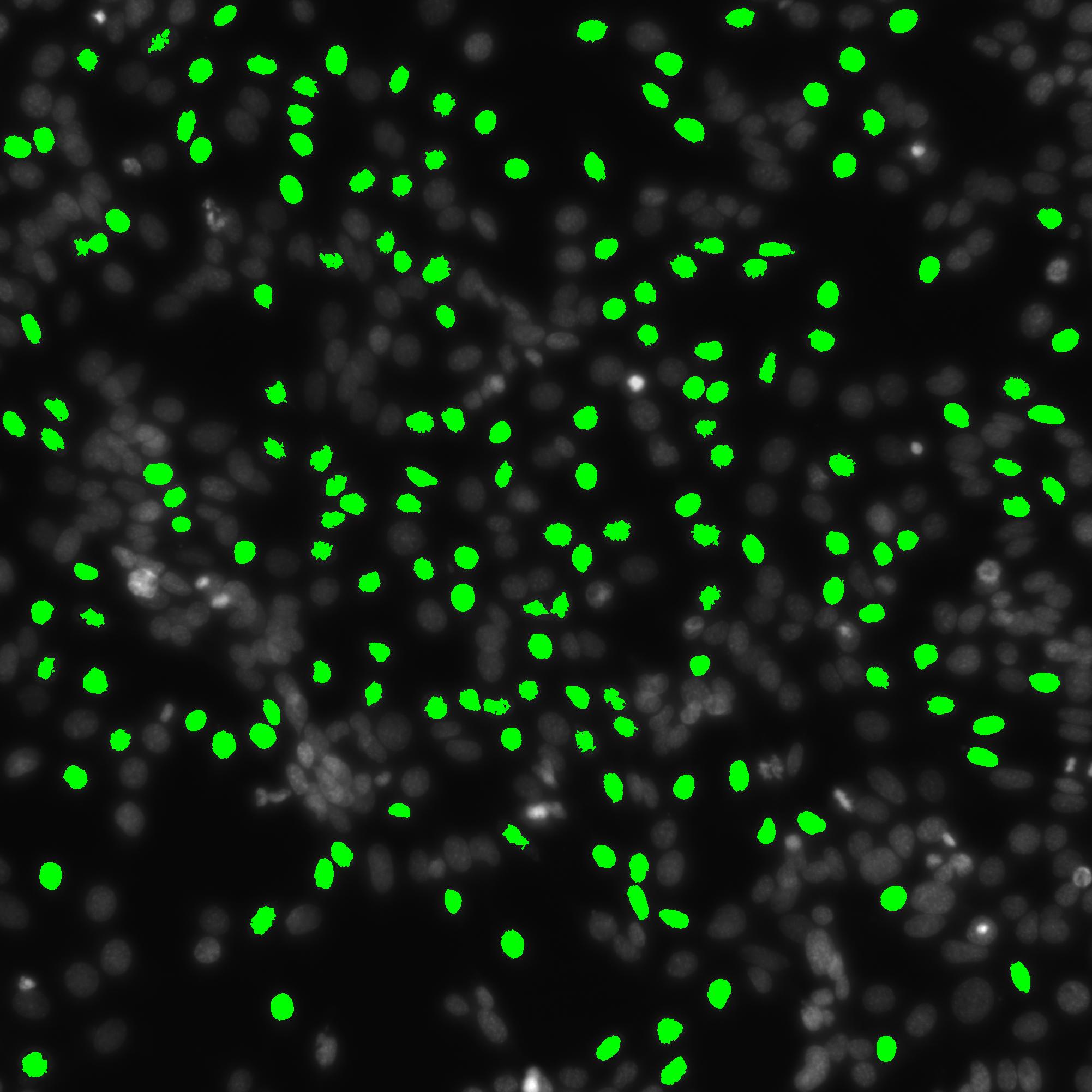

Supplement: S1 File — (ZIP) [file pone.0278130.s006.zip › Supporting Information_Matlab/ExampleData/ScreenWells/AnalyseImages/E04_008_singlenucl.jpg]

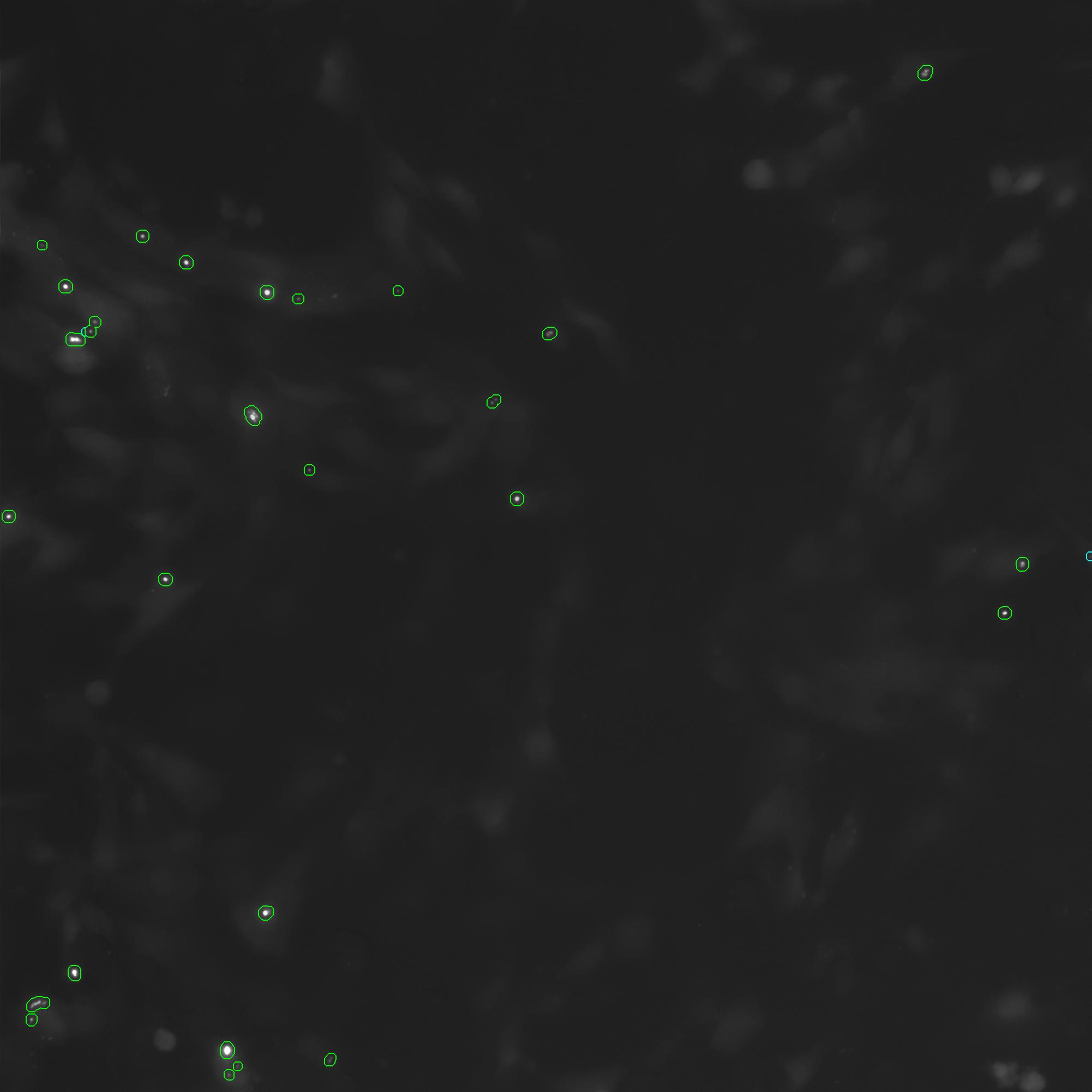

Supplement: S1 File — (ZIP) [file pone.0278130.s006.zip › Supporting Information_Matlab/ExampleData/ScreenWells/AnalyseImages/E04_009_aggr.jpg]

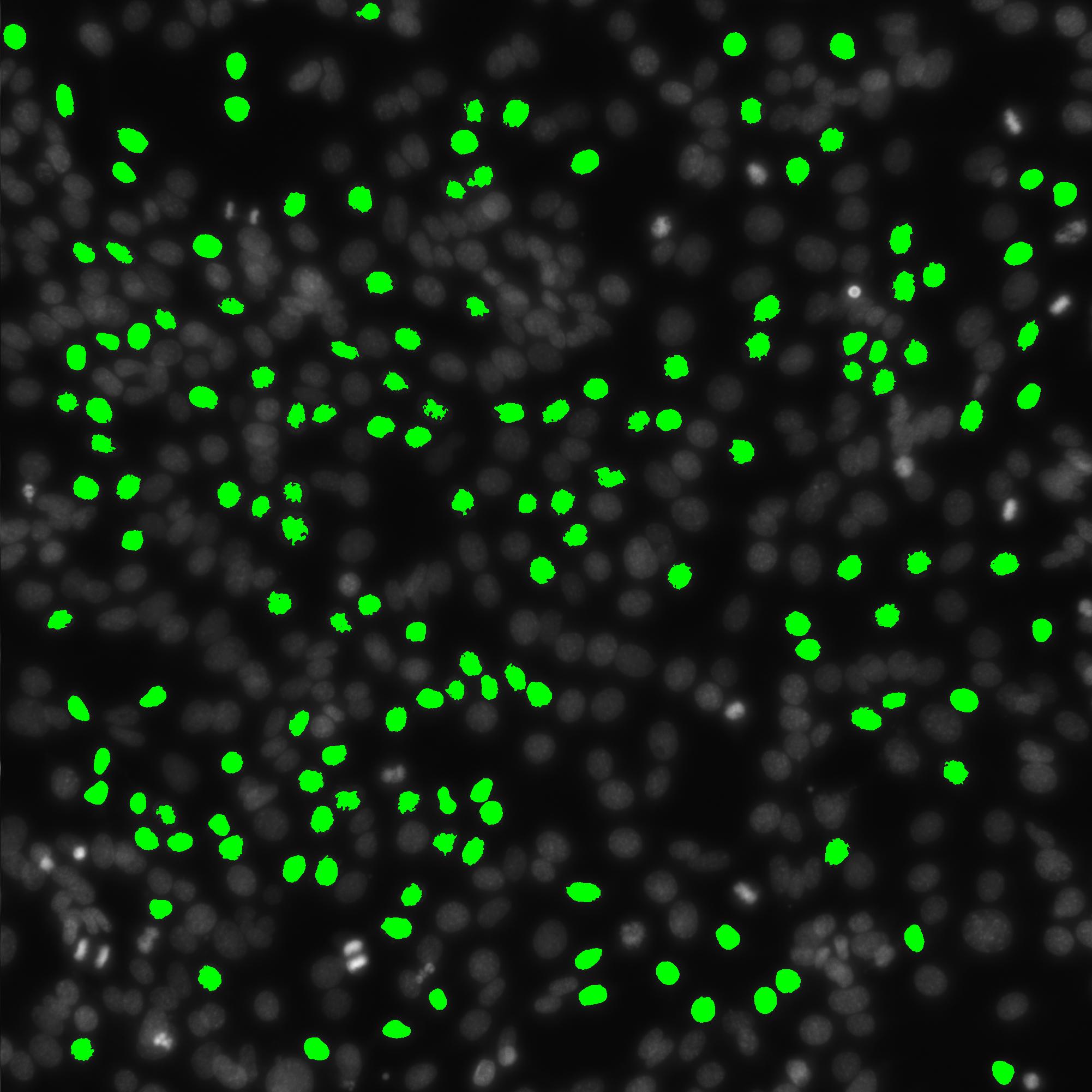

Supplement: S1 File — (ZIP) [file pone.0278130.s006.zip › Supporting Information_Matlab/ExampleData/ScreenWells/AnalyseImages/E04_009_singlenucl.jpg]

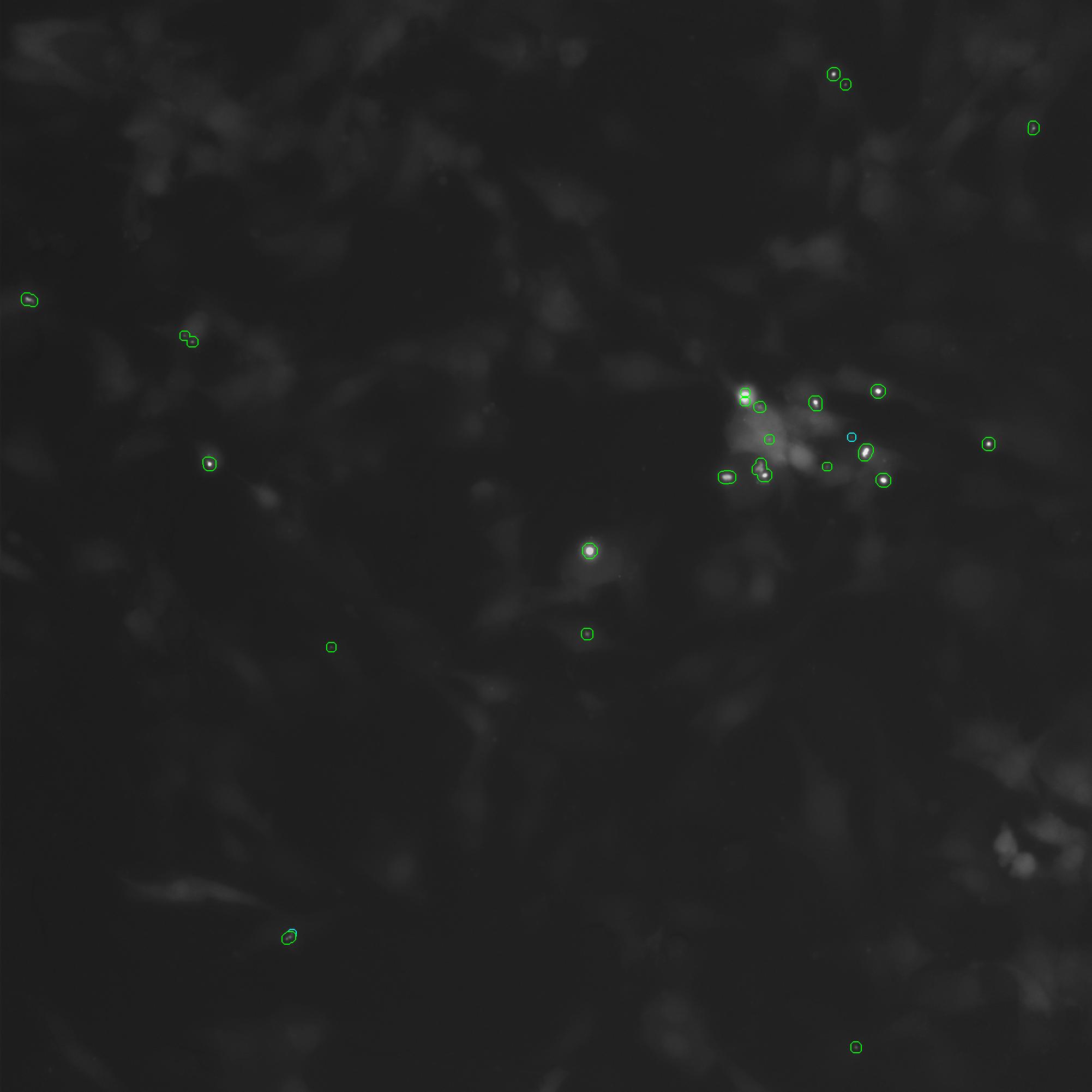

Supplement: S1 File — (ZIP) [file pone.0278130.s006.zip › Supporting Information_Matlab/ExampleData/ScreenWells/AnalyseImages/E04_010_aggr.jpg]

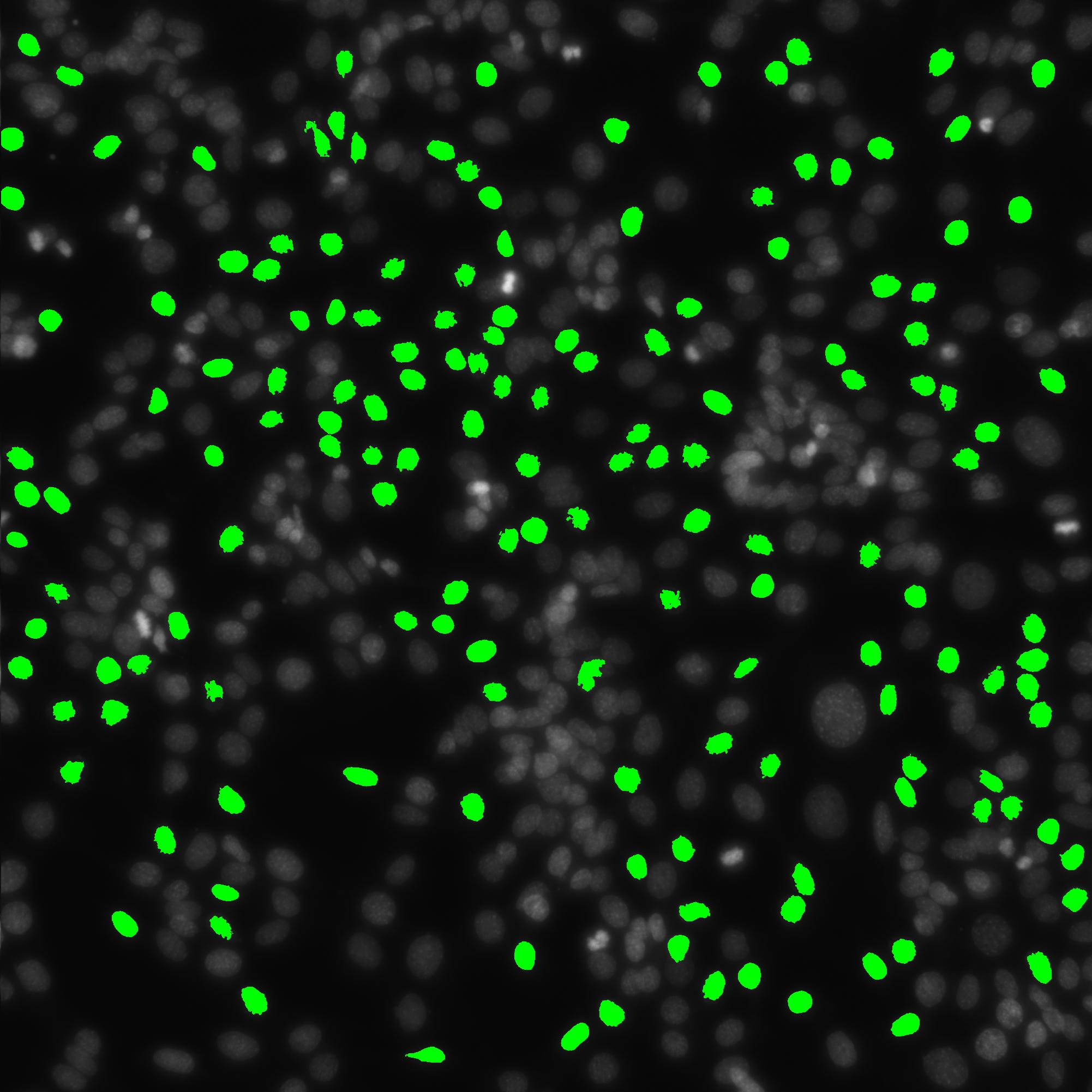

Supplement: S1 File — (ZIP) [file pone.0278130.s006.zip › Supporting Information_Matlab/ExampleData/ScreenWells/AnalyseImages/E04_010_singlenucl.jpg]

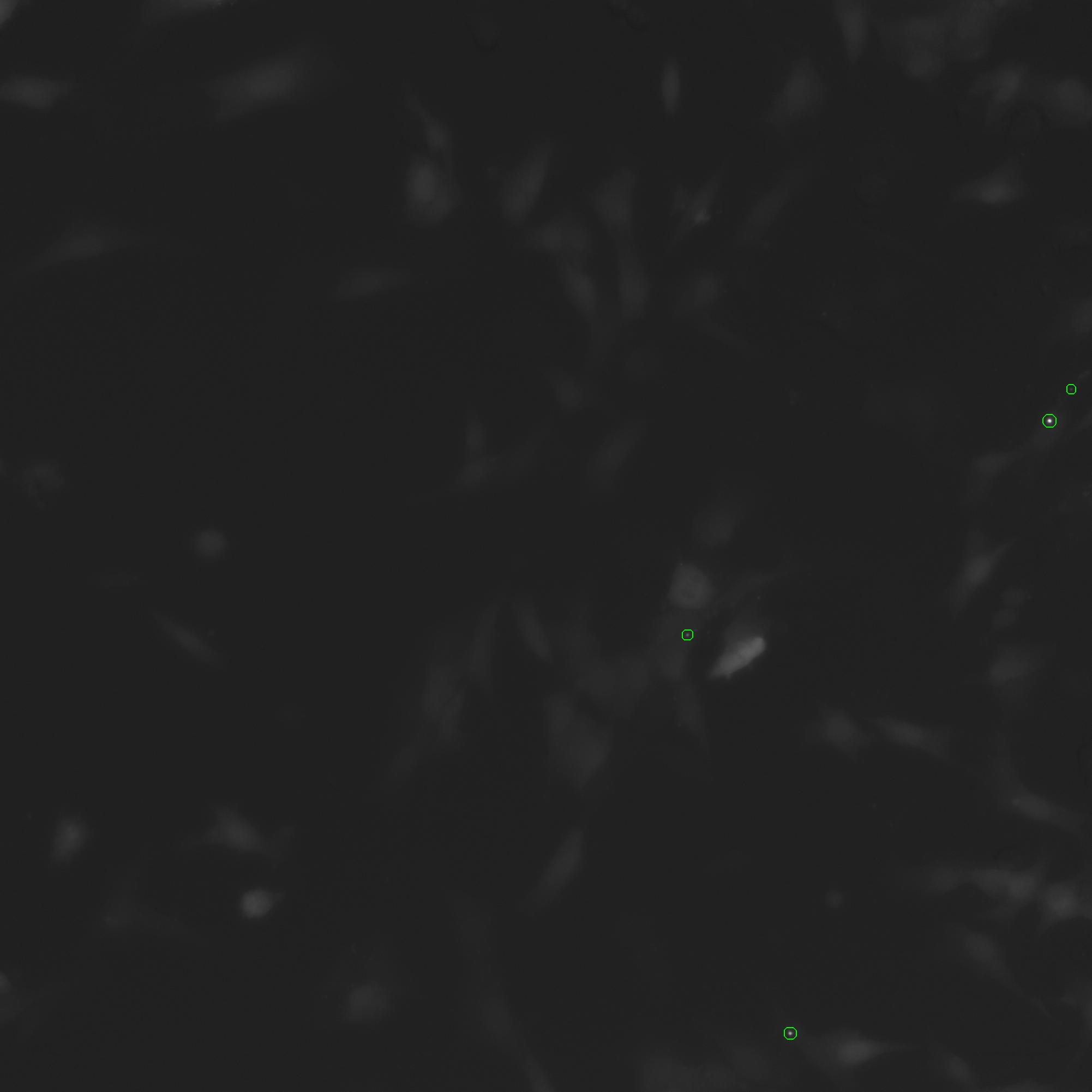

Supplement: S1 File — (ZIP) [file pone.0278130.s006.zip › Supporting Information_Matlab/ExampleData/ScreenWells/AnalyseImages/E04_011_aggr.jpg]

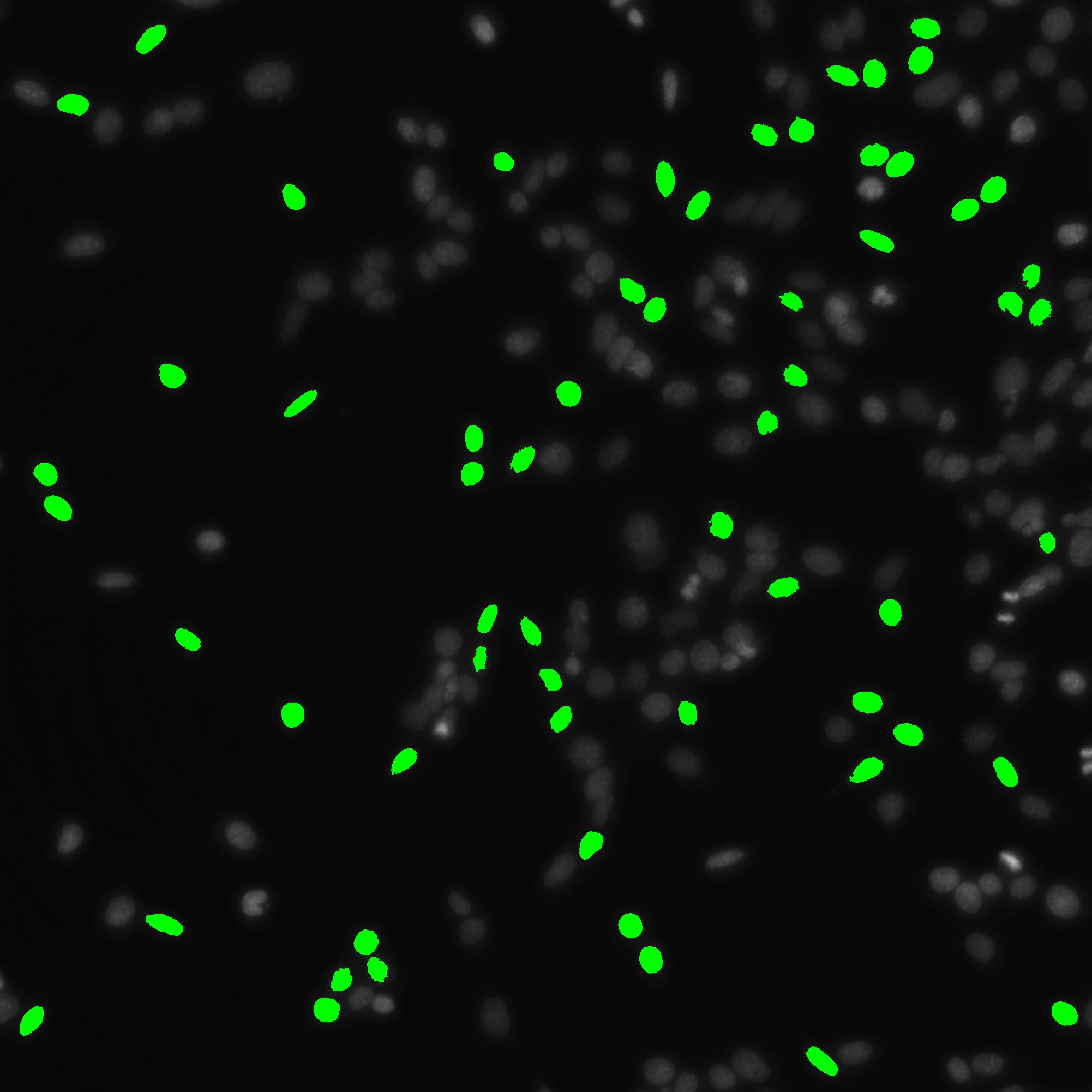

Supplement: S1 File — (ZIP) [file pone.0278130.s006.zip › Supporting Information_Matlab/ExampleData/ScreenWells/AnalyseImages/E04_011_singlenucl.jpg]

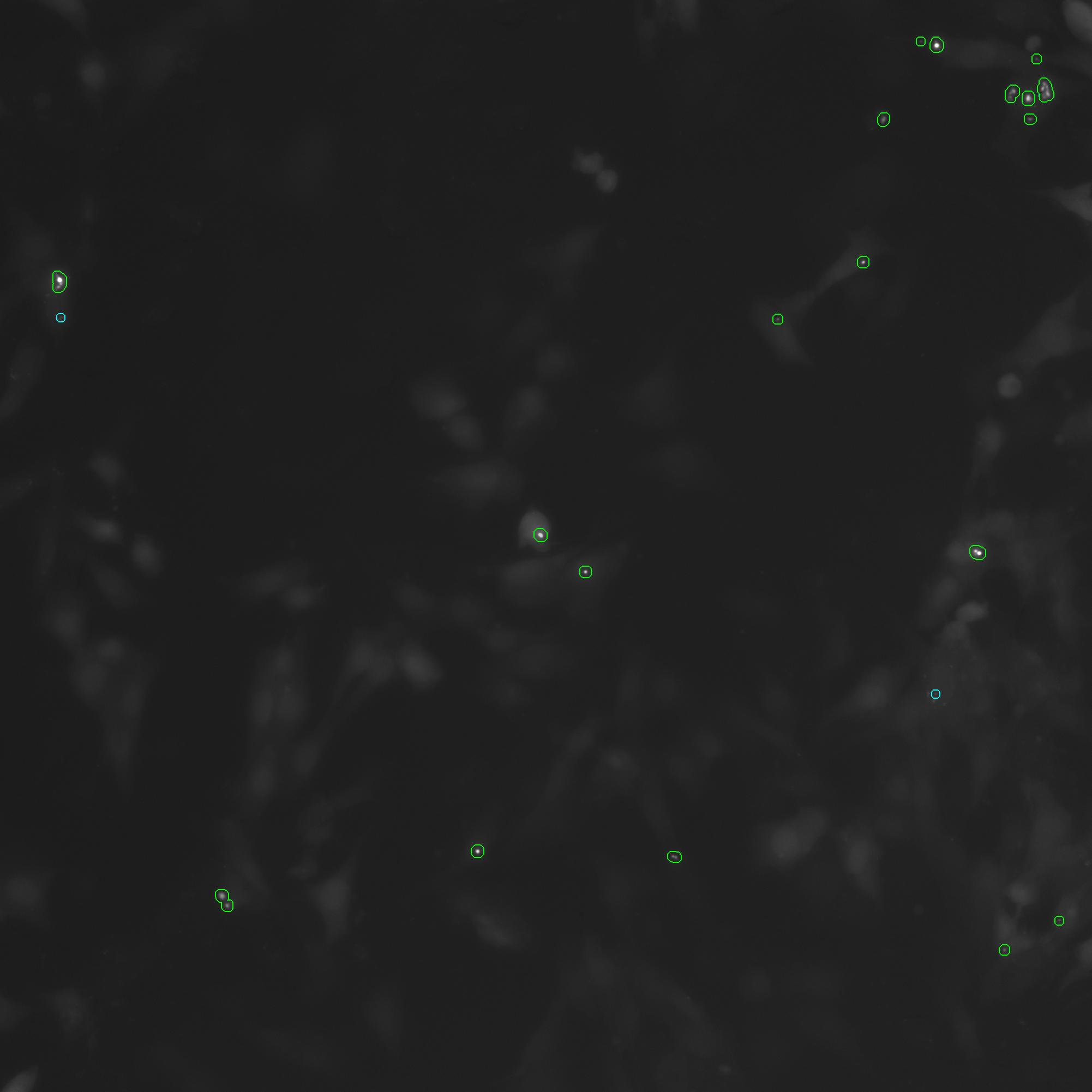

Supplement: S1 File — (ZIP) [file pone.0278130.s006.zip › Supporting Information_Matlab/ExampleData/ScreenWells/AnalyseImages/E04_012_aggr.jpg]

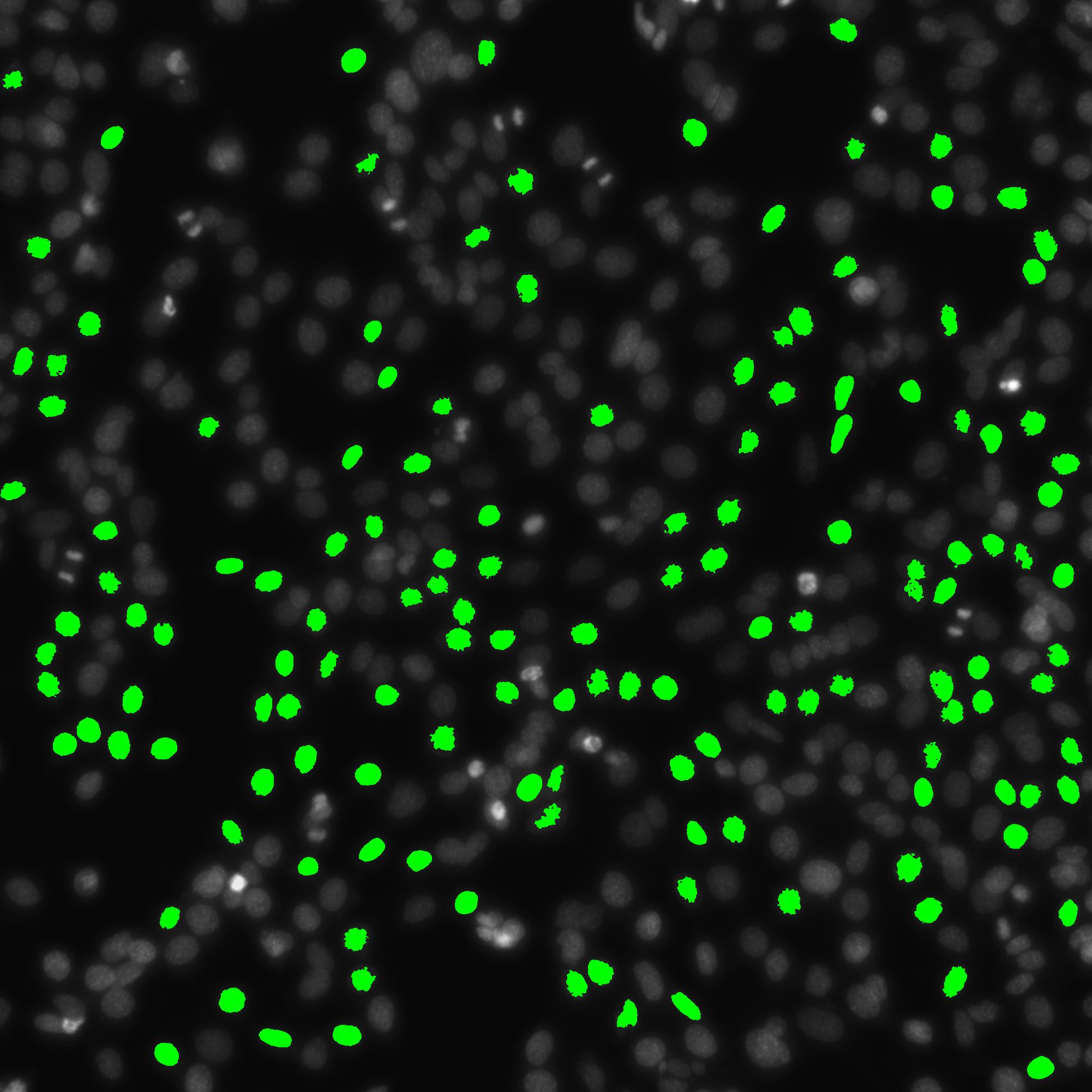

Supplement: S1 File — (ZIP) [file pone.0278130.s006.zip › Supporting Information_Matlab/ExampleData/ScreenWells/AnalyseImages/E04_012_singlenucl.jpg]

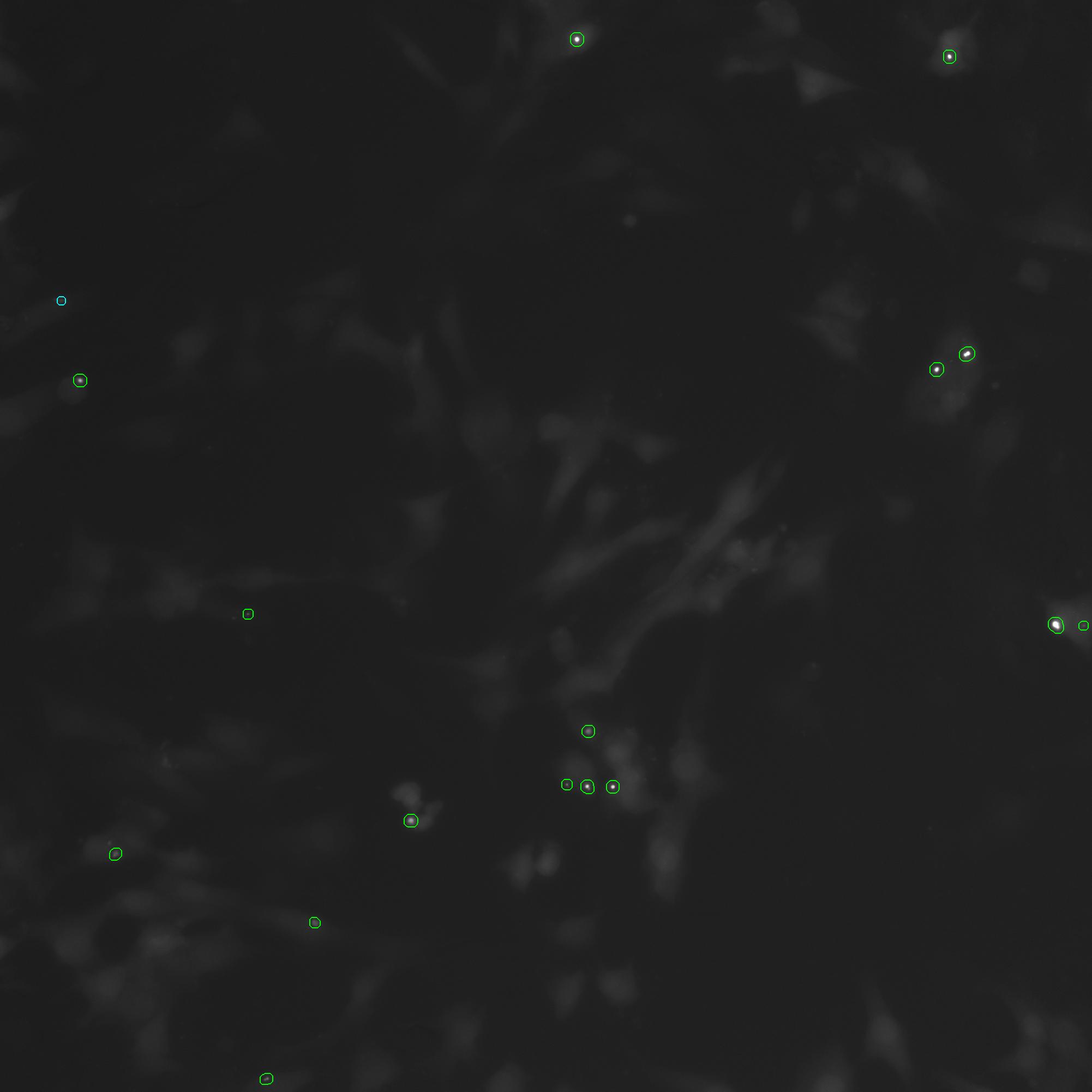

Supplement: S1 File — (ZIP) [file pone.0278130.s006.zip › Supporting Information_Matlab/ExampleData/ScreenWells/AnalyseImages/E04_013_aggr.jpg]

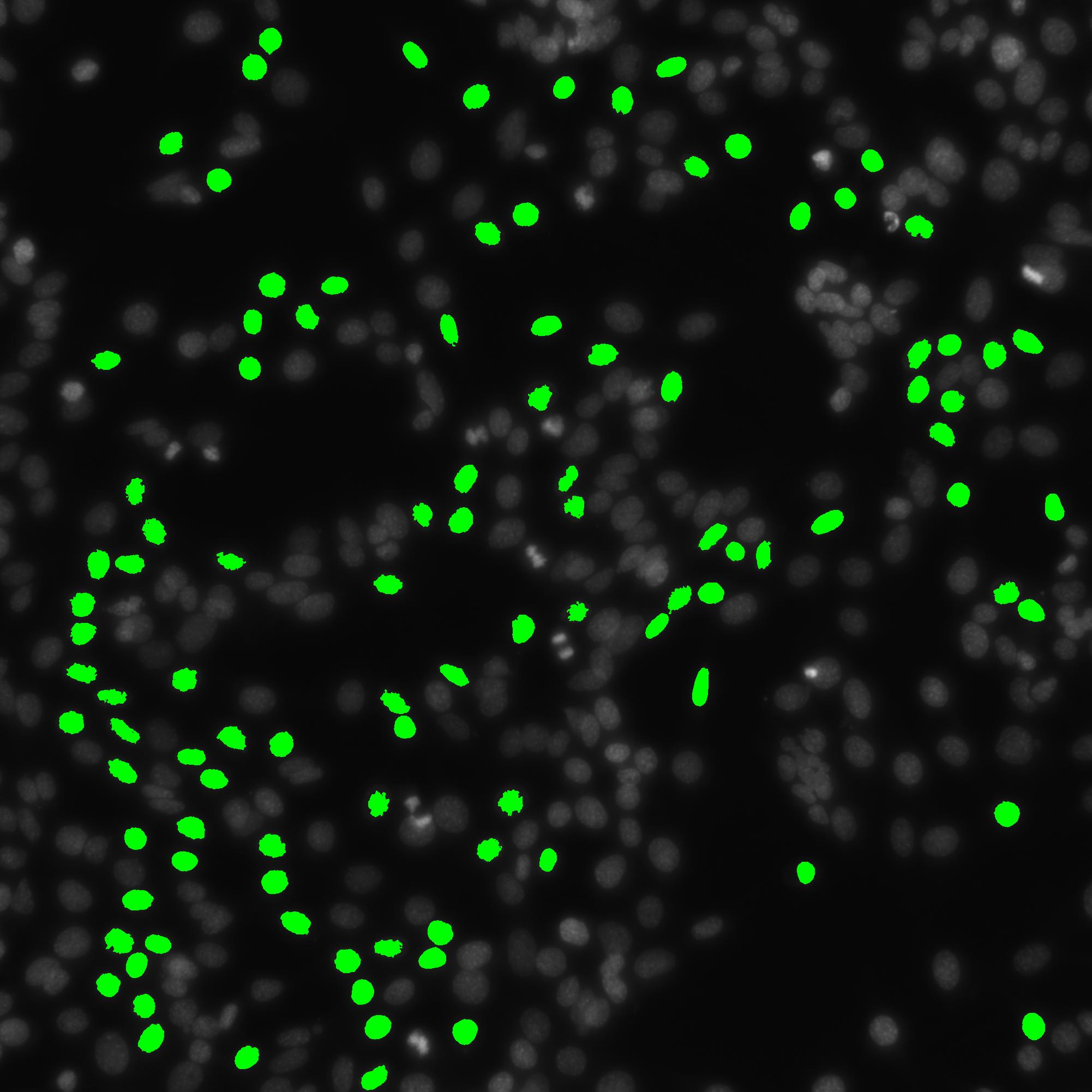

Supplement: S1 File — (ZIP) [file pone.0278130.s006.zip › Supporting Information_Matlab/ExampleData/ScreenWells/AnalyseImages/E04_013_singlenucl.jpg]

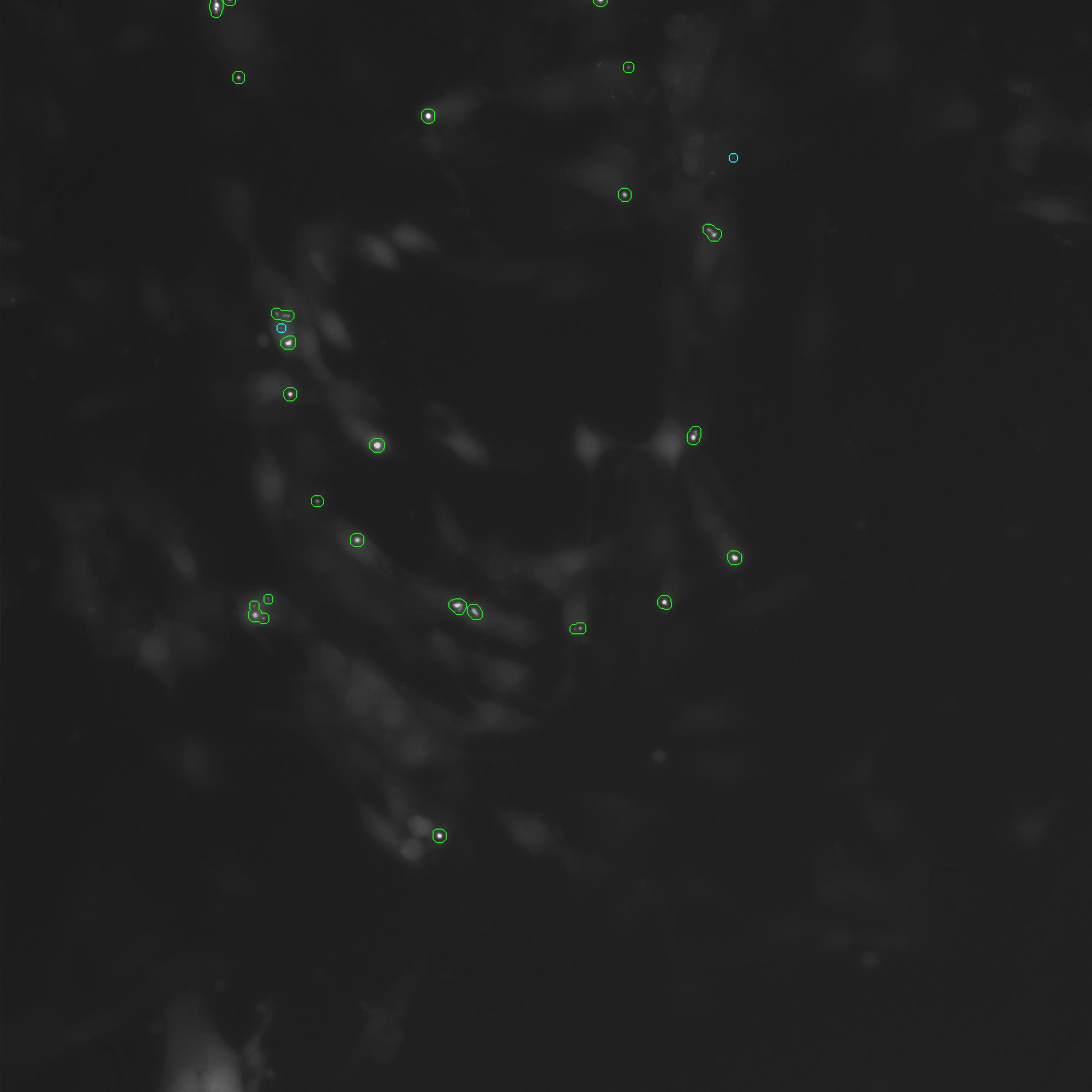

Supplement: S1 File — (ZIP) [file pone.0278130.s006.zip › Supporting Information_Matlab/ExampleData/ScreenWells/AnalyseImages/E04_014_aggr.jpg]

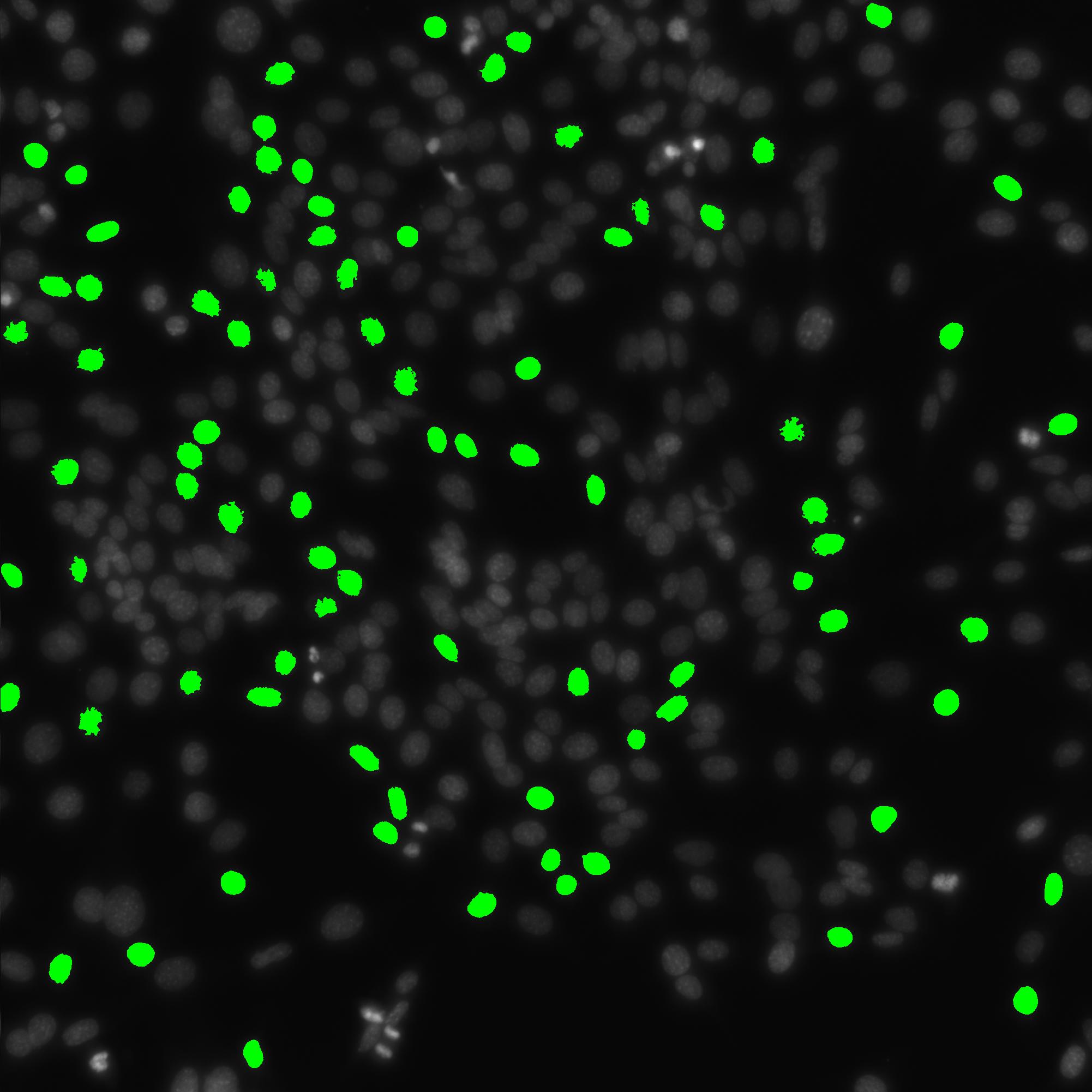

Supplement: S1 File — (ZIP) [file pone.0278130.s006.zip › Supporting Information_Matlab/ExampleData/ScreenWells/AnalyseImages/E04_014_singlenucl.jpg]

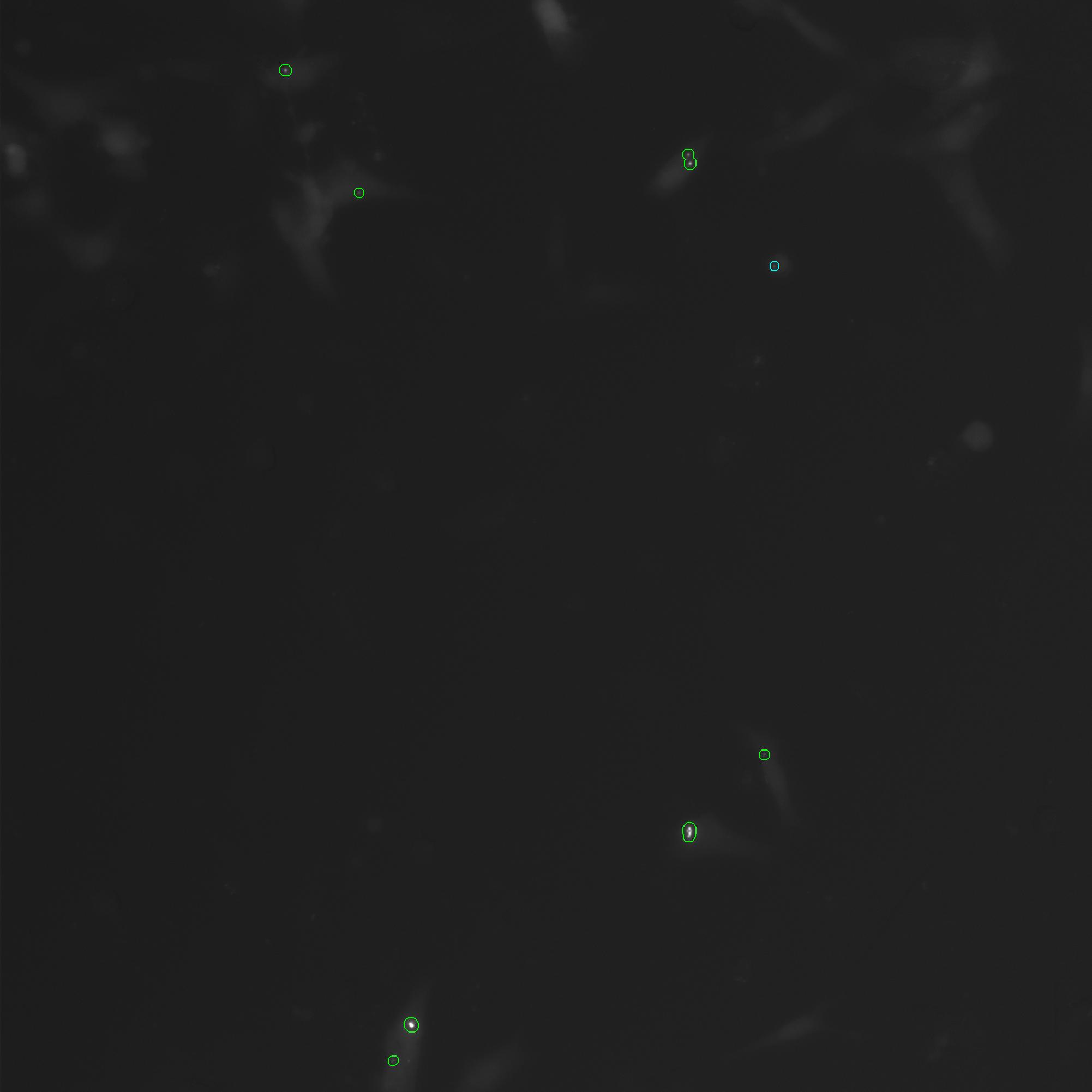

Supplement: S1 File — (ZIP) [file pone.0278130.s006.zip › Supporting Information_Matlab/ExampleData/ScreenWells/AnalyseImages/E04_015_aggr.jpg]

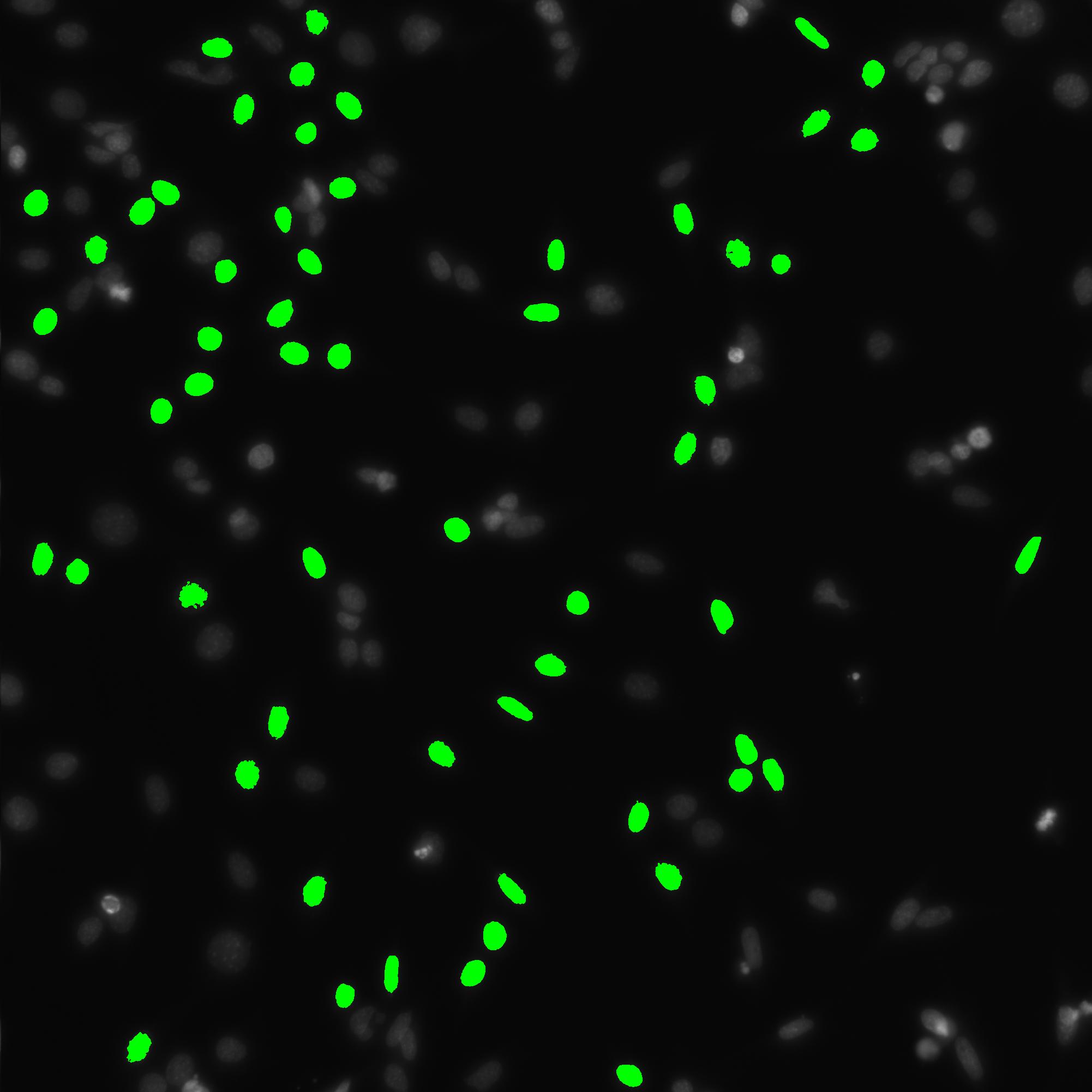

Supplement: S1 File — (ZIP) [file pone.0278130.s006.zip › Supporting Information_Matlab/ExampleData/ScreenWells/AnalyseImages/E04_015_singlenucl.jpg]

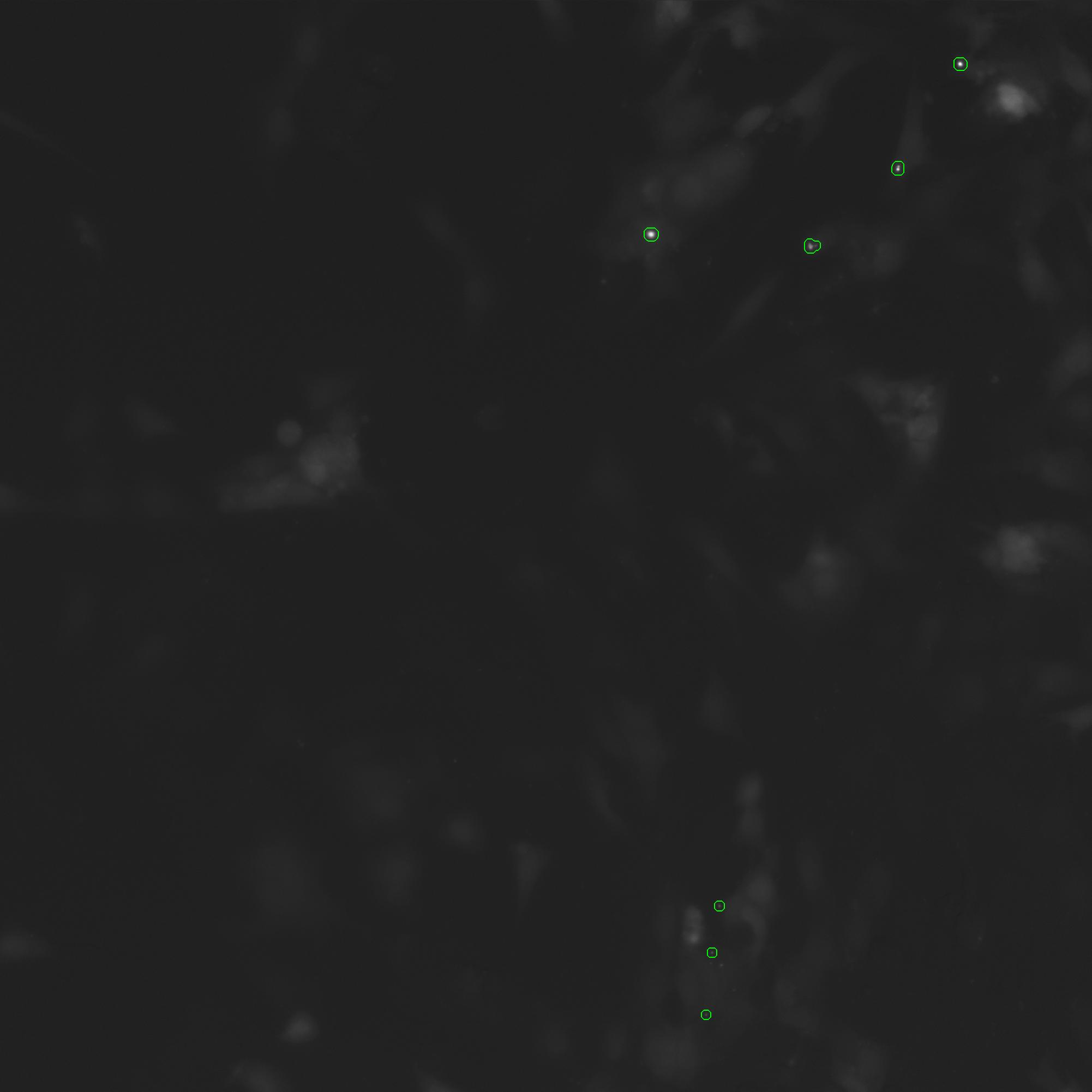

Supplement: S1 File — (ZIP) [file pone.0278130.s006.zip › Supporting Information_Matlab/ExampleData/ScreenWells/AnalyseImages/E04_016_aggr.jpg]

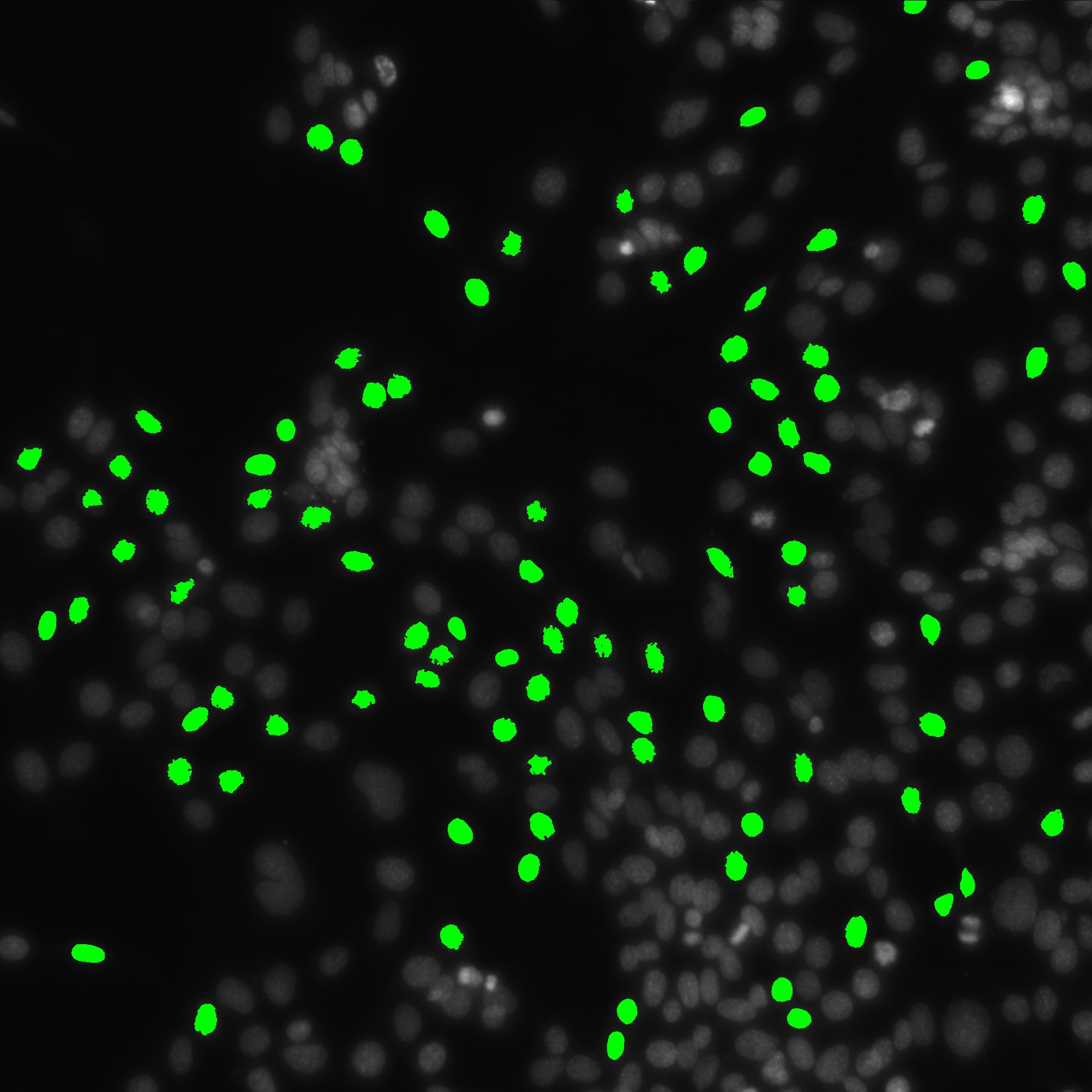

Supplement: S1 File — (ZIP) [file pone.0278130.s006.zip › Supporting Information_Matlab/ExampleData/ScreenWells/AnalyseImages/E04_016_singlenucl.jpg]

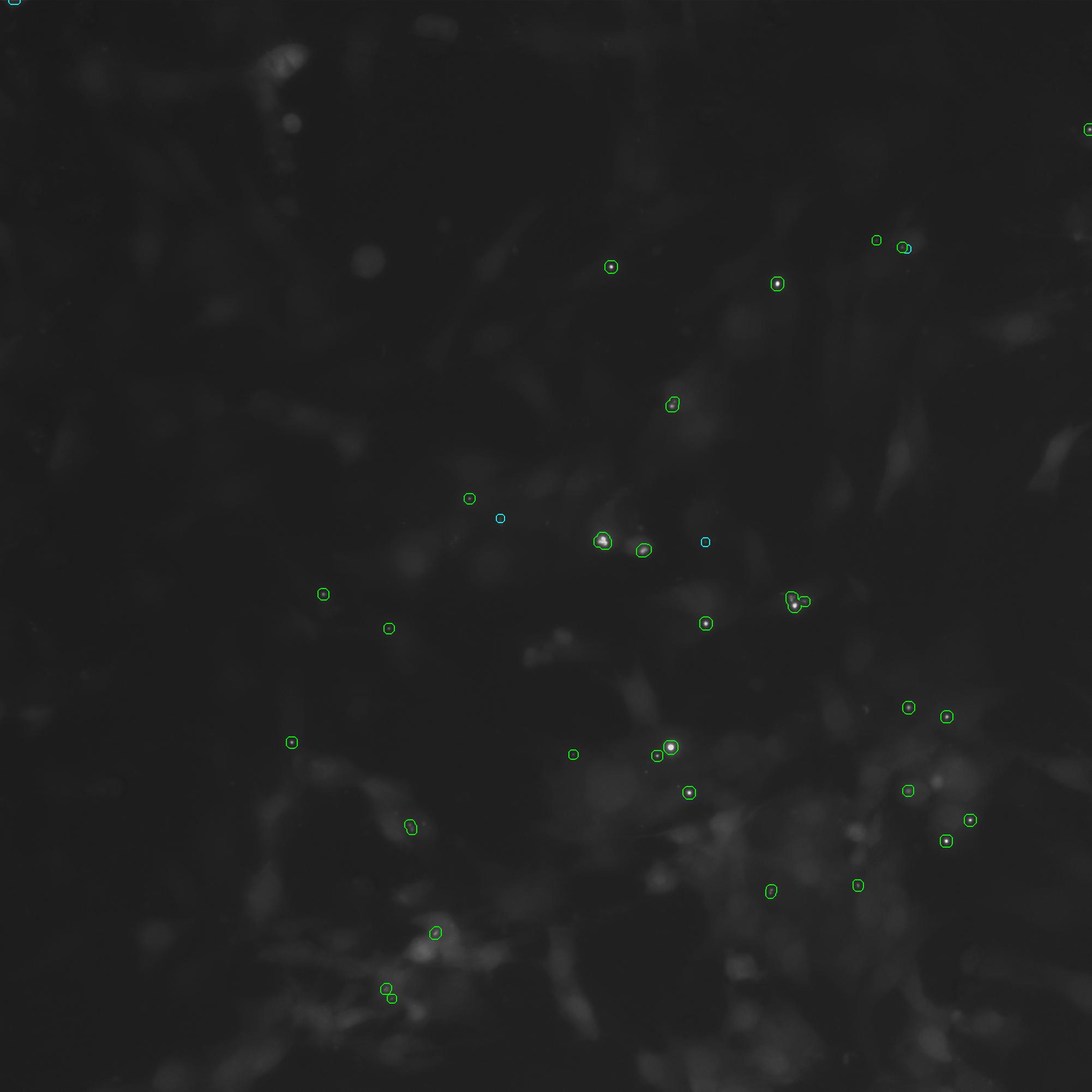

Supplement: S1 File — (ZIP) [file pone.0278130.s006.zip › Supporting Information_Matlab/ExampleData/ScreenWells/AnalyseImages/E04_017_aggr.jpg]

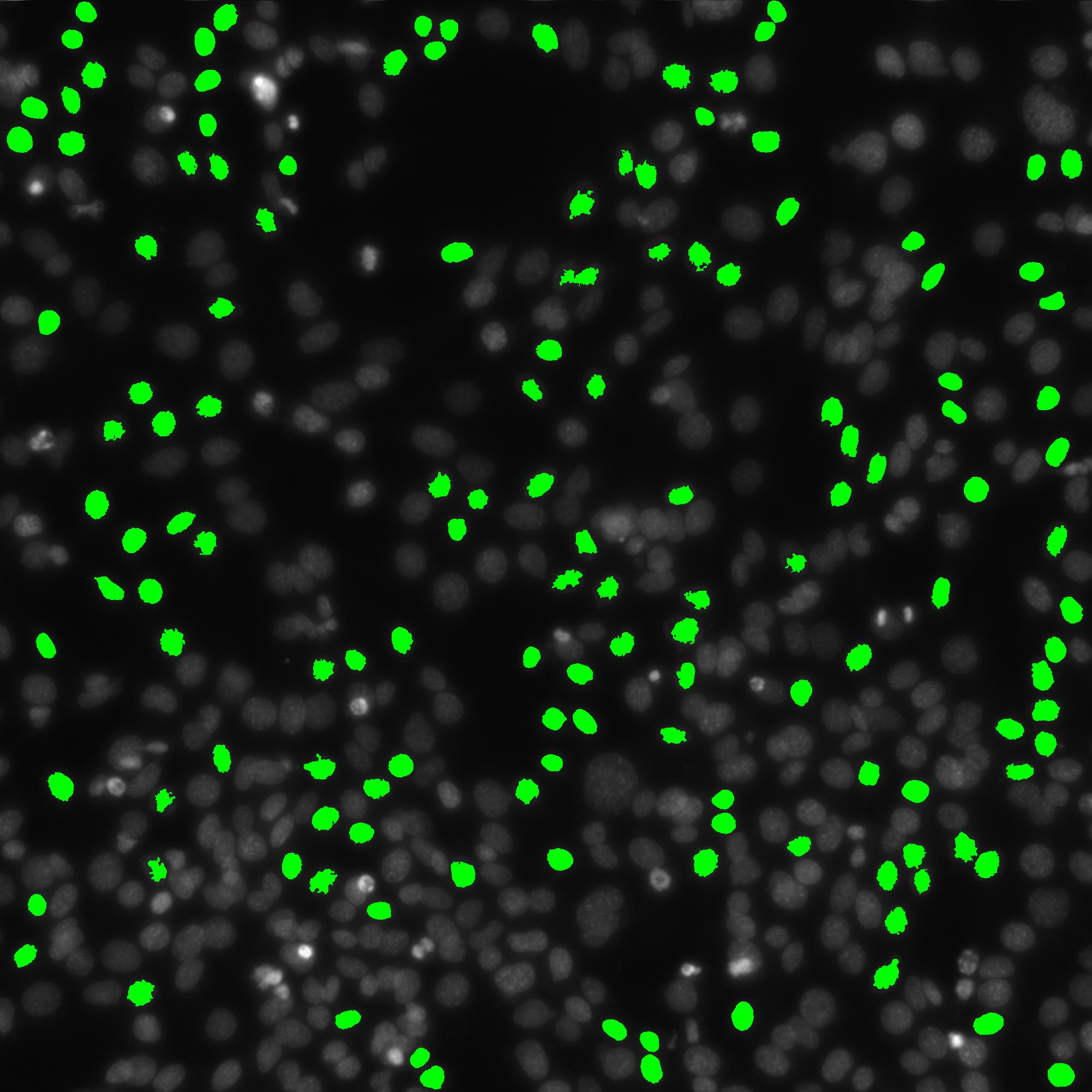

Supplement: S1 File — (ZIP) [file pone.0278130.s006.zip › Supporting Information_Matlab/ExampleData/ScreenWells/AnalyseImages/E04_017_singlenucl.jpg]

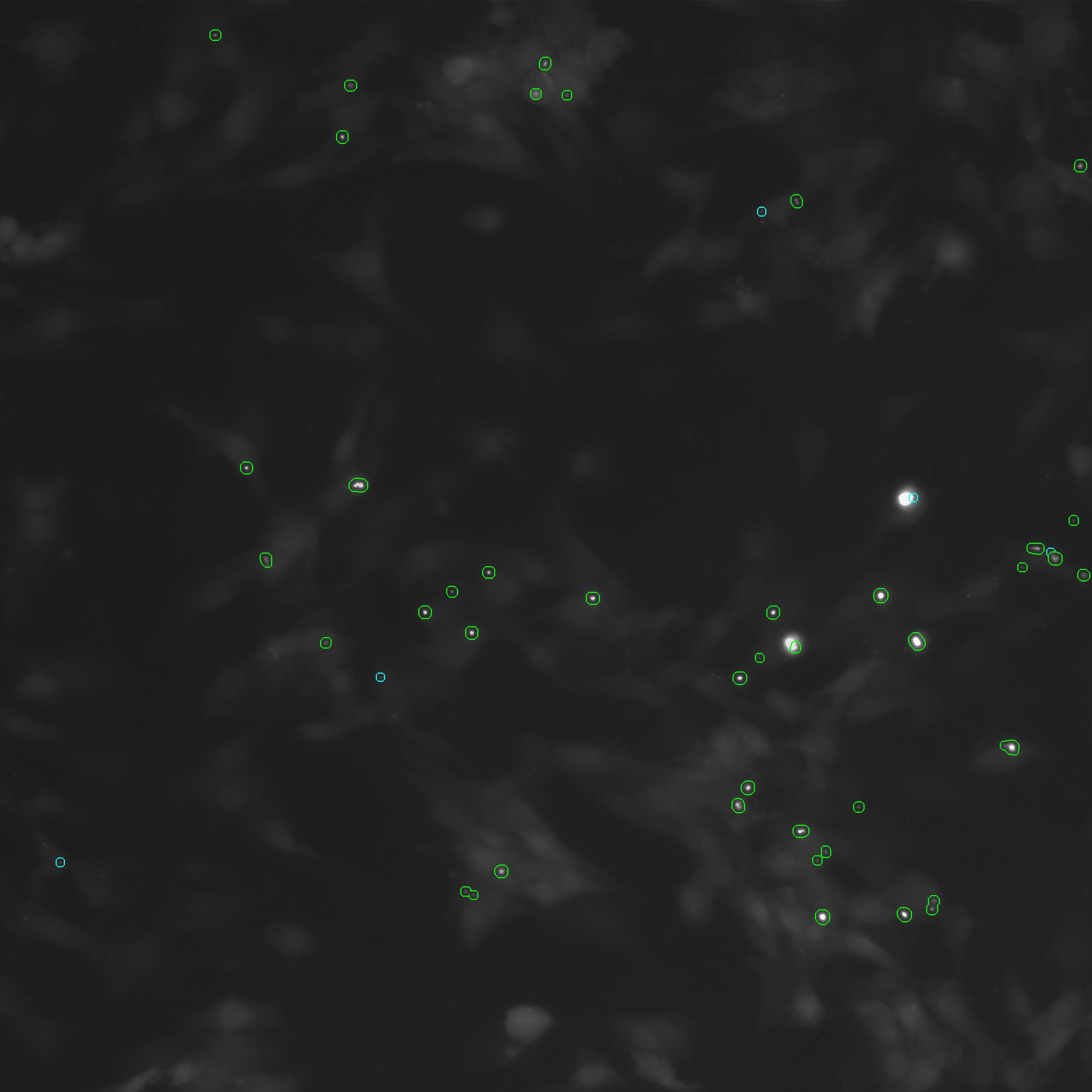

Supplement: S1 File — (ZIP) [file pone.0278130.s006.zip › Supporting Information_Matlab/ExampleData/ScreenWells/AnalyseImages/E04_018_aggr.jpg]

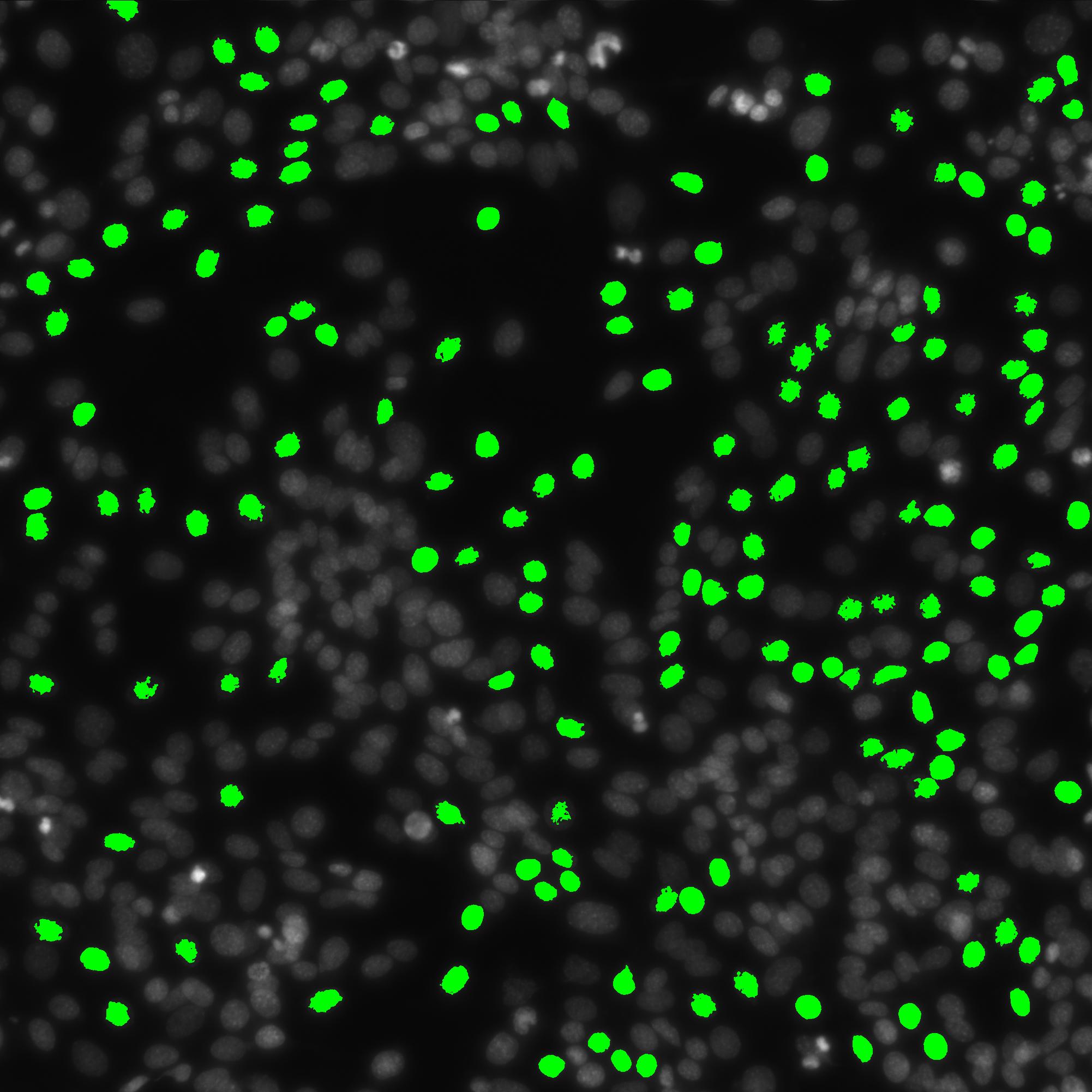

Supplement: S1 File — (ZIP) [file pone.0278130.s006.zip › Supporting Information_Matlab/ExampleData/ScreenWells/AnalyseImages/E04_018_singlenucl.jpg]

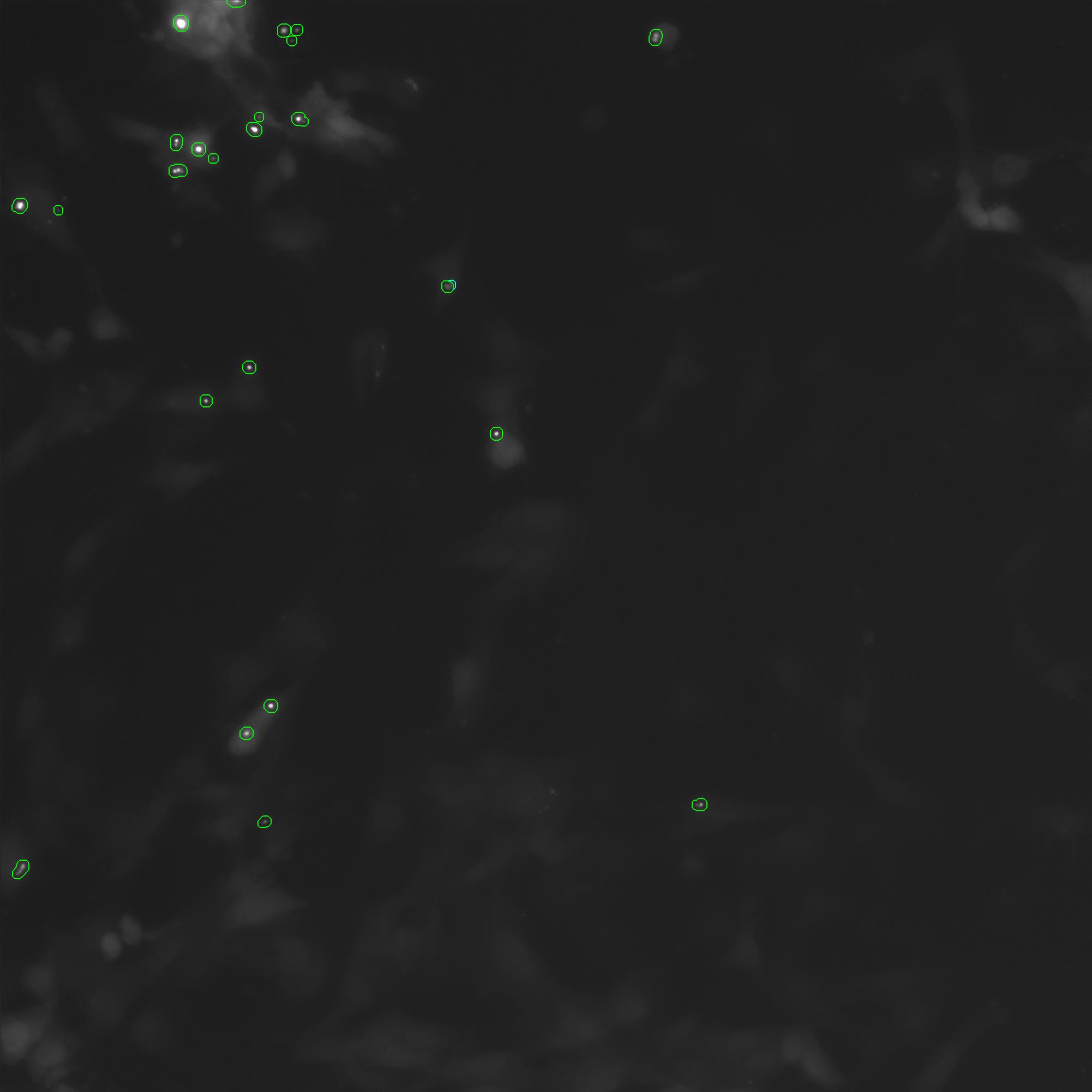

Supplement: S1 File — (ZIP) [file pone.0278130.s006.zip › Supporting Information_Matlab/ExampleData/ScreenWells/AnalyseImages/E04_019_aggr.jpg]

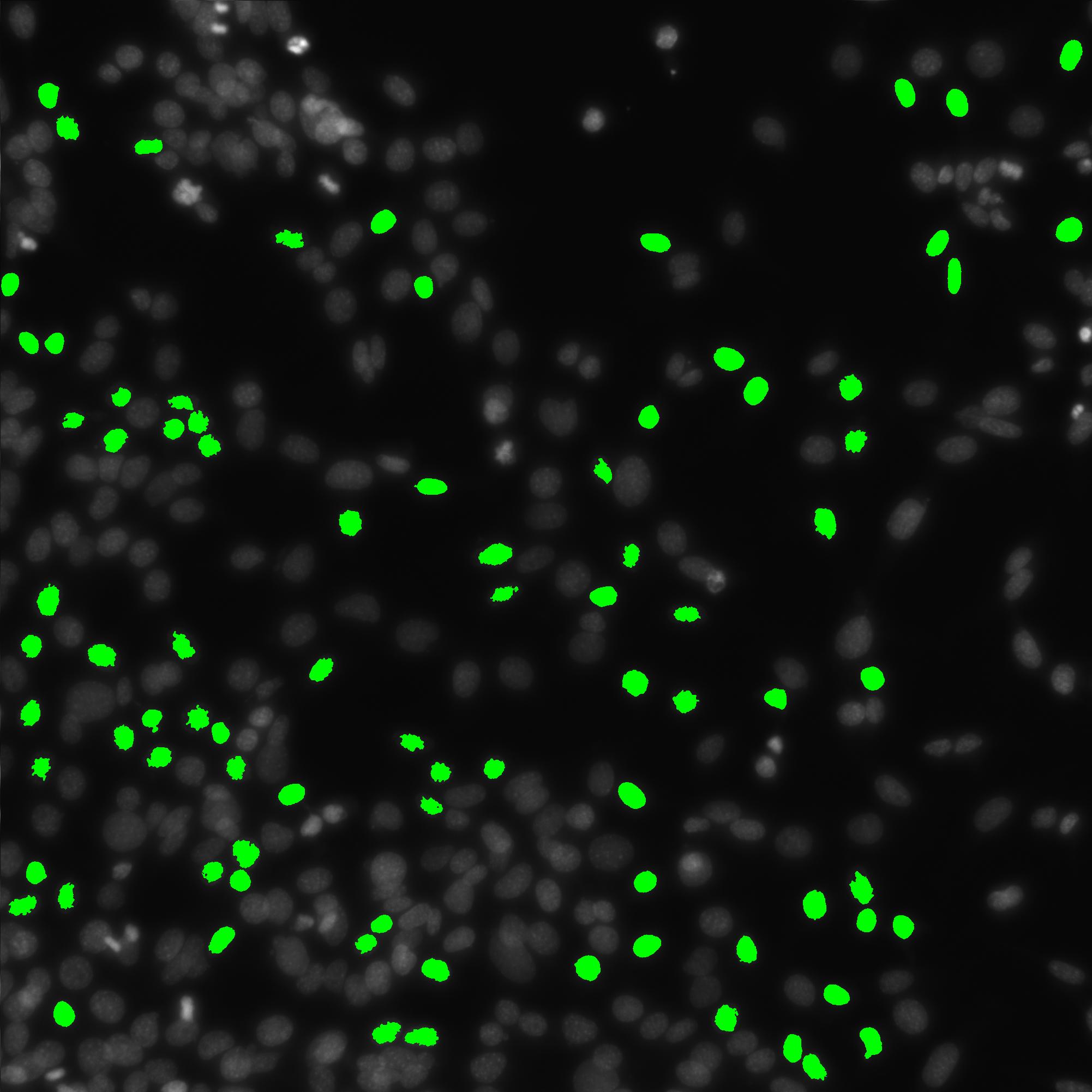

Supplement: S1 File — (ZIP) [file pone.0278130.s006.zip › Supporting Information_Matlab/ExampleData/ScreenWells/AnalyseImages/E04_019_singlenucl.jpg]

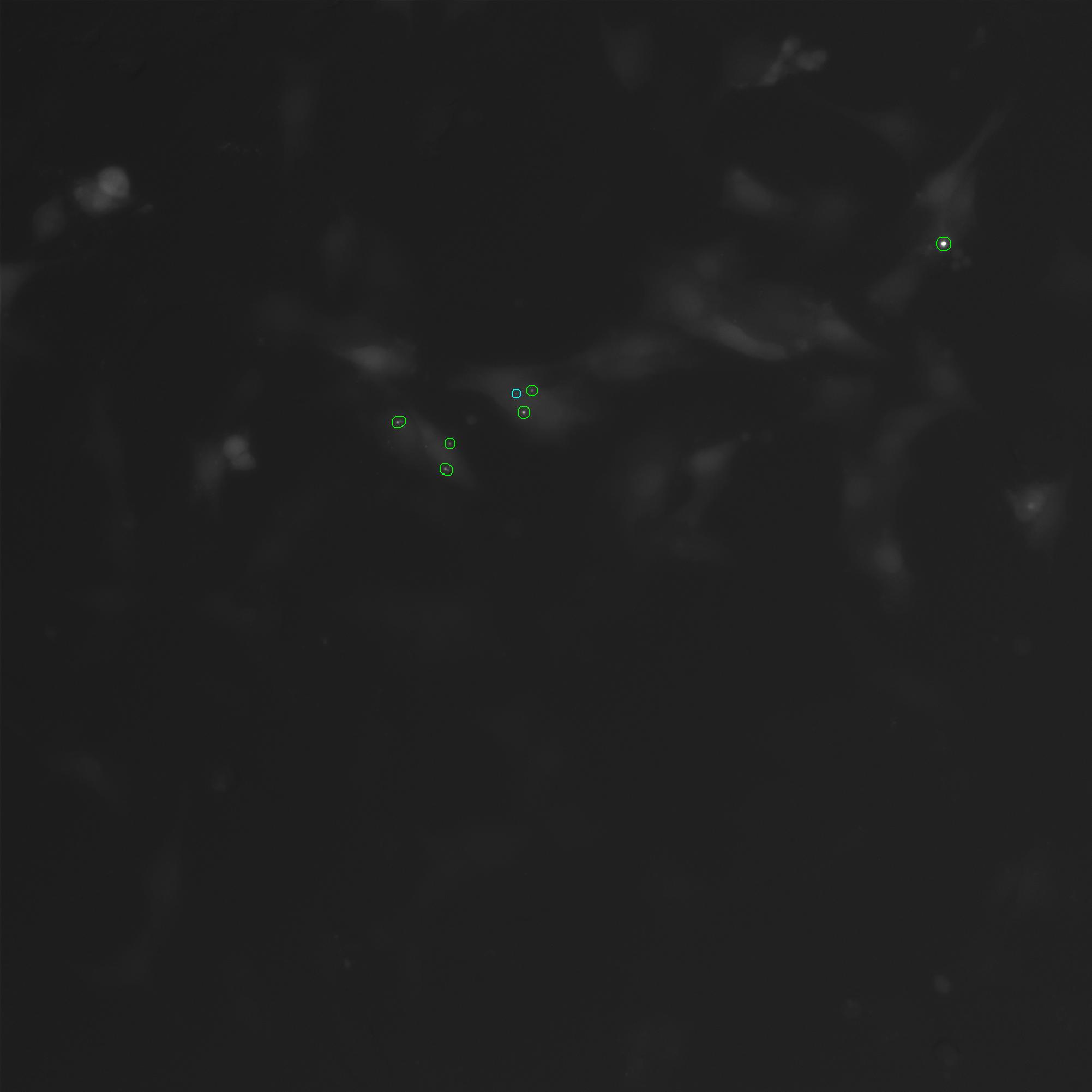

Supplement: S1 File — (ZIP) [file pone.0278130.s006.zip › Supporting Information_Matlab/ExampleData/ScreenWells/AnalyseImages/E04_020_aggr.jpg]

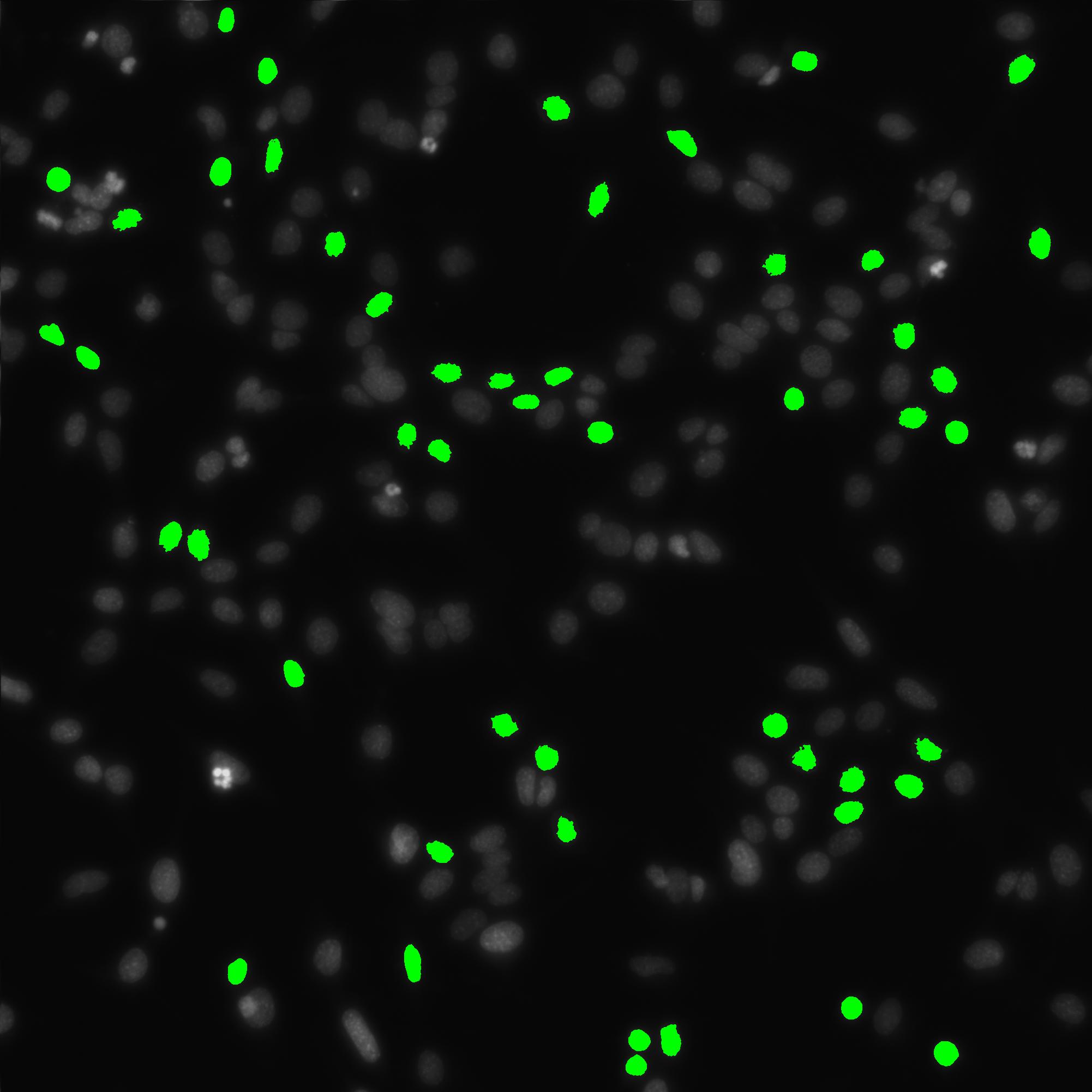

Supplement: S1 File — (ZIP) [file pone.0278130.s006.zip › Supporting Information_Matlab/ExampleData/ScreenWells/AnalyseImages/E04_020_singlenucl.jpg]

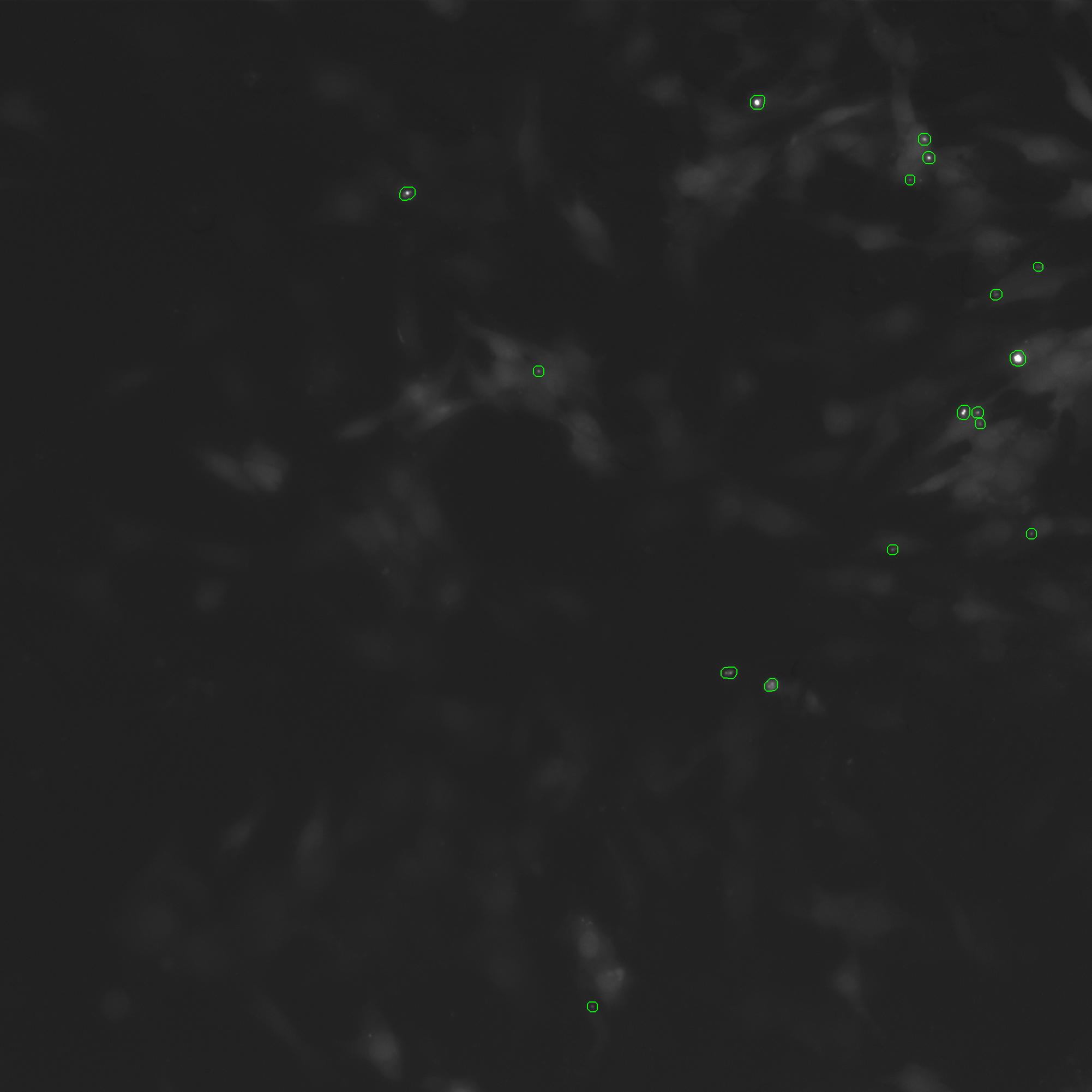

Supplement: S1 File — (ZIP) [file pone.0278130.s006.zip › Supporting Information_Matlab/ExampleData/ScreenWells/AnalyseImages/E04_021_aggr.jpg]

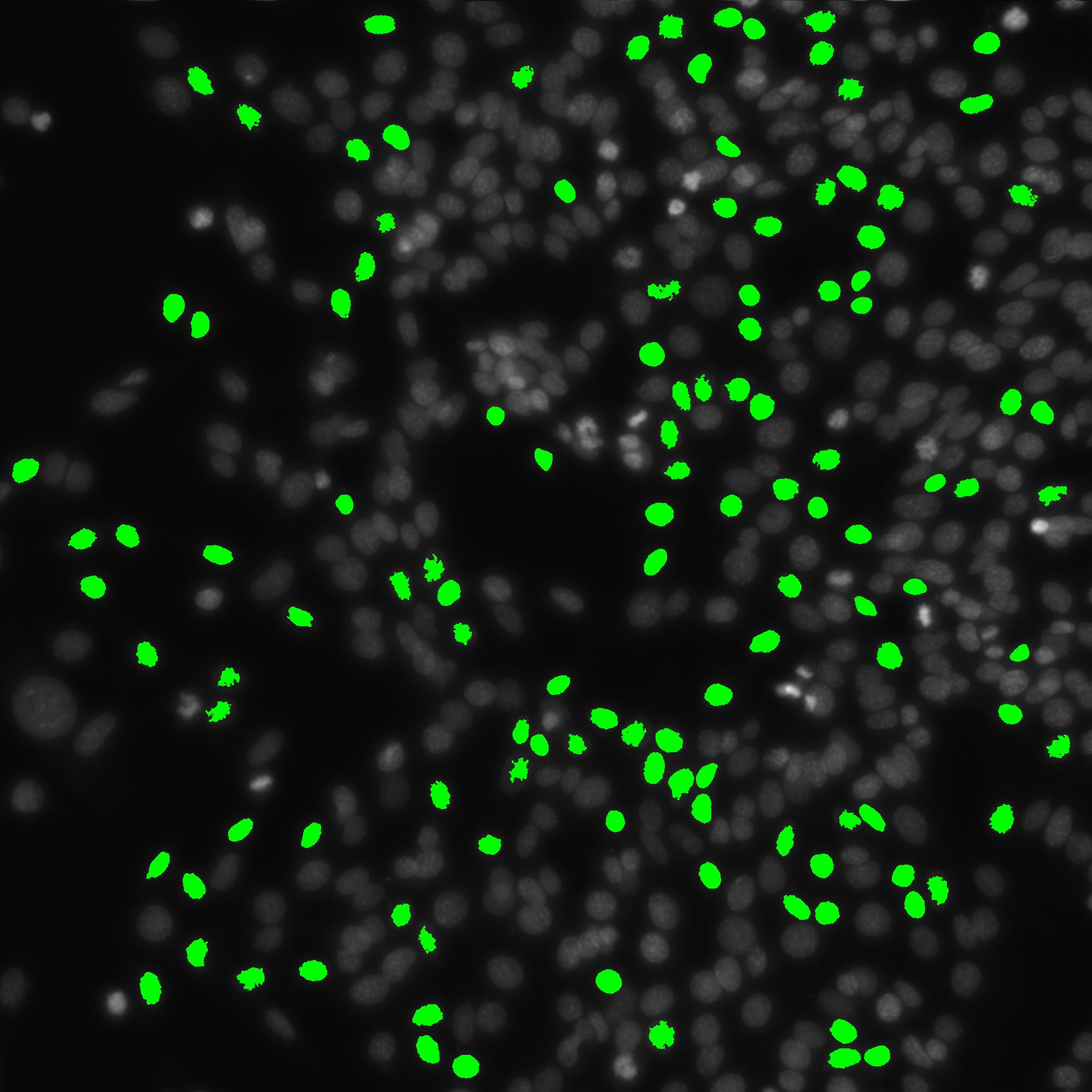

Supplement: S1 File — (ZIP) [file pone.0278130.s006.zip › Supporting Information_Matlab/ExampleData/ScreenWells/AnalyseImages/E04_021_singlenucl.jpg]

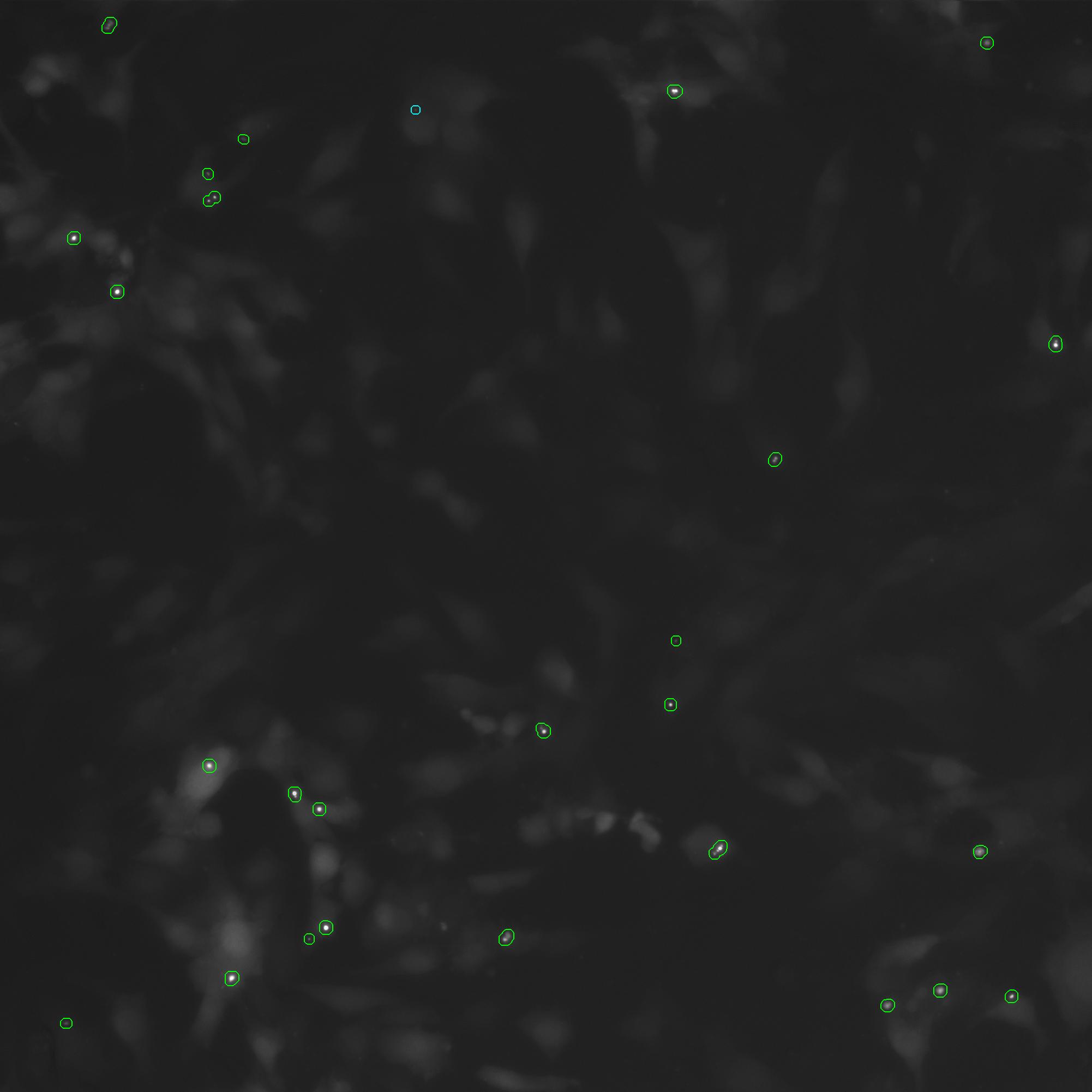

Supplement: S1 File — (ZIP) [file pone.0278130.s006.zip › Supporting Information_Matlab/ExampleData/ScreenWells/AnalyseImages/E04_022_aggr.jpg]

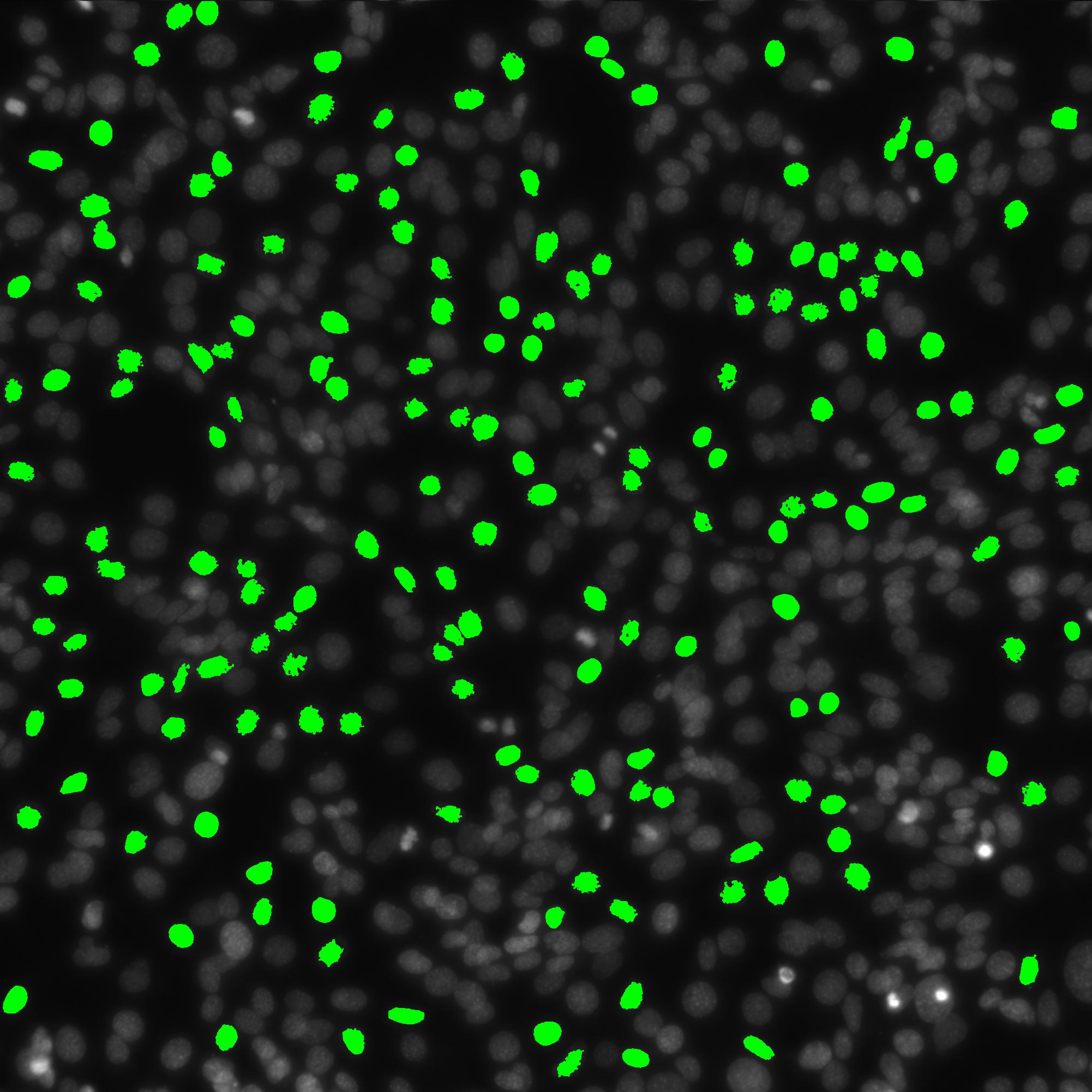

Supplement: S1 File — (ZIP) [file pone.0278130.s006.zip › Supporting Information_Matlab/ExampleData/ScreenWells/AnalyseImages/E04_022_singlenucl.jpg]

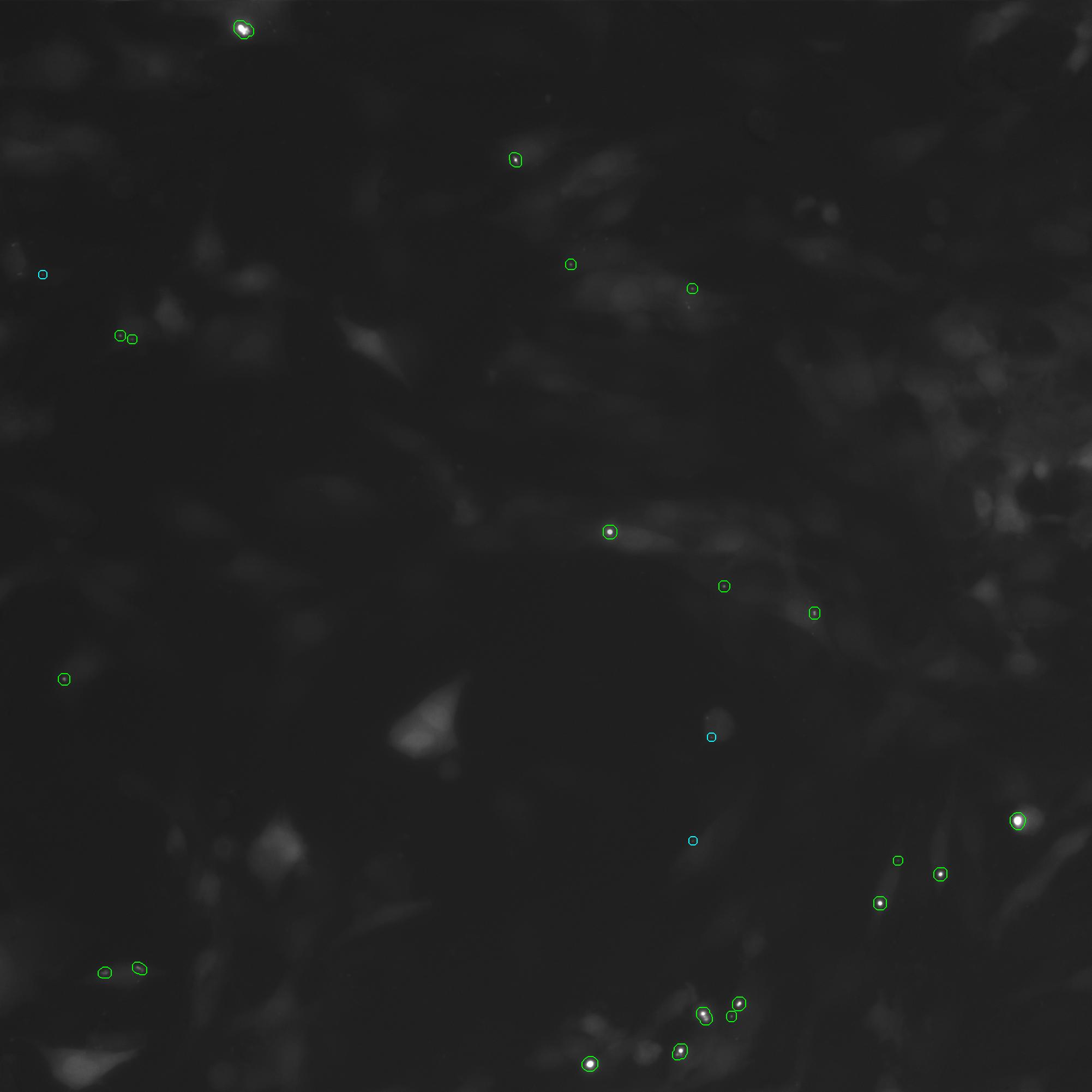

Supplement: S1 File — (ZIP) [file pone.0278130.s006.zip › Supporting Information_Matlab/ExampleData/ScreenWells/AnalyseImages/E04_023_aggr.jpg]

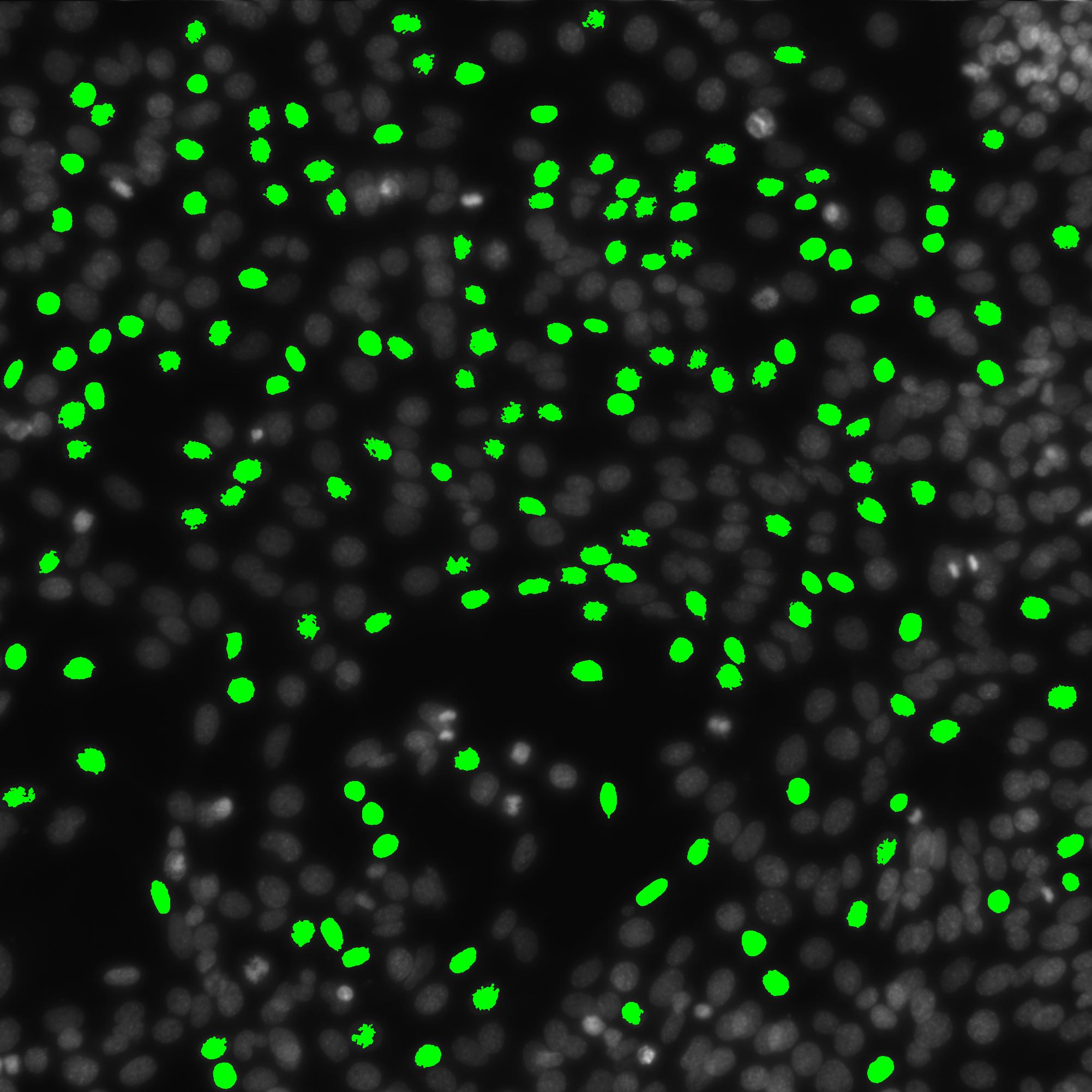

Supplement: S1 File — (ZIP) [file pone.0278130.s006.zip › Supporting Information_Matlab/ExampleData/ScreenWells/AnalyseImages/E04_023_singlenucl.jpg]

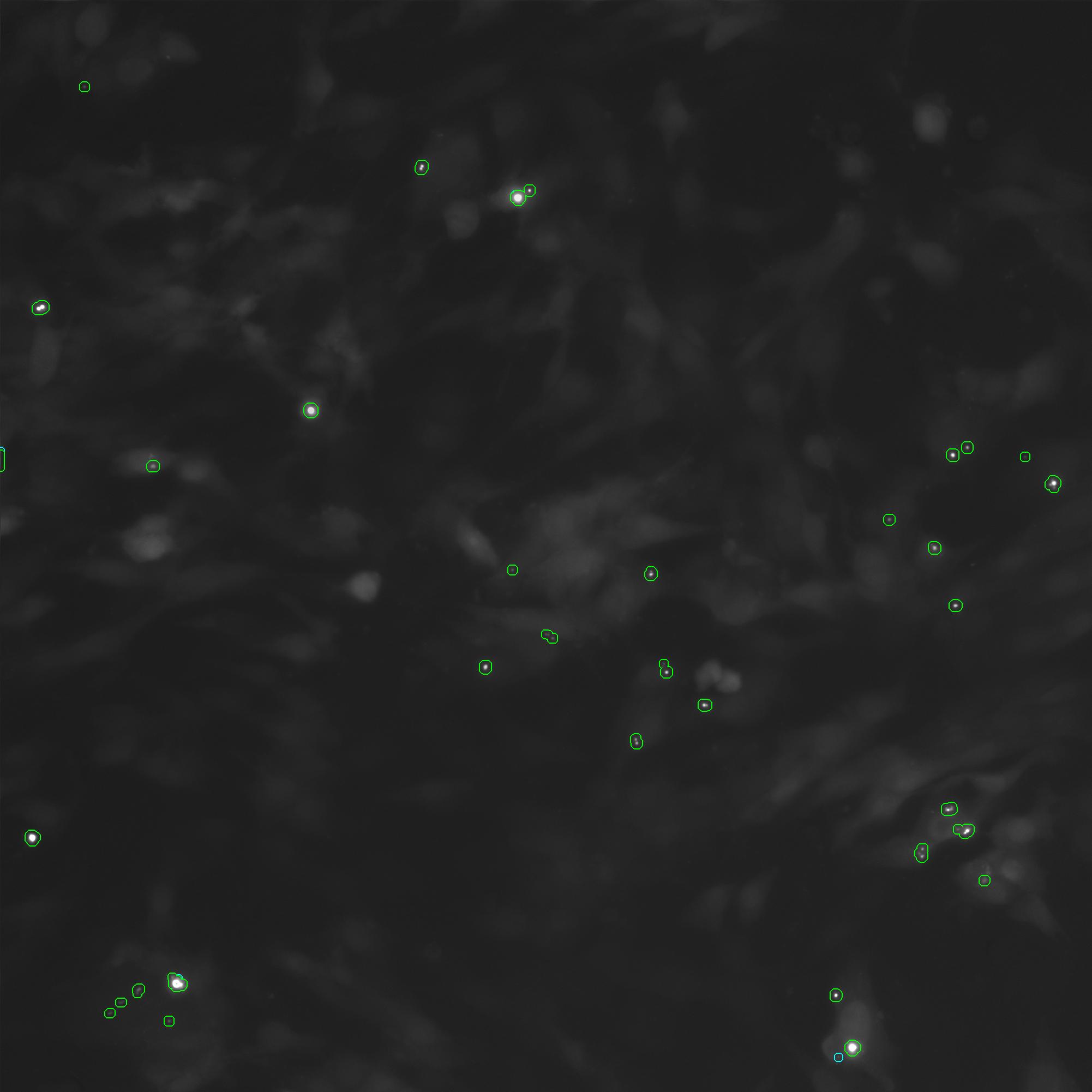

Supplement: S1 File — (ZIP) [file pone.0278130.s006.zip › Supporting Information_Matlab/ExampleData/ScreenWells/AnalyseImages/E04_024_aggr.jpg]

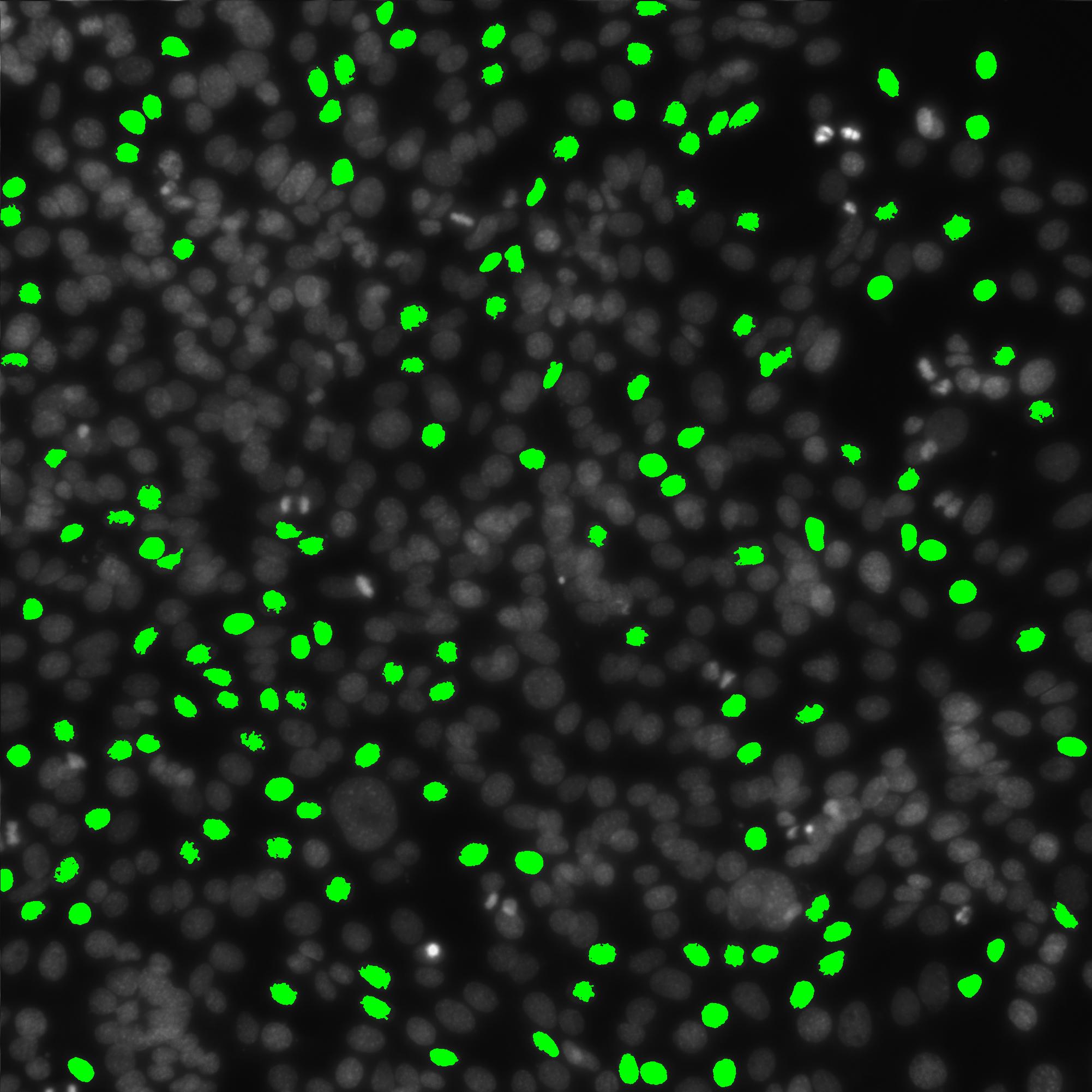

Supplement: S1 File — (ZIP) [file pone.0278130.s006.zip › Supporting Information_Matlab/ExampleData/ScreenWells/AnalyseImages/E04_024_singlenucl.jpg]

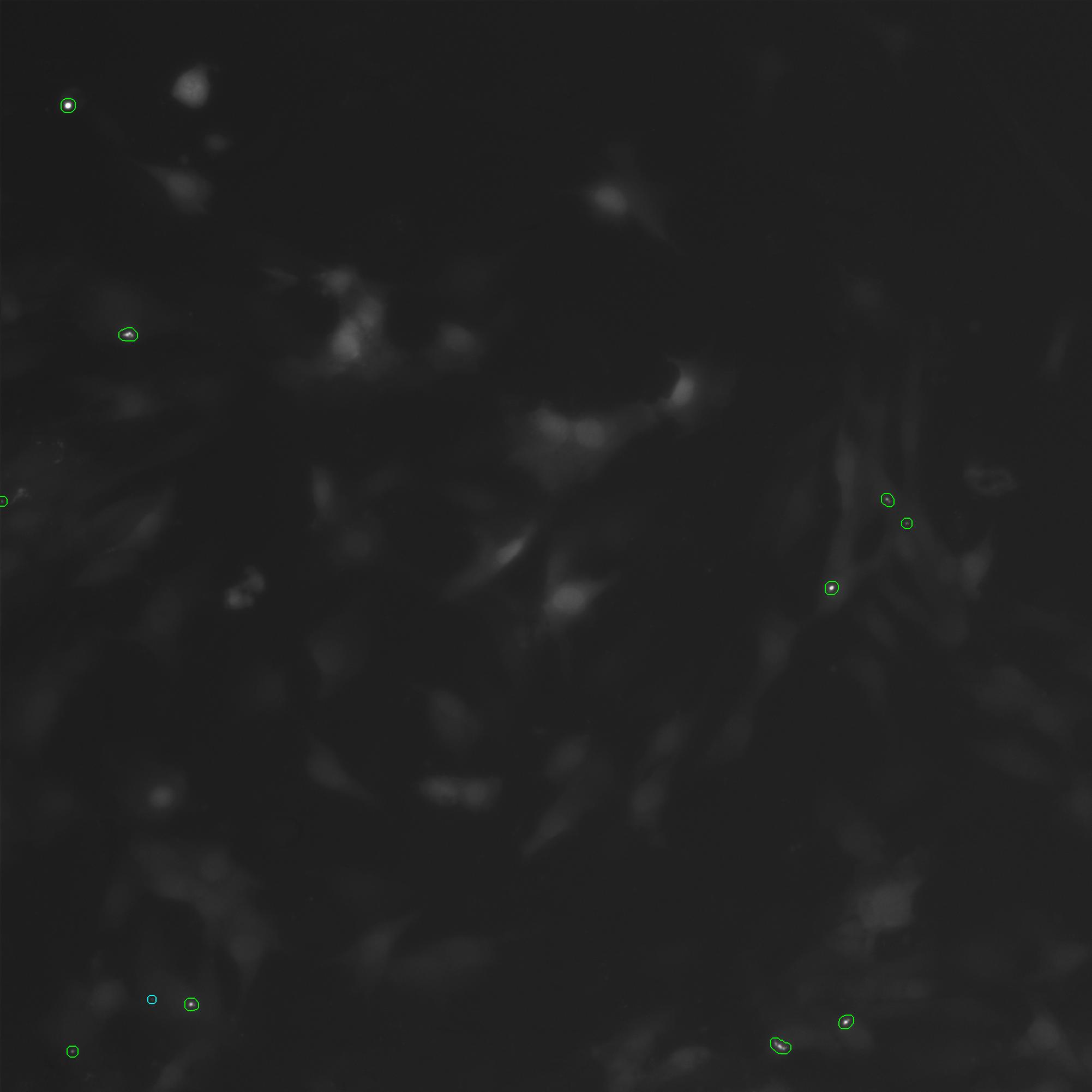

Supplement: S1 File — (ZIP) [file pone.0278130.s006.zip › Supporting Information_Matlab/ExampleData/ScreenWells/AnalyseImages/E04_025_aggr.jpg]

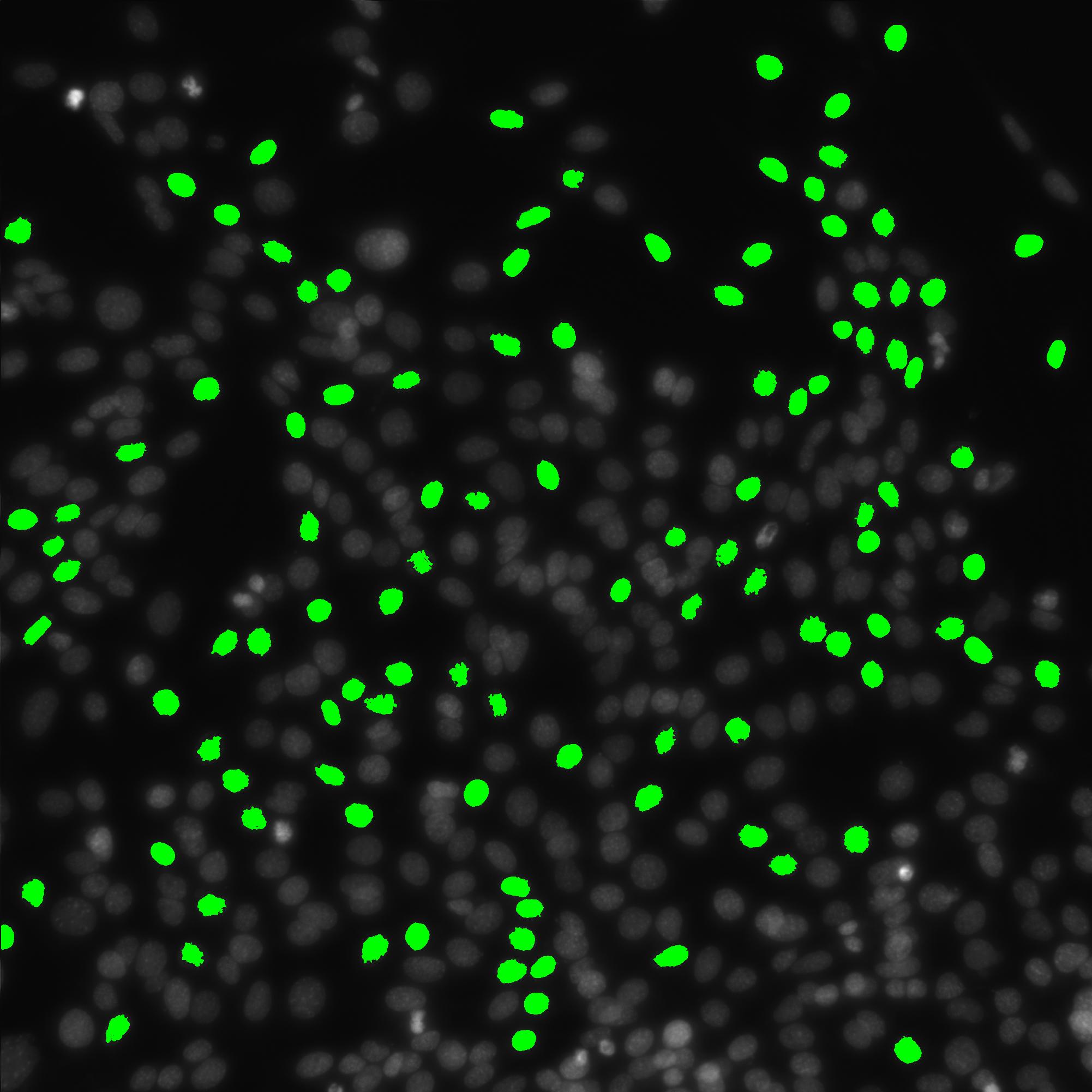

Supplement: S1 File — (ZIP) [file pone.0278130.s006.zip › Supporting Information_Matlab/ExampleData/ScreenWells/AnalyseImages/E04_025_singlenucl.jpg]

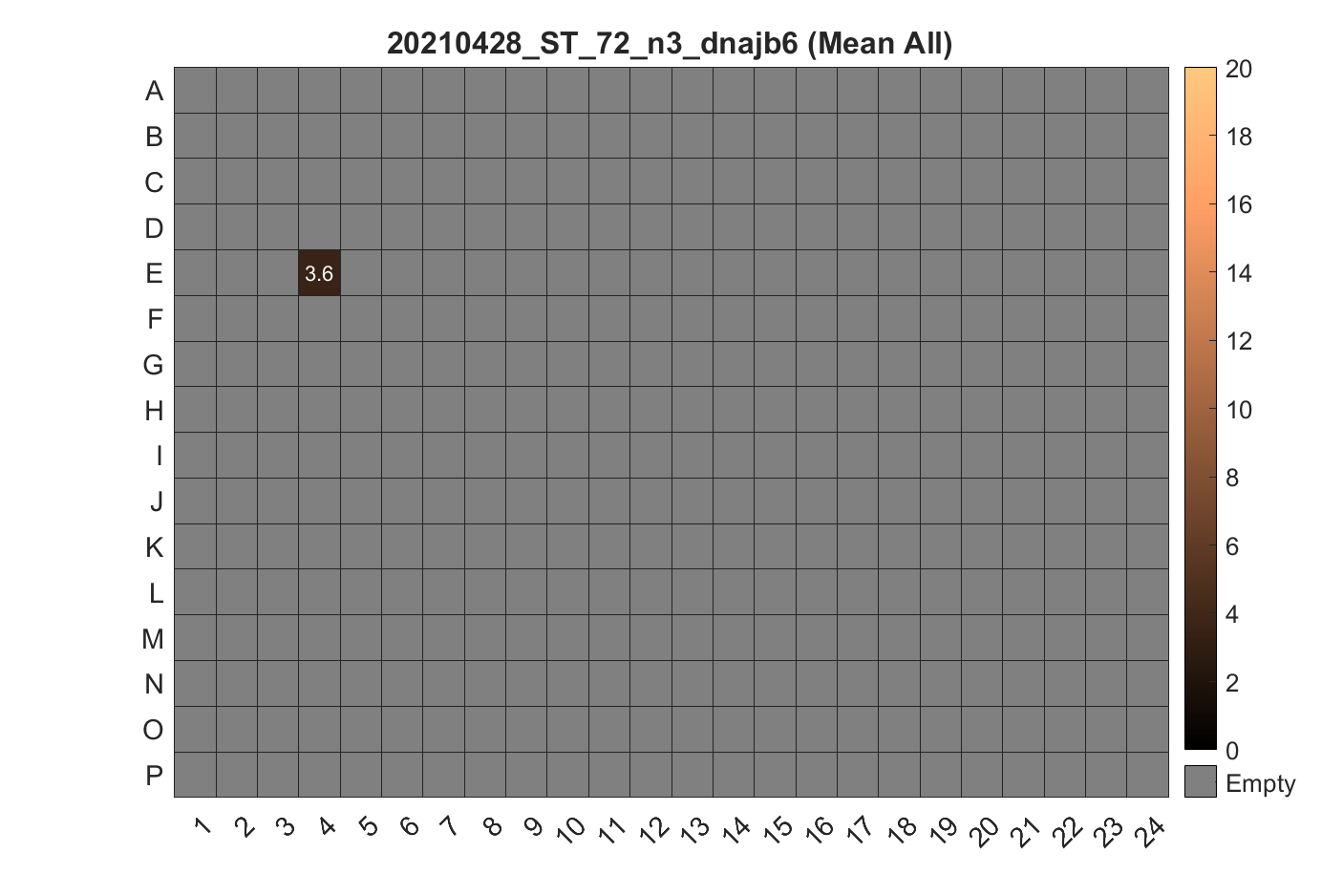

Supplement: S1 File — (ZIP) [file pone.0278130.s006.zip › Supporting Information_Matlab/ExampleData/ScreenWells/Results/heatmap-All-Tiles.png]
