# Supplementary material for: Reduction in PA28αβ activation in HD mouse brain correlates to increased mHTT aggregation in cell models
Source: PLoS One. 2022 Dec 27;17(12):e0278130. doi: 10.1371/journal.pone.0278130 (PMC9794069; doi:10.1371/journal.pone.0278130)
Supplement: S1 File — (ZIP) [file pone.0278130.s006.zip › Supporting Information_Matlab/PicoScreen.pdf]

Pico Screen for Aggregates in Cells

Load images

```
% Define Images
NucleiFile='20210428_ST_72_n3_dnajb6_E04_nuclei_14.tif';
AggrFile='20210428_ST_72_n3_dnajb6_E04_aggr_14.tif';

% Load Hoechst Nuclei Image
imNuclei=imread(NucleiFile);
imNuclei=double(imNuclei)/4095; % MaxValue from Pico's is 4095
imNucleiOrg=imNuclei;
% Load GFP Aggregates Image
imAggr=imread(AggrFile);
imAggr=double(imAggr)/4095;
% Showing Nuclei Left and Aggregates Right
montage({imadjust(imNuclei,[0 0.4]), imadjust(imAggr,[0 1])});
```

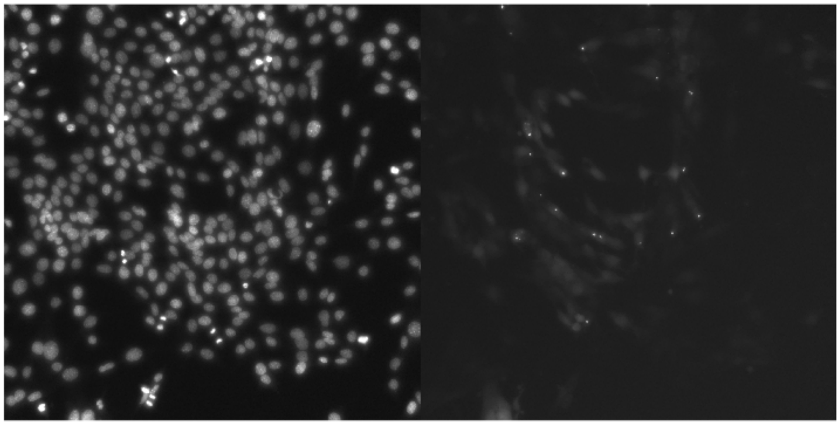

Segmentation of the Nuclei

```
% Noise Filter Nuclei Image
imNuclei=medfilt2(imNuclei,[3 3]);

% Background Correction (Rolling Ball Filter)
rolling_balln=45;
se = strel('disk', rolling_balln);
background = imopen(imNuclei, se);
imNuclei = imNuclei-background;
imNuclei(imNuclei<0)=0;
```

```
% Segment Nuclei Image using Otsu Threshold
imNucleimask=imbinarize(imNuclei,'global');
% Show Nuclei Image Left and Mask Right
montage({imadjust(imNuclei,[0 0.4]), imNucleimask});
```

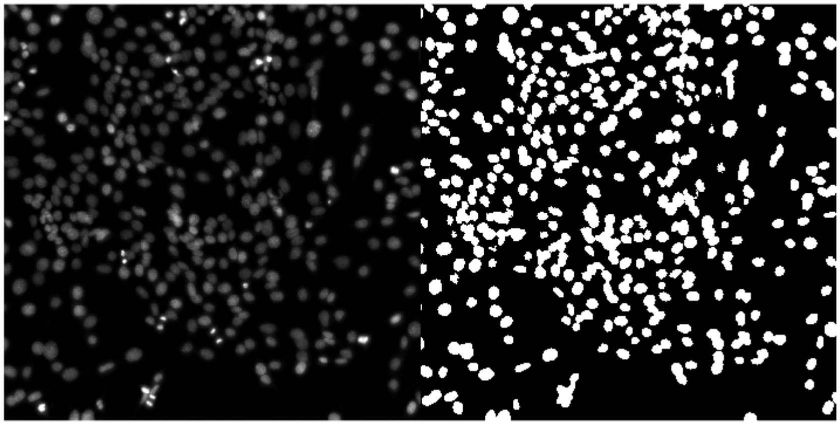

Find Overexpressing Cell Areas

```
AggrMaskO=imAggr>0.7;
AggrMaskO=bwpropfilt(AggrMaskO,imAggr,'Area',[500 20000]);
% Show Aggregates Left and Mask OverExpression Right (empty == no
overexpression)
montage({imAggr, AggrMaskO});
```

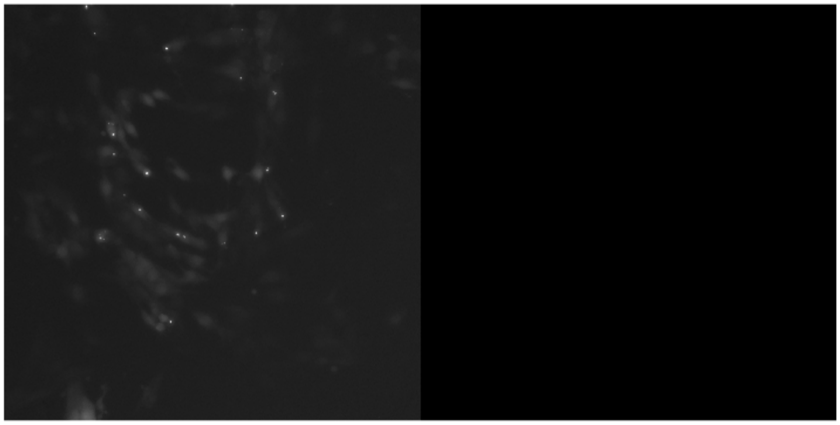

Find Aggregates

```
% Background Correction (Rolling Ball Filter)
% Size of rolling ball is set to expected Aggregate Size
rolling_ball=6;
se = strel('disk', rolling_ball);
background = imopen(imAggr, se);
imAggrC = imAggr-background;
imAggrC(imAggrC<0)=0;

% Cropping all images and masks due to border artifacts of background
subtraction
s0=floor(rolling_ball/2);
s1=size(imAggrC,1)-floor(rolling_ball/2);
s2=size(imAggrC,2)-floor(rolling_ball/2);
imAggr=imAggr(s0:s1,s0:s2);
imAggrC=imAggrC(s0:s1,s0:s2);
AggrMaskO=AggrMaskO(s0:s1,s0:s2);
imNucleimask=imNucleimask(s0:s1,s0:s2);
imNucleiOrg=imNucleiOrg(s0:s1,s0:s2);

% Create Aggregate Mask
AggrMask=imAggrC>0.1;
% Remove over GFP expressing Cells (if any)
AggrMask(AggrMaskO==1)=0;
% Color All Found Aggregates Yellow
m=(imdilate(AggrMask,strel('disk', 13))-imdilate(AggrMask,strel('disk', 7)));
mo=imoverlay(imAggr,m,'yellow');

% Filter on Expected Intensity
AggrMask=bwpropfilt(AggrMask,imAggr,'MeanIntensity',[0.2,1]);
% Filter on Expected Area
AggrMask=bwpropfilt(AggrMask,imAggr,'Area',[3, 500]);
% Color Area Filtered Aggregates Green
m=(imdilate(AggrMask,strel('disk', 13))-imdilate(AggrMask,strel('disk', 7)));
mo=imoverlay(mo,m,'green');

% Show Aggregates Left and Detected and Filtered Aggregates (Green is OK)
montage({imAggr(600:1400,300:1200,:), mo(600:1400,300:1200,:)});
```

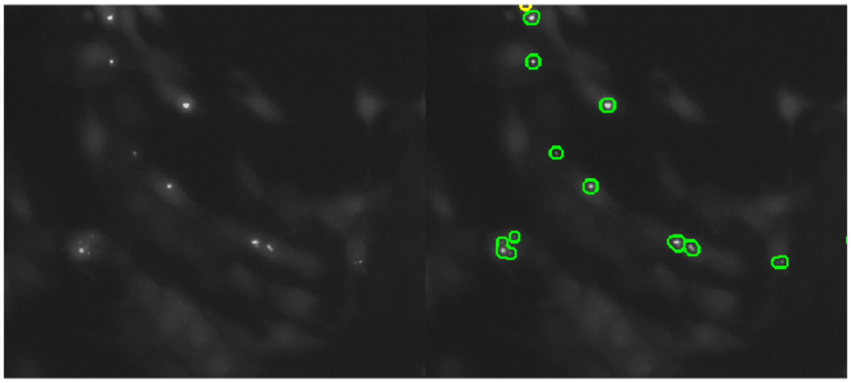

Calculating Number Single Nuclei

```
% Find Single Nuclei in Nuclei Image
% Remove Nuclei at borders
imNucleimaskc=imclearborder(imNucleimask);
% Filter on Area
imNucleimaskc=bwareafilt(imNucleimaskc,[800, 1800]);
% Find Number of Single Nuclei in Nuclei Image
cc=bwconncomp(imNucleimaskc);
SingleNucleiNumber=cc.NumObjects;
disp(['Number of Single Nuclei = ' num2str(SingleNucleiNumber)])
```

```
Number of Single Nuclei = 80
```

```
m=imoverlay(imNucleimask,imNucleimaskc,'green');
montage({imadjust(imNucleiOrg,[0 0.4]),m});
```

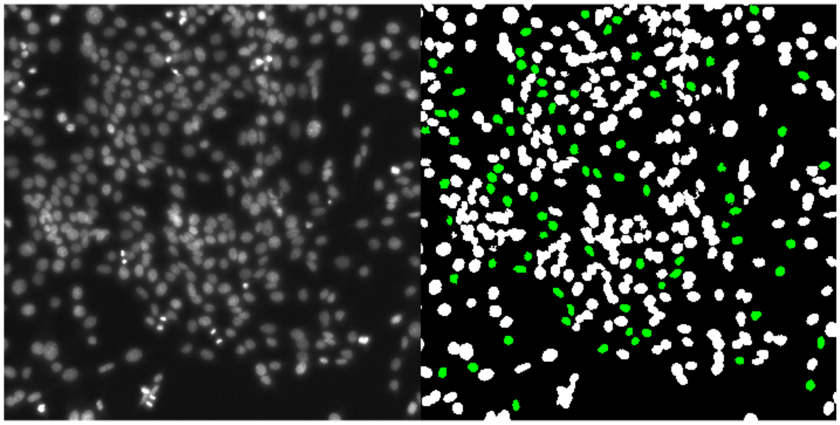

```
%Calculating AllNuclei Stats
imNucleimask(AggrMaskO==1)=0; % Exclude GFP Overexpressing Cells

% Calculating Single Nuclei Stats
if SingleNucleiNumber>=1 % At least 1 nuclei for stats
    % Select only Circular Single Nuclei

AggrStats=regionprops('table',imNucleimaskc,imNucleiOrg,'Circularity','MeanInten
sity','Area');
    AggrStats=AggrStats(AggrStats.Circularity>0.95,:);
    if ~isempty(AggrStats)
        TileSingleNucleiIntensity=AggrStats.MeanIntensity.*AggrStats.Area;
    else
        errordlg('Error Calculating Stats of Nuclei, no Circular Single Nuclei
Found')
    end
else
    errordlg('Error Calculating Number of Nuclei, no single nuclei found')
end
disp(['Number of Selected Single Nuclei = ' num2str(numel(AggrStats.Area))])
```

```
Number of Selected Single Nuclei = 23
```

Calculating Number of Nuclei

```
TileAllNucleiIntensity=sum(imNucleimask.*imNucleiOrg,'all');
MeanTileSingleNucleiIntensity=mean(TileSingleNucleiIntensity);
TileNumNuclei=round(TileAllNucleiIntensity/MeanTileSingleNucleiIntensity);
disp(['Tile Number of Nuclei = ' num2str(TileNumNuclei)]);
```

```
Tile Number of Nuclei = 593
```

Calculating Aggregate Stats

```
cc=bwconncomp(AggrMask);
TileNumberofAggr=cc.NumObjects;
disp(['Tile Number of Aggregates = ' num2str(TileNumberofAggr)])
```

```
Tile Number of Aggregates = 26
```

Percentage of Nuclei with Aggregate

```
PercTileNucleiWithAggr=TileNumberofAggr/TileNumNuclei*100;
disp(['Tile Percentage of Nuclei with Aggregate = '
num2str(PercTileNucleiWithAggr) '%'])
```

```
Tile Percentage of Nuclei with Aggregate = 4.3845%
```

For multiple Images per Well:

In the case of multiple images per well, the sum of the aggregates per image is divided to the total number of nuclei.

PseudoCode for Percentage Aggragates in Well:

WellMeanSingleNucleiIntensity=mean(TileSingleNucleiIntensities);

WellAllNucleiIntensity=sum(TileAllNucleiIntensities);

WellNumNuclei=round(MeanAllNucleiIntensity/WellAllNucleiIntensity);

WellNumAggr=sum(TileAggrNumbers);

*PercWellNucleiWithAggr=WellNumAggr/WellNumNuclei*100;*
